# Total Synthesis of Voratin C: Formation of the [6,5]-Spiroketal Core through Acid-Mediated Epoxide Cascade Opening

**DOI:** 10.1021/acs.orglett.6c02323

**Published:** 2026-07-02

**Authors:** Chun-Ho Ip, Sergei Ivlev, Ulrich Koert

**Affiliations:** Department of Chemistry, Philipps University of Marburg, Hans-Meerwein-Straße 4, D-35043 Marburg, Germany

## Abstract

An enantioselective total synthesis of voratin C is reported. Key steps include a ketalization through an epoxide-opening cascade to construct the spiroketal core. An enriched Sharpless dihydroxylation, followed by a stereospecific diol-to-epoxide conversion, furnished the key epoxide intermediate. The carbon skeleton of the natural product was assembled in a cross-olefin metathesis between a vinylpyridine and a C10–C18 alkene fragment. The C3 stereocenter was established via an enantioselective Cu-mediated conjugate reduction of an acrylonitrile.

## Graphical abstract

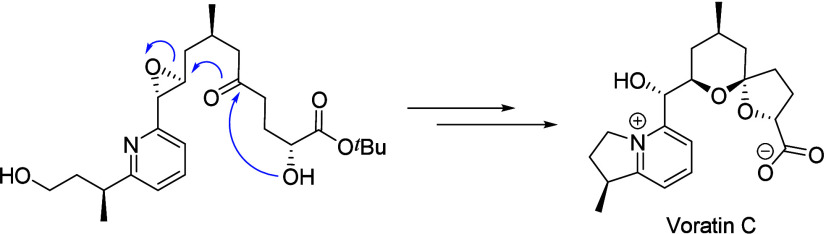

Bioactive natural products with spiroketal units are numerous.^1^ Pyridinium alkaloids form a small but well-studied natural product subclass.^2^ Spiroketals^3^ and pyridinium alkaloids^4^ continue to attract the interest of synthetic and natural product chemists. Examples of natural products with a spiroketal unit and a pyridinium substructure are very rare. Recent, prominent examples are the voratins.^3^ Voratins A (**1**), B (**2**), and C (**3**) (Figure 1) were isolated from the marine dinoflagellate *Effrenium voratum* and show inhibitory effects on biomarkers for benign prostatic hyperplasia.^5^ All three zwitterionic compounds contain a dihydroindolizinium ring and a spiroketal substructure, a 6,6-spiroketal for voratins A (**1**) and B (**2**) and a 6,5-spiroketal for voratin C (**3**).

**Figure 1:**
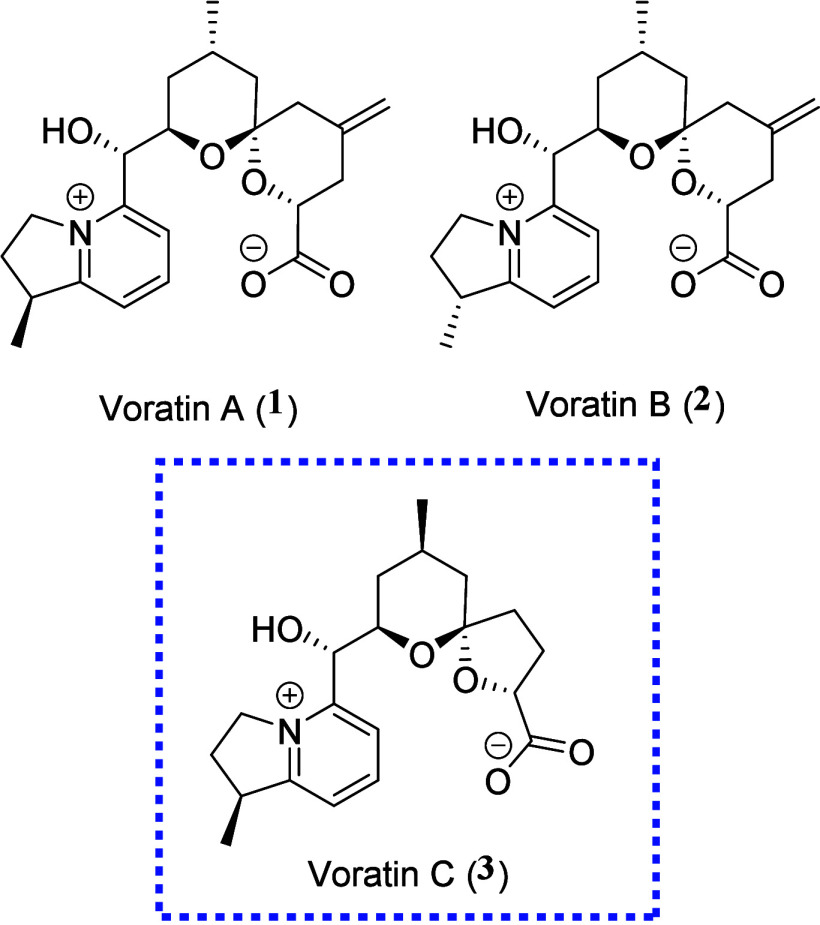
Reported structures of voratins A (**1**), B (**2**), and C (**3**).

The unique combination of structural features and the promising bioactivity makes the voratins rewarding synthetic targets. Herein, we report the first total synthesis of natural product voratin C (**3**). Our retrosynthesis (Scheme 1) is inspired by the Cane–Celmer–Westley hypothesis for polyether biosynthesis.^6^ The spiroketal part of voratin C (**3**) could be prepared by a cascade of ketalization and epoxide opening from an epoxy–ketone intermediate of type **4**. Alkene **5** would be a suitable precursor for this epoxide. A cross-olefin metathesis of vinylpyridine **6** and terminal alkene **7** should be a key step to assemble the whole carbon skeleton of the target compound.

**Scheme 1:**
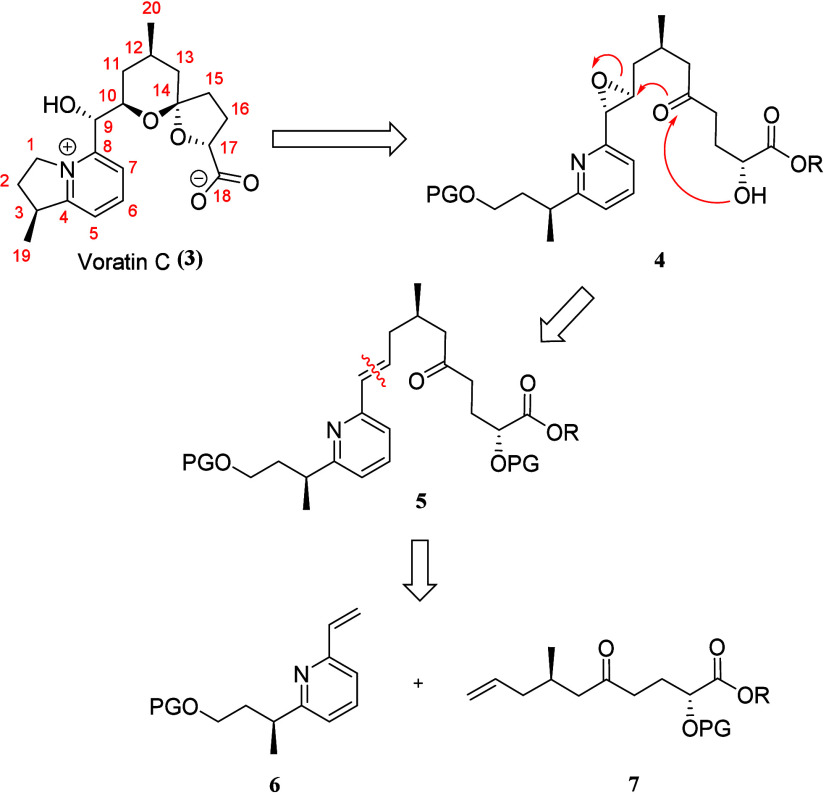
Retrosynthesis of Voratin C (**3**)

The main challenge in the synthesis of the vinyl pyridine building block **6** is the controlled introduction of the C3 stereocenter with its methyl group. Methods that were developed for aryl systems turned out to be problematic for the present pyridine case. For example, Knochel’s stereoretentive C­(sp^3^)–C­(sp^2^) cross-coupling of chiral secondary alkylzinc reagents^7^ proved to be completely racemic for the **8** + **9** → **10** case (Scheme 2A). The Evans auxiliary-controlled hydrogenation of alkene **11** gave a low diastereoselectivity (Scheme 2B). However, access to hydrogenation products of type **12** provided crystalline material, which allowed the determination of the absolute configuration of the new stereocenter confirmed by X-ray (see the Supporting Information).

**Scheme 2:**
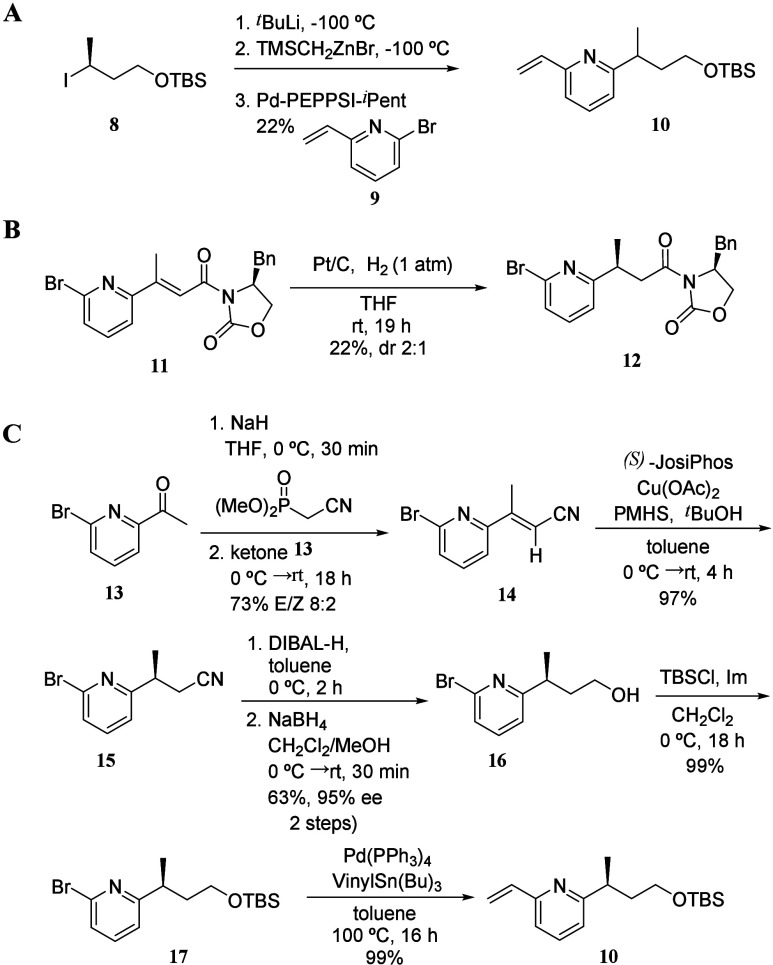
Stereoselective Synthesis of Vinyl Pyridine **10**

The starting point for the enantioselective synthesis of pyridine **10** was bromopyridine **13**, which was converted into acrylonitrile **14** (Scheme 2C). The Cu-mediated asymmetric conjugate reduction of **14** using polymethylhydrosiloxane (PMHS) as a hydride source and (*S*)-JosiPhos as a chiral ligand gave desired saturated nitrile **15** in excellent yield and enantioselectivity (Scheme 2C).^8^ The enantioselectivity and absolute configuration of the new stereocenter in **15** could be established at the stage of alcohol **16**, which was correlated with the outcome of the Evans auxiliary series (see the Supporting Information). Conversion of nitrile **15** into primary alcohol **16** and subsequent TBS protection delivered bromopyridine **17**. A final Stille coupling gave desired enantiopure building block **10**.

The synthesis of C10–C18 segment **7** as TBS-ether **24** (Scheme 3) started with commercially available carboxylic acid **18**, which can also be prepared from inexpensive d-glutamic acid. (*R*)-5-Oxo-tetrahydrofuran-2-carboxylic acid (**18**) was converted into *tert*-butyl ester **19** (Scheme 3).^9^ Conversion of butyrolactone **19** into Weinreb amide **20** proceeded smoothly. Reaction of the latter protected Weinreb amide **21** with Grignard compound **23** gave desired ketone **24**. Grignard reagent **23** was prepared from known bromide **22**, which can be accessed by the Evans allylation methodology.^10^

**Scheme 3:**
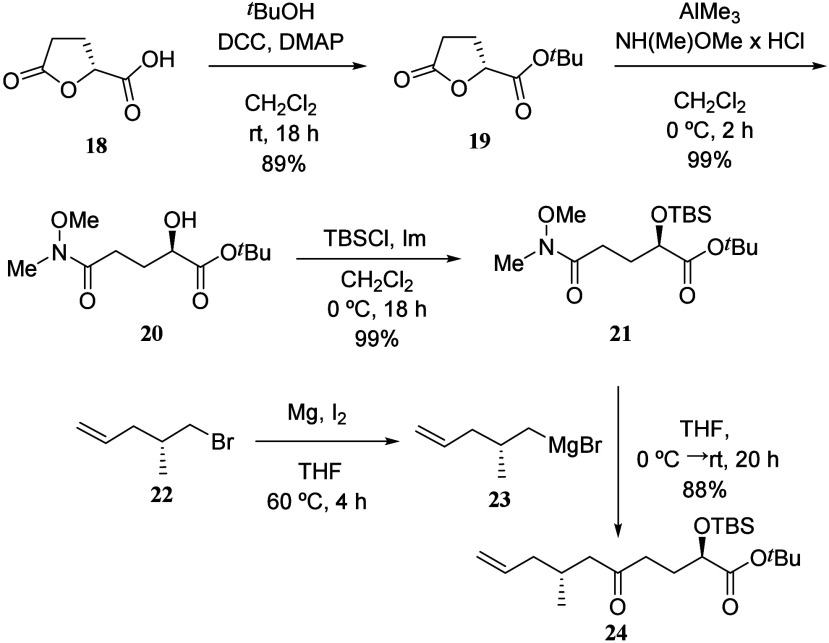
Stereoselective Synthesis of Ketone **24**

The cross-olefin metathesis of building blocks **10** and **24** had to be addressed next (Scheme 4). 2-Vinylpyridines have been used rarely in such a type of intermolecular cross-olefin metathesis reactions.^11^ For the present case, larger amounts (at least 10%) of Grubbs–Hoveyda II catalyst were necessary to give a reasonable yield of desired alkene **25** (Scheme 4). For small scale reactions (0.5 mmol), up to 65% was obtained, and upscaling (0.6–1.0 mmol) resulted in a variation of the yield by 30% in some cases only. The homodimer formation of **24** was observed as a significant side reaction. Subsequent attempts to perform the stereoselective epoxidation of alkene **25** were disappointing. Neither with Jacobsen’s^12^ nor with Shi’s^13^ epoxidation conditions was a satisfying amount of epoxide **26** formed. The presence of the pyridine N atom seems to slow the catalytic activity for these epoxidation reactions. Low turnover was observed, and if harsher conditions were applied, the formation of the pyridine *N*-oxide was a serious side reaction.

**Scheme 4:**
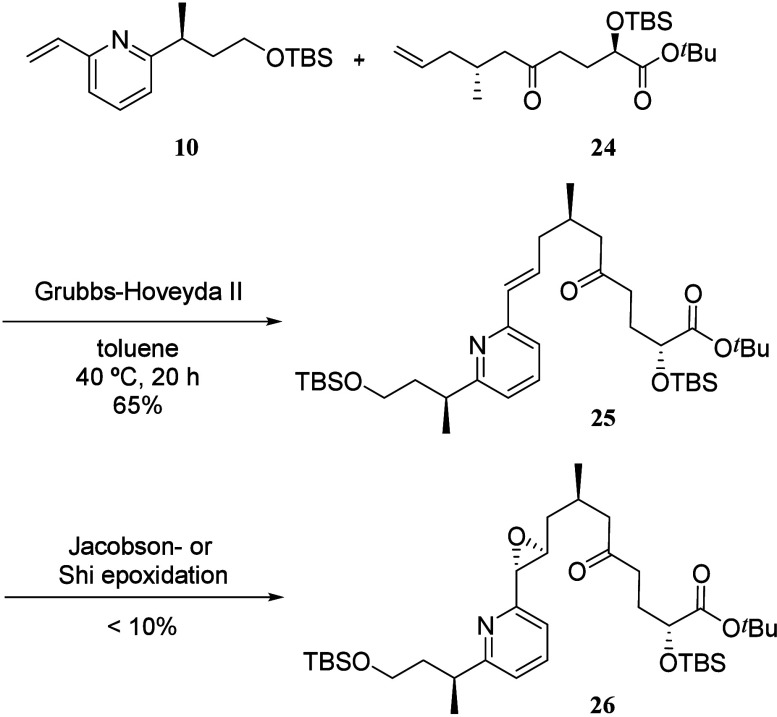
Successful Cross-Olefin Metathesis and Subsequent Epoxidation Attempts

While the direct stereocontrolled epoxidation of alkene **25** was unsuccessful, we turned our attention to a two-step sequence of asymmetric dihydroxylation and subsequent stereospecific epoxide formation (Scheme 5).^14^ Initial attempts using commercially available AD-mix-α showed no conversion (Table 1, entry 1). Increasing the amount of osmate led to a good yield of diol **27** but without stereoselectivity (entry 3). Enriching AD-mix-α further by adding chiral ligand (DHQ)_2_PHAL and an excess of K_2_CO_3_/K_3_[Fe­(CN)_6_] led to an excellent diastereoselectivity and a satisfying yield of diol **27** (entries 4 and 5).

**Scheme 5:**
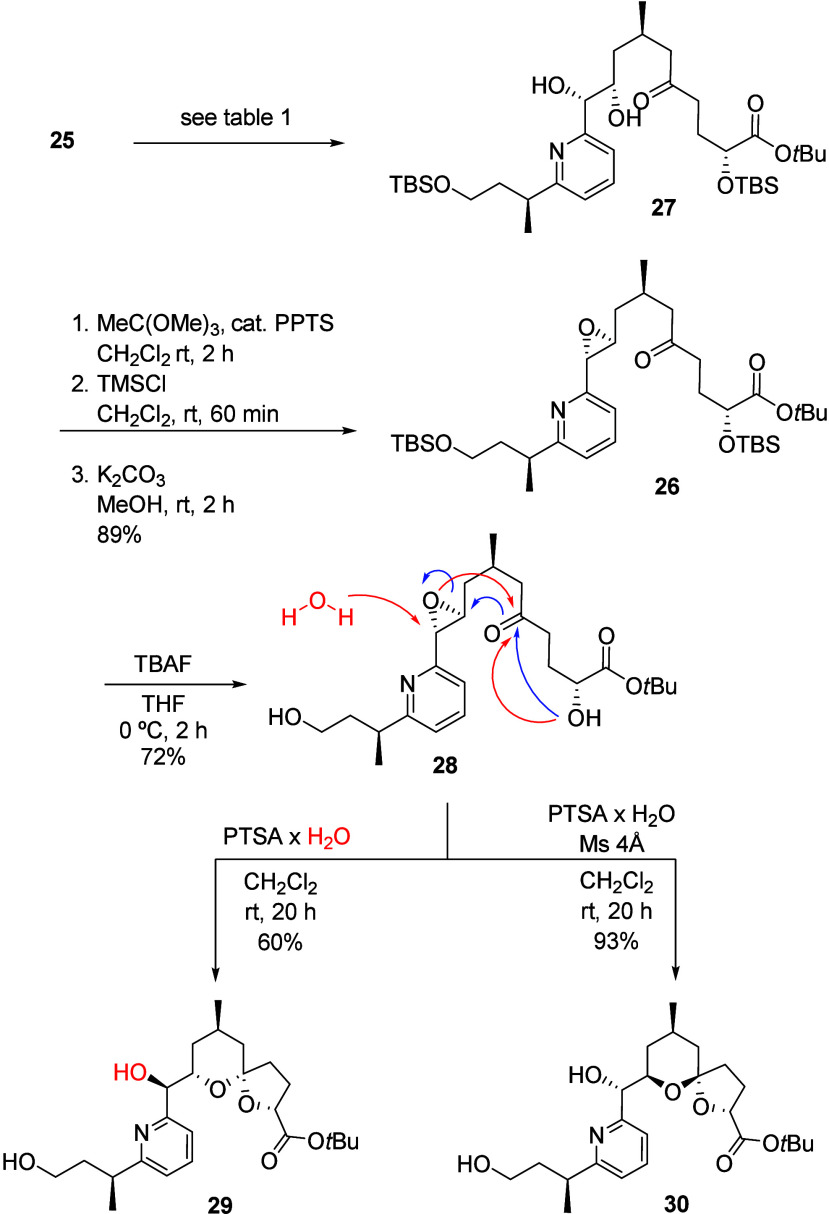
Stereoselective Synthesis of Key Epoxide **26** and Its Acid-Mediated Ketalization/Epoxide Cascade

**Table 1:** Optimization of the Asymmetric Dihydroxylation of Alkene **25** and Variations of the Standard Conditions^a^

	K_2_[OsO_2_(OH)_4_] (equiv)	(DHQ)_2_PHAL (equiv)	additive	yield
1^b^	–	–	–	no reaction
2^b^	–	–	MeSO_2_NH_2_ (1.0 equiv)	no reaction
3^c^,^d^	0.3	–	MeSO_2_NH_2_ (1.0 equiv)	72%, dr 1:1
4^c^,^d^	0.2	0.1	MeSO_2_NH_2_ (1.1 equiv)	67%, dr 1:0.4
5^c^,^d^	0.2	0.4	MeSO_2_NH_2_ (1.1 equiv)	64%, dr >25:1

a Standard conditions: K_2_[OsO_2_(OH)_4_] (0.5 mol %), (DHQ)_2_PHAL (1.0 mol %), K_2_CO_3_ (3.0 equiv), K_3_[Fe­(CN)_6_] (3.0 equiv), 1:1 *t*BuOH/H_2_O (0.075 M), 0 °C to rt, 18 h.

b Commercially purchased AD-mix-α.

c Self-prepared AD-mix-α.

d The diastereomeric ratio was determined by integration of the ^1^H NMR spectrum.

Twofold TBS deprotection of **26** gave epoxydiol **28**. The acid-initiated epoxide opening cascade^15^ of epoxide **28** provided some surprise in the beginning. Using commercially available *p*TsOH monohydrate epoxide **28** gave spiroketal **29** with the expected constitution but a relative configuration different from that of the natural product. Its relative configuration was assigned by the key H10–H20, H13–H15, H9–H11a, H10–H11b, and H10–H17 NOE correlations (Figure 2). This result could be rationalized by an intermolecular attack of water at the quasi-benzylic position (Scheme 5, red arrows). Thus, the strict exclusion of water was secured by molecular sieves. Now, the acid-initiated epoxide-opening cascade followed the planned blue arrows and delivered spiroketal **30** with a relative configuration in agreement with voratin C (**3**). The relative configuration was assigned on the basis of the key H12–H20, H9–H17, H9–H11b, H10–H11a, and H13–H15 NOE correlations (Figure 2) and by the correlation with the spiroketal system of the natural product.

**Figure 2:**
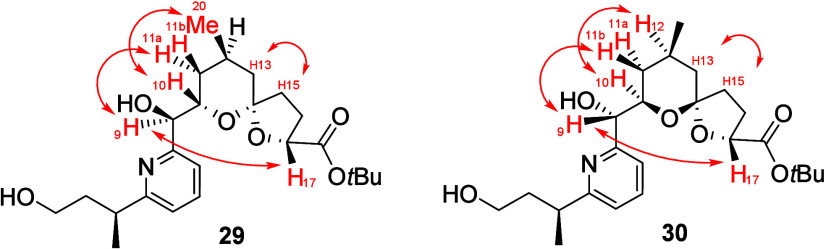
Key NOE correlations of **29** and **30** for the assignment of the relative configuration.

The final stage of the synthesis of voratin C (**3**), including release of the zwitterionic form, is shown in Scheme 6. Mesylation of the primary alcohol enabled direct intramolecular cyclization to afford the dihydroindolizinium ring with mesylate as the counterion. Subsequent cleavage of the *tert*-butyl ester with TFA provided the corresponding carboxylic acid, furnishing voratin C (**31**) as its TFA salt. The presence of TFA was confirmed by ^19^F NMR spectroscopy, and the downfield shift of H17 is consistent with a strongly deshielding counterion environment. Counterion exchange from trifluoroacetate to chloride, followed by formate, and removal via repeated lyophilization delivered voratin C (**3**). The analytical data matched those of the natural isolate (see the Supporting Information). The optical rotation of the synthetic sample ([α]^25^_D_ = +8.9 (*c* 0.07, MeOH)) agrees with that of the natural isolate sample ([α]^25^_D_ = +7.0 (*c* 0.07, MeOH)).^5^

**Scheme 6:**
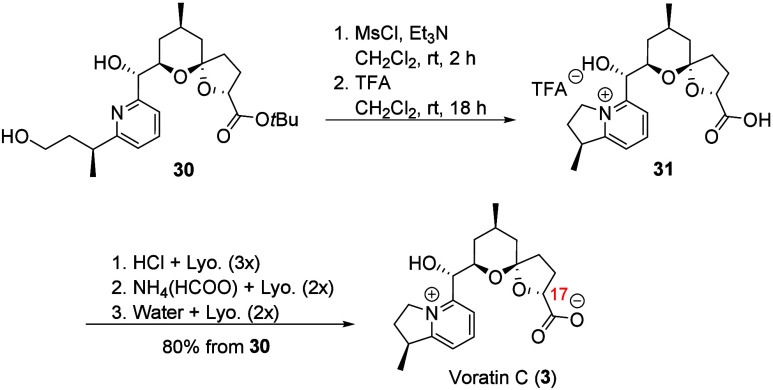
Synthesis of Voratin C (**3**) and Counterion Removal

In conclusion, the first enantioselective total synthesis of voratin C (**3**) was achieved in 15 steps (longest linear sequence) with an overall yield of 9%. A key step is the acid-promoted polyether spiroketalization via an epoxide cascade opening of epoxy–ketone **28**. The carbon skeleton was assembled in a cross-metathesis of vinylpyridine **10** and terminal alkene **24**. In addition, an asymmetric dihydroxylation–epoxide formation sequence was used to prepare the key epoxide. This strategy could enable access to other pyridinium-containing natural products, including related voratins A (**1**) and B (**2**), which are currently under investigation and may facilitate further biological and synthetic studies of this natural compound class.

## Supplementary Material



## Data Availability

The data underlying this study are available in the published article and its Supporting Information.

## Notes

Experimental details, spectroscopic data, and analytical data of all new compounds (PDF)
