# Supplementary material for: Total Synthesis of Voratin C: Formation of the [6,5]-Spiroketal Core through Acid-Mediated Epoxide Cascade Opening
Source: Org Lett. 2026 Jul 2;28(28):8934–8. doi: 10.1021/acs.orglett.6c02323 (PMC13386527; doi:10.1021/acs.orglett.6c02323)
Supplement: Supplementary file 1 [file ol6c02323_si_001.pdf]

Supporting Information

Total Synthesis of Voratin C: Formation of [6,5]-Spiroketal Core through Acid Mediated Epoxide-Cascade-Opening

Chun-Ho Ip, Sergei Ivlev, Ulrich Koert*

Department of Chemistry, Philipps-Universität Marburg, Hans-Meerwein-Strasse 4, D-35043 Marburg, Germany

Email: koert@chemie.uni-marburg.de

Table of contents

1 General Methods and Materials 2

2 Experimental procedures & Characterization Data 4

3 NMR-Spectra 27

4 Establishment of the C3 Configuration 60

4.1 Experimental procedures & Characterization Data 60
4.2 NMR-Data 73

5 X-Ray Data 80

*5.1 X-Ray Data of (S,R-)**12** 80*

6 References 82

1 General Methods and Materials

All non-aqueous reactions were carried out using flame-dried glassware under argon atmosphere unless noted otherwise. All solvents were distilled by rotary evaporation. Solvents for non-aqueous reactions were dried as follows prior to use: THF was dried with KOH and subsequently distilled from Solvona® under nitrogen atmosphere. CH_2Cl_2 was distilled from CaH_2. MeOH was dried by refluxing with Mg-turnings (5 g/L) and subsequent distillation under nitrogen atmosphere. Toluene was distilled from Solvona® under nitrogen atmosphere. Et_2O was dried and distilled from Sodium/Solvona® under nitrogen atmosphere. An oil bath was used as a heat source for reactions that require heating. All commercially available reagents and reactants were used without purification unless otherwise noted.

Reactions were monitored by thin layer chromatography (TLC) using Merck Silica Gel 60 F_{254}-plates and visualized by fluorescence quenching under UV-light. In addition, TLC-plates were stained using a potassium permanganate or ceric sulfate/phosphomolybdic acid stain.

Chromatographic purification of products was performed on Merck Silica Gel 60 (230-400 mesh) unless otherwise noted using a forced flow of eluents. Concentration under reduced pressure was performed by rotary evaporation at 40 °C and appropriate pressure and by exposing to fine vacuum at room temperature if necessary.

NMR spectra were recorded on a Bruker AV 300 MHz, AV III 500 MHz, AV III HD 500 MHz spectrometer at room temperature unless otherwise noted. Chemical shifts are reported in ppm with the solvent resonance as internal standard. Data are reported as follows: s = singlet, d = doublet, t = triplet, q = quartet, quint = quintet, m = multiplet. Structural assignments were made with additional information from gCOSY, gHSQC, gHMBC and NOE experiments.

Mass spectra were recorded by the mass service department of the Philipps-Universität Marburg. HR-ESI mass spectra were acquired with an LTQ-FT mass spectrometer (Thermo Fischer Scientific). The resolution was set to 100 000.

IR spectra were recorded on a Bruker IFS 200 spectrometer. The absorption bands are given in wave numbers (cm^{-1}), intensities are reported as follows: s = strong, m = medium, w = weak, br = broad band.

Melting points were determined on a Mettler Toledo MP70 using one end closed capillary tubes.

Specific rotations were determined at 20 °C for the Na-D wavelength (589 nm) with a Krüss P8000 T polarimeter.

CD spectra were recorded on a JASCO J-810 CD spectropolarimeter (500-200 nm, 1 nm band width, 50 nm/min scanning speed, accumulation of 3 scans).

UV-Visible absorption spectroscopy was performed with a Cary 8454 UV-Vis spectrophotometer from Agilent with ChemStation software. Quartz cells with cell paths of 0.2 cm was used.

HPLC chromatograms were recorded with an Agilent 1260 Infinity Series HPLC. Detection was carried at 25 °C with a DAD 3000 detector using different wavelengths. As a stationary phase, a Chiralcel OD-H column (4.6 × 250 mm) was used. The used solvents for the mobile phase were A: *n*-hexane and B: isopropyl alcohol.

RP HPLC: Purification of Voratin C (**3**) was performed using a Thermo Fisher Scientific Dionex Ultimate 3000 HPLC System. Detection was carried out at 25 °C with a DAD 3000 detector using different wavelengths. For analytical RP-HPLC, an ACE UltraCore Super 2.5 column (150 × 2.1 mm, 2.5 µm, 2.5 µm) was used. For semi-preparative ACE SuperC18 (150 × 10 mm, 5 µm, 90 Å) column was used. The used solvents for the mobile phase were A: Water + 0.1% TFA and B: MeCN + 0.085% TFA. Samples were dissolved in a mixture of solvents A and B prior to injection.

Emission and UV/Vis spectra were recorded on a SpectraMax M5 (Molecular Devices).

2 Experimental procedures & Characterization Data

2.1 (*E*)-3-(6-bromopyridin-2-yl)but-2-enenitrile (14)

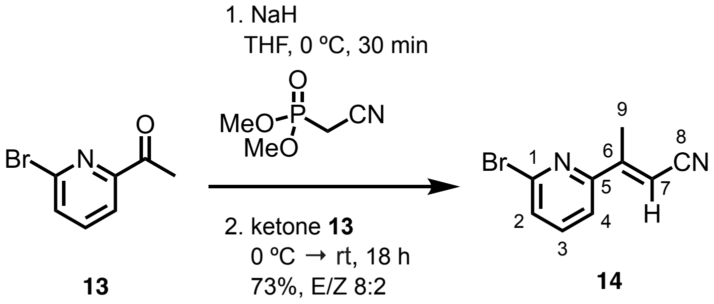

13 **14**

NaH (2.39 g, 60.0 mmol, 1.20 eq.) was added portionwise into a solution of dimethyl (cyanomethyl)phosphonate (9.70 mL, 60.0 mmol, 1.20 eq.) in THF (200 mL) at 0 °C and stirred for 30 min at the same temperature. Acetylpyridine **13** (10 g, 50.0 mmol, 1.00 eq.) was added slowly. After vigorous stirring for 18 h, sat. aq. NH_4Cl (100 mL) was added. The phases were separated and the aqueous layer was extracted with EtOAc (3x70 mL). The combined organic layers were washed with brine, dried over anhydrous $MgSO_4$ and the solvent was removed under reduced pressure. The crude product was purified with column chromatography (*n*-pentane/EtOAc 10:1→7:1) to give *E*-acrylonitrile **14** (8.00 g, 36.3 mmol, 73%) as a white solid.

TLC: R_f = 0.4 (*n*-pentane/EtOAc 7:1). **^{1}H-NMR:** (500 MHz, $CDCl_3$) δ = 7.61 (t, 1H, *J* = 7.7 Hz, *H-3*), 7.49 (d, 1H, *J* = 7.7 Hz, *H-2*), 7.44 (d, 1H, *J* = 7.7 Hz, *H-4*), 6.49 (s, 1H, *H-7*), 2.46 (s, 3H, *H-9*) ppm. **^{13}C-NMR:** (500 MHz, $CDCl_3$) δ = 155.1 (*C-6*), 155.0 (*C-8*), 142.3 (*C-1*), 139.3 (*C-3*), 129.3 (*C-2*), 119.6 (*C-4*), 117.4 (*C-5*), 100.2 (*C-7*), 18.3 (*C-9*) ppm. **HR-MS (ESI$^+$):** m/z calc. $C_9H_7BrN_2H$ $[M+H]^+$: 222.9865, found: 222.9862 m/z. **FT-IR:** film, $\tilde{\nu}$ = 3064 (w), 2998 (w), 2927 (w), 2214 (s), 2164 (w), 1980 (w), 1797 (w), 1687 (w), 1613 (w), 1567 (m), 1549 (s), 1432 (w), 1413 (s), 1380 (w), 1313 (w), 1282 (w), 1230 (w), 1166 (w), 1132 (s), 1098 (w), 1044 (w), 1015 (w), 984 (w), 938 (w), 847 (w), 788 (s), 729 (w), 693 (w), 633 (w), 615 (w), 489 (w), 452 (w) cm^{-1}. **m.p** T_m= 81-84 °C (EtOAc).

2.2 (*S*)-3-(6-bromopyridin-2-yl)butanenitrile (15)

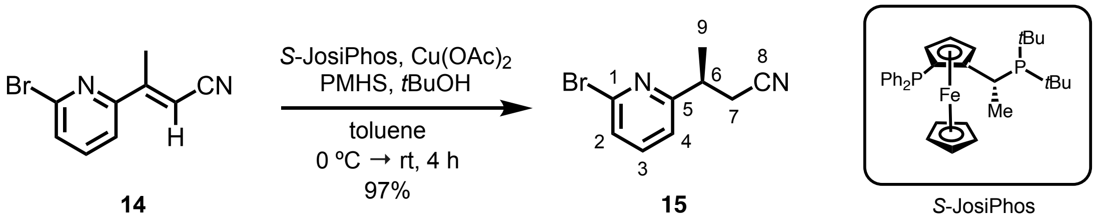

PMHS (3.87 mL, 62.7 mmol, 4.00 eq.) was added into a solution of *S*-JosiPhos (170 mg, 0.31 mmol, 0.02 eq.) and $Cu(OAc)_2$ (57.0 mg, 0.31 mmol, 0.02 eq.) in toluene (16.0 mL) at 0° C and stirred for 10 min. At 0° C a solution of acrylonitrile **14** (3.5 g, 15.7 mmol, 1.00 eq.) in toluene (16.0 mL) was added dropwise followed by *t*BuOH (5.96 mL, 62.7 mmol, 4.00 eq.) and allowed to warm up to room temperature over 20 min. After 3.5 h the reaction mixture was diluted with Et_2O (10 mL) and NaOH-solution (2 M, 50 mL) and stirred for 30 min. The phases were separated and the aqueous layer was extracted with EtOAc (3x50 mL). The combined organic layers were dried over anhydrous $MgSO_4$ and the solvent was removed under reduced pressure. The crude product was purified with column chromatography (*n*-pentane/EtOAc 10:1) to give compound **15** (3.43 g, 15.21 mmol, 97%) as a colorless oil. The *ee* could not be determined with chiral HPLC at this stage and was determined at the stage of alcohol *S*-**16** (see 2.4).

TLC: R_f = 0.5 (*n*-pentane/Et_2O 5:1). **^{1}H-NMR:** (500 MHz, $CDCl_3$) δ = 7.52 (t, 1H, *J* = 7.6 Hz, *H-3*), 7.37 (d, 1H, *J* = 7.6 Hz, *H-2*), 7.19 (d, 1H, *J* = 7.6 Hz, *H-4*), 3.29-3.22 (m, 1H, *H-6*), 2.82 (dd, 1H, *J* = 16.7, 6.6 Hz, *H-7*), 2.72 (dd, 1H, *J* = 16.7, 6.6 Hz, *H-7*), 1.44 (d, 3H, *J* = 6.9 Hz, *H-9*) ppm. **^{13}C-NMR:** (500 MHz, $CDCl_3$) δ = 163.2 (*C-8*), 142.2 (*C-1*), 139.3 (*C-3*), 126.9 (*C-2*), 120.7 (*C-4*), 118.7 (*C-5*), 38.2 (*C-6*), 24.1 (*C-7*), 20.0 (*C-9*) ppm. **HR-MS (ESI$^+$):** m/z calc. $C_9H_9BrN_2H$ $[M+H]^+$: 225.0022, found: 225.0025 m/z. **FT-IR:** film, $\tilde{\nu}$ = 3078 (w), 2973 (w), 2934 (w), 2248 (w), 1581 (w), 1553 (s), 1458 (w), 1432 (s), 1409 (w), 1377 (w), 1350 (w), 1269 (w), 1198 (w), 1159 (m), 1126 (s), 1082 (w), 1028 (w), 985 (w), 948 (w), 908 (w), 861 (w), 794 (s), 740 (m), 676 (w), 622 (w), 539 (w), 435 (w) cm^{-1}. **S.r:** $[\alpha]_D^{20}$=+31.4 (*c* 0.5, $CHCl_3$).

2.3 (*S*)-3-(6-bromopyridin-2-yl)butanal (16a)

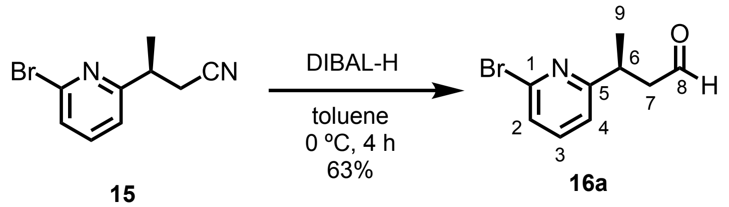

DIBAL-H (1 M in *n*-heptane, 5.33 mL, 5.33 mmol, 1.50 eq.) was added dropwise into a solution of nitrile (800 mg, 3.55 mmol, 1.00 eq.) in toluene (15 mL) at 0° C and stirred for 2 h. After completion of the reaction sat. NH_4Cl (10 mL) was added. The phases were separated, and the aqueous layer was extracted with Et_2O (3x10 mL). The combined organic layers were washed with brine, dried over anhydrous $MgSO_4$ and the solvent was removed under reduced pressure. The crude product was purified with column chromatography (*n*-pentane/EtOAc 4:1) to give aldehyde **16a** (508 mg, 2.22 mmol, 63%) as a colorless oil. Aldehyde **16a** was used directly in the next step due to its instability.

TLC: R_f = 0.43 (*n*-pentane/EtOAc 5:1). **^{1}H-NMR:** (500 MHz, $CDCl_3$) δ = 9.77 (t, 1H, *J* = 1.4 Hz, *H-8*), 7.46 (t, 1H, *J* = 7.7 Hz, *H-3*), 7.27 (d, 1H, *J* = 7.6 Hz, *H-2)*, 7.16 (d, 1H, *J* = 7.6 Hz, *H-4)*, 3.50-3.11 (m, 1H, *H-6*), 3.08 (ddd, 1H, *J* = 17.5, 6.3, 1.4 Hz, *H-7*), 2.69 (ddd, 1H, *J* = 17.5, 6.3, 1.4 Hz, *H-7*), 1.32 (d, 3H, J = 6.9 Hz, *H-9*) ppm. **^{13}C-NMR:** (500 MHz, $CDCl_3$) δ = 201.4 (*C-8*), 165.9 (*C-5*), 141.8 (*C-1*), 139.0 (*C-3*), 126.0 (*C-2*), 120.9 (*C-4*), 49.8 (*C-7*), 35.8 (*C-6*), 20.9 (*C-9*) ppm. **HR-MS (ESI$^+$):** m/z calc. $C_9H_{10}BrNOH$ $[M+H]^+$: 228.0019, found: 228.0014 m/z. **FT-IR:** film, $\tilde{\nu}$ = 3064 (w), 2968 (w), 2932 (w), 2878 (w), 2824 (w), 2724 (w), 2125 (w), 1719 (s), 1581 (s), 1552 (s), 1458 (w), 1433 (s), 1406 (m), 1355 (w), 1270 (w), 1195 (w), 1160 (m), 1125 (s), 1082 (w), 1049 (w), 985 (w), 866 (w), 795 (s), 742 (w), 656 (w), 615 (w), 535 (w), 451 (w) cm^{-1}. **S.r:** $[\alpha]_D^{20}$=+21.8 (*c* 0.5, $CHCl_3$).

2.4 (*S*)-3-(6-bromopyridin-2-yl)butan-1-ol (16)

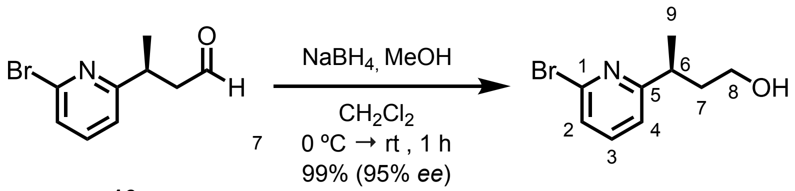

16a **16**

$NaBH_4$ (146 mg, 3.86 mmol, 2.00 eq.) was added portionwise into a solution of aldehyde (440 mg, 1.93 mmol, 1.00 eq.) in CH_2Cl_2 (13 mL) and MeOH (6.6 mL) at 0 °C. The reaction mixture was warmed up to rt and stirred for 30 min. After completion of the reaction sat. NH_4Cl (10 mL) was added. The phases were separated and the aqueous layer was extracted with EtOAc (2x50 mL). The combined organic layers were washed with brine, dried over anhydrous $MgSO_4$ and the solvent was removed under reduced pressure. The crude product was purified with column chromatography (*n*-pentane/EtOAc 1:1) to give alcohol **16** (442 mg, 1.92 mmol, 99%, 95% *ee*) as a colorless oil.

TLC: R_f= 0.24 (*n*-pentane/EtOAc 2:1). **^{1}H-NMR:** (500 MHz, $CDCl_3$) δ = 7.47 (t, 1H, *J* = 7.7 Hz, *H-3*), 7.30 (dd, 1H, *J* = 7.7, 0.8 Hz, *H-2)*, 7.13 (dd, 1H, *J* = 7.7, 0.8 Hz, *H-4)*, 3.65-3.54 (m, 2H, *H-8*), 3.12-3.08 (m, 1H, *H-6*), 2.24 (br s, 1H, O*H),* 1.95-1.87 (m, 2H, *H-7*), 1.31 (d, 3H, J = 6.9 Hz, *H-9*) ppm. **^{13}C-NMR:** (500 MHz, $CDCl_3$) δ = 167.7 (*C-5*), 141.6 (*C-1*), 139.1 (*C-3*), 125.7 (*C-2*), 120.5 (*C-4*), 60.7 (*C-8*), 39.4 (*C-7*), 38.5 (*C-6*), 20.6 (*C-9*) ppm. **HR-MS (ESI$^+$):** m/z calc. $C_9H_{12}BrNOH$ $[M+H]^+$: 230.0175, found: 230.0171 m/z. **FT-IR:** film, $\tilde{\nu}$ = 3337 (w), 2963 (w), 2931 (m), 2874 (w), 2118 (w), 1581 (m), 1552 (s), 1457 (w), 1432 (m), 1407 (s), 1373 (w), 1352 (w), 1159 (m), 1126 (m), 1082 (w), 1045 (s), 986 (m), 953 (w), 903 (w), 852 (w), 794 (s), 777 (w), 742 (m), 670 (w), 624 (w), 520 (w), 415 (w) cm^{-1}. **S.r:** $[\alpha]_D^{20}$=+21.5 (*c* 0.5, $CHCl_3$). **HPLC:** 99% *n*-hexane, 1% *i*-propanol, 0.8 mL/min, Chiralpak® OD-H (4.6x250 mm), ϑ = 20 °C, t_{major}= 43.26 min ((*S*)-**16**), t_{minor} = 44.97 min, ((*R*)-**16**), 95% *ee*.

Racemic reference:

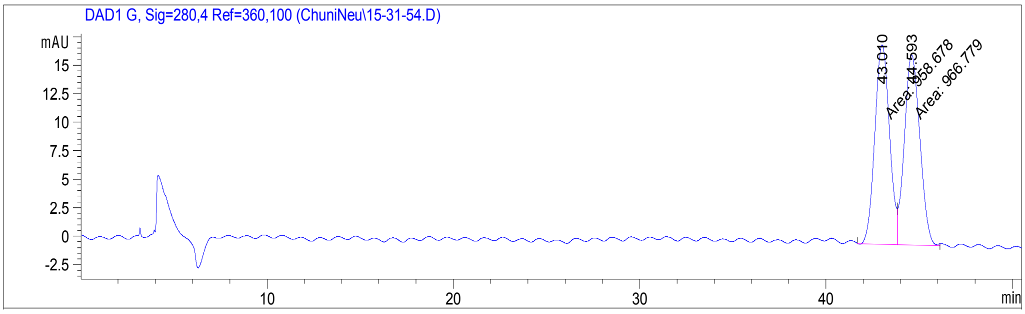

Signal 5: DAD1 G, Sig=280,4 Ref=360,100

Peak #	RetTime [min]	Type	Width [min]	Area [mAU*s]	Height [mAU]	Area %
1	43.010	MF	0.9122	958.67798	17.51561	49.7896
2	44.593	FM	0.9664	966.77875	16.67321	50.2104

Totals : 1925.45673 34.18882

(*S*)-**16**: t_R = 43.26 min, 95% *ee*

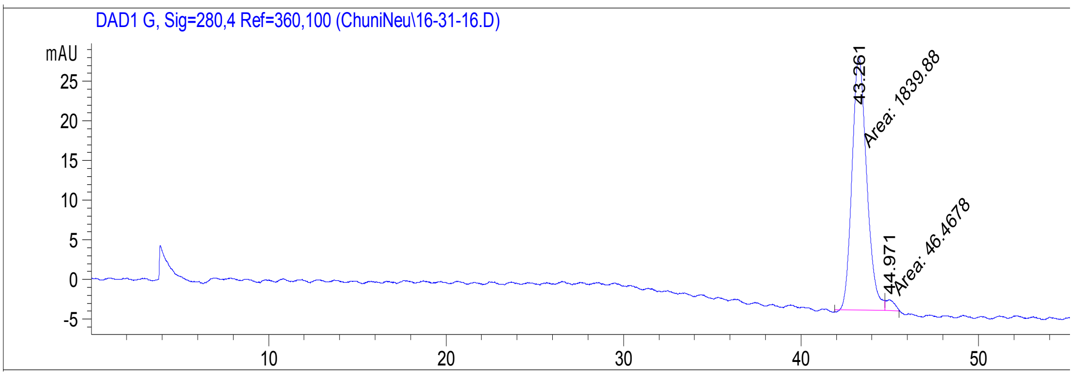

Signal 3: DAD1 G, Sig=280,4 Ref=360,100

Peak #	RetTime [min]	Type	Width [min]	Area [mAU*s]	Height [mAU]	Area %
1	43.261	MF	0.9578	1839.88354	32.01520	97.5366
2	44.971	FM	0.5604	46.46781	1.38210	2.4634

Totals : 1886.35135 33.39729

2.5 (*S*)-2-bromo-6-(4-((*tert*-butyldimethylsilyl)oxy)butan-2-yl)pyridine (17)

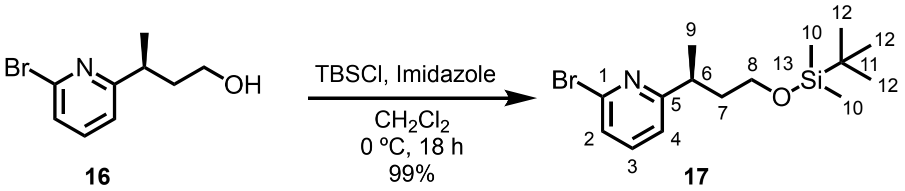

TBSCl (1.20 g, 7.909 mmol, 1.30 eq.) and imidazole (497 mg, 7.301 mmol, 1.20 eq.) were added to a solution of (*S*)-alcohol **16** (1.4 g, 6.084 mmol, 1.00 eq.) in CH_2Cl_2 (36 mL) at 0 °C. The reaction was stirred at room temperature for 20 h. After completion of the reaction distilled water (10 mL) was added and the mixture was extracted with CH_2Cl_2 (3x30 mL). The combined organic layers were washed with brine, dried over anhydrous $MgSO_4$ and the solvent was removed under reduced pressure. The crude product was purified by flash column chromatography (n-pentane/EtOAc, 20:1) to give TBS-ether 17 (2.1 g, 6.084 mmol, 99%) as a colorless oil.

TLC: R_f = 0.56 (*n*-pentane/EtOAc 20:1). **^{1}H-NMR:** (500 MHz, $CDCl_3$) δ = 7.45 (t, *J* = 7.7 Hz, 1H, *H-3*), 7.27 (dd, *J* = 7.8, 0.8 Hz, 1H, *H-2*), 7.08 (dd, *J* = 7.6, 0.9 Hz, 1H, *H-4*), 3.82-3.48 (m, 2H, *H-8*), 3.07 -3.00 (m, 1H, *H-6*), 2.01-1.95 (m, 1H, *H-7*), 1.82 (m, 1H, *H-7*), 1.26 (d, *J* = 7.0 Hz, 3H, *H-9*), 0.87 (s, 9H, *H-12*), 0.00 (d, *J* = 3.4 Hz, 6H, *H-10*) ppm. **^{13}C-NMR:** (500 MHz, $CDCl_3$) δ = 168.0 (*C-1*), 141.7 (*C-5*), 138.7 (*C-3*), 125.5 (*C-2*), 120.8 (*C-4*), 61.2 (*C-8*), 39.5 (*C-7*), 38.2 (*C-6*), 26.1 (*C-12*), 21.0 (*C-9*), 18.3 (*C-11*), -5.2 (*C-10*) ppm. **HR-MS (ESI$^+$):** m/z calc. $C_{15}H_{26}BrNOSiNa$ $[M+Na]^+$: 368.0840, found: 368.0828 m/z. **FT-IR:** film, $\tilde{\nu}$ = 2930 (w), 2858 (w), 2252 (w), 1582 (w), 1554 (w), 1462 (w), 1434 (w), 1408 (w), 1256 (w), 1161 (w), 1102 (w), 986 (w), 903 (s), 836 (w), 777 (w), 724 (s), 649 (w), 418 (w) cm-1. **S.r:** $[\alpha]_D^{20}$=+49.3 (*c* 0.5, $CHCl_3$).

2.6 (*S*)-2-(4-((*tert*-butyldimethylsilyl)oxy)butan-2-yl)-6-vinylpyridine (10)

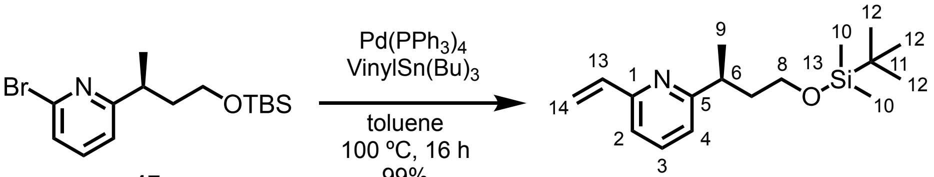

Tributyl(vinyl)tin (0.57 mL, 1.96 mmol, 1.30 eq.) was added dropwise to a solution of bromopyridine **17** (520 mg, 1.51 mmol, 1.00 eq.) and $Pd(PPh_3)_4$ (87.0 mg, 0.08 mmol, 0.05 eq.) in toluene (1 mL). The reaction mixture was stirred at 100°C for 20 h. The solvent was removed under reduced pressure. The residue was extracted with CH_2Cl_2 (3x20 mL). The combined organic layers were dried over anhydrous $MgSO_4$ and the solvent was removed under reduced pressure. The crude product was purified by flash column chromatography (*n*-pentane/EtOAc, 25:1) to obtain vinyl pyridine (*S*)-**10** (440 mg, 1.51 mmol, 99%) as a colorless oil.

TLC: R_f= 0.52 (*n*-pentane/EtOAc 25:1). **^{1}H-NMR:** (500 MHz, $CDCl_3$) δ = 7.54 (t, *J* = 7.7 Hz, 1H, *H-3*), 7.13 (dd, *J* = 7.7, 1.0 Hz, 1H, *H-2*), 7.00 (dd, *J* = 7.7, 1.0 Hz, 1H, *H-4*), 6.80 (dd, *J* = 17.5, 10.8 Hz, 1H, *H-13*), 6.19 (dd, *J* = 17.5, 1.5 Hz, 1H, *H-14*), 5.43 (dd, *J* = 10.8, 1.5 Hz, 1H, *H-14*), 3.61-3.50 (m, 2H, *H-8*), 3.05-3.01 (m, 1H, *H-6*), 2.06 (dt, *J* = 13.9, 6.7 Hz, 1H, *H-7*), 1.86 (dt, *J* = 13.5, 6.8 Hz, 1H, *H-7*), 1.28 (d, *J* = 7.0 Hz, 3H, *H-9*), 0.87 (s, 9H, *H-12*), -0.01 (s, 6H, *H-10*) ppm. **^{13}C-NMR:** (500 MHz, $CDCl_3$) δ = 165.9 (*C-5*), 155.1 (*C-1*), 137.5 (*C-13*), 136.6 (*C-3*), 120.6 (*C-4*), 118.6 (*C-2*), 117.8 (*C-14*), 61.5 (*C-8*), 39.8 (*C-7*), 38.5 (*C-6*), 26.1 (*C-12*), 21.2 (*C-9*), 18.4 (*C-11*), -5.2 (*C-10*) ppm. **HR-MS (ESI$^+$):** m/z calc. $C_{17}H_{30}NOSi$ $[M+H]^+$: 292.2102, found: 292.2084 m/z. **FT-IR:** film, $\tilde{\nu}$ = 2957 (w), 2928 (w), 2857 (w), 2253 (w), 2043 (w), 1572 (w), 1462 (w), 1378 (w), 1255 (w), 1159 (w), 1093 (w), 991 (w), 904 (s), 836 (w), 776 (w), 726 (s), 649 (w), 598 (w), 542 (w), 510 (w), 465 (w), 408 (w) cm^{-1}. **S.r:** $[\alpha]_D^{20}$=+29.3 (*c* 0.5, $CHCl_3$).

2.7 Synthesis of *tert*-butyl (*R*)-5-oxotetrahydrofuran-2-carboxylate (19)

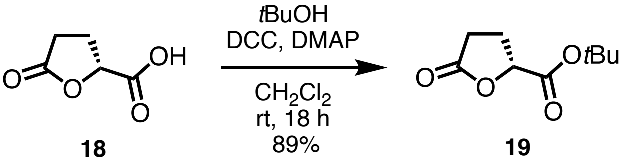

Ester **19** was prepared according to a literature procedure.[2] A solution of DCC (8.72 g, 42.27 mmol, 1.10 eq.) in CH_2Cl_2 (40 mL) was added to a solution of carboxylic acid **18** (5.0 g, 38.43 mmol, 1.00 eq.), *t*BuOH (3.97 mL, 42.27 mmol, 1.10 eq.) and DMAP (1.88 g, 15.37 mmol, 0.40 eq.) in CH_2Cl_2 (140 mL) at 0° C. The reaction mixture was stirred for 18 h. Upon completion the solvent was removed under reduced pressure. The residue was purified by column chromatography (*n*-pentane/EtOAc 3:1) to give ester **19** (6.63 g, 34.13 mmol, 89%) as a pale yellow solid. The analytical data are in accordance with the literature.[2]

TLC: R_f = 0.30 (*n*-pentane/EtOAc 3:1). **^{1}H-NMR:** (300 MHz, $CDCl_3$) δ = 4.84-4.77 (m, 1H, C*H*), 2.65-2.44 (m, 3H, C*H₂,* C*H*), 2.24 (m, 1H, *CH*), 1.48 (s, 9H, *3*xC*H₃*) ppm.

2.8 *tert*-butyl (*R*)-2-hydroxy-5-(methoxy(methyl)amino)-5-oxopentanoate (20)

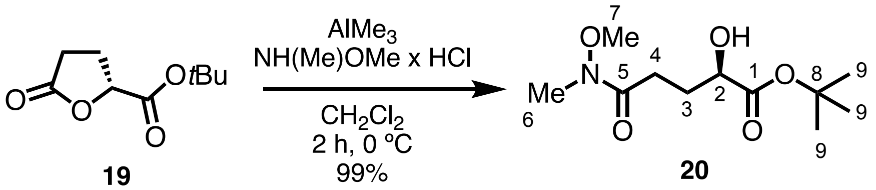

A solution of $AlMe_3$ (1.61 mL, 3.22 mmol, 1.50 eq., 2 M in toluene) was added to a solution of NHMeOMe x HCl (314 mg, 3.22 mmol, 1.50 eq.) in CH_2Cl_2 (36 mL) at 0° C. The mixture was stirred for 20 min at 0 °C and lactone **19** (400 mg, 2.15 mmol, 1.00 eq.) was added portionwise. After stirring for 1 h, the mixture was diluted with $CHCl_3$ (2 mL) and HCl (1 mL, 0.1 M) was added dropwise at 0 °C. The mixture was stirred for 1 h, the organic layers were separated and extracted with CH_2Cl_2 (2x15mL). The combined organic layers were dried over anhydrous $MgSO_4$ and the solvent was removed under reduced pressure to obtain Weinreb amide **20** (520 mg, 2.15 mmol, 99%) as a colorless oil, which was used without further purification.

TLC: R_f = 0.2 (*n*-pentane/EtOAc 2:1). **^{1}H-NMR:** (500 MHz, $CDCl_3$) δ = 4.10 (m, 1H, *H-2*), 3.68 (s, 3H, *H-7*), 3.18 (s, 3H, *H-6*), 3.12 (s, 1H, O*H*), 2.67-2.54 (m, 2H, *H-4*), 2.20-2.12 (m, 1H, *H-3*), 1.93-1.98 (m, 1H, *H-3*), 1.48 (s, 9H, *H-9*) ppm. **^{13}C-NMR:** (500 MHz, $CDCl_3$) δ = 174.2 (*C*-1), 174.0 (*C-5*), 82.6 (*C*-8), 70.1 (*C-2*), 61.3 (*C-7*), 32.3 (*C-6*), 29.2 (*C-3*), 28.0 (*C-9*), 27.5 (*C-*

4) ppm. **HR-MS (ESI⁺):** m/z calc. $C_{11}H_{21}N_1O_5Na$ $[M+Na]^+$: 270.1312, found: 270.1304 m/z. **FT-IR:** film, $\tilde{\nu}$ = 3435 (w), 2977 (w), 2937 (w), 1790 (w), 1727 (m), 1655 (m), 1447 (w), 1419 (w), 1390 (w), 1368 (m), 1279 (w), 1250 (w), 1154 (s), 1097 (w), 995 (m), 951 (w), 911 (w), 845 (m), 792 (w), 747 (w), 606 (w), 499 (w), 470 (w), 437 (w) cm^{-1}. **S.r:** $[\alpha]_D^{20}$=+19.5 (*c* 0.5, $CHCl_3$).

2.9 Synthesis of *tert*-butyl (*R*)-2-((*tert*-butyldimethylsilyl)oxy)-5-(methoxy (methyl)amino)-5-oxopentanoate (21)

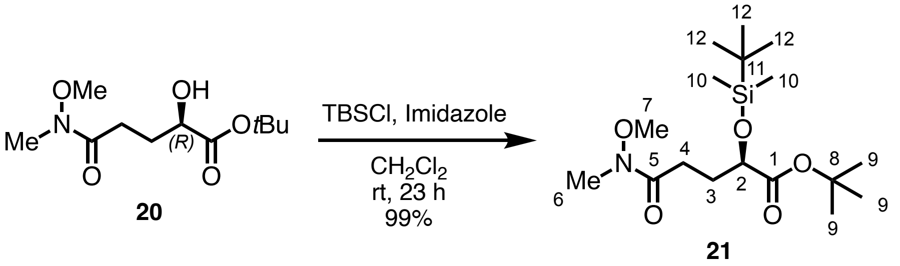

TBSCl (7.83 g, 51.92 mmol, 1.50 eq.) was added to a solution of alcohol **20** (8.56 g, 34.61 mmol, 1.00 eq.) and imidazole (3.53 g, 51.92 mmol, 1.50 eq.) in CH_2Cl_2 (85 mL) at 0 °C and stirred for 23 h. After completion of the reaction water (50 mL) was added and the mixture was extracted with CH_2Cl_2 (3x50 mL). The combined organic layers were washed with brine (50 mL), dried over anhydrous $MgSO_4$ and the solvent was removed under reduced pressure. The crude product was purified by column chromatography (*n*-pentane/EtOAc 2:1) to give TBS-ether **21** (12.30 g, 34.07 mmol, 99%) as a colorless liquid.

TLC: R_f = 0.70 (*n*-pentane/EtOAc 2:1). **¹H-NMR:** (500 MHz, $CDCl_3$) δ = 4.17-4.14 (m, 1H, *H-2*), 3.67 (s, 3H, *H-7*), 3.16 (s, 3H, *H-6*), 2.56-2.51 (m, 2H, *H-4*), 2.11-2.04 (m, 1H, *H-3*), 1.98-1.91 (m, 1H, *H-3*), 1.45 (s, 9H, *H-9*), 0.90 (s, 9H, *H-12*), 0.09 (s, 3H, *H-10*), 0.04 (s, 3H, *H-10*) ppm. **¹³C-NMR:** (500 MHz, $CDCl_3$) δ = 174.2 (*C-5*), 172.1 (*C-1*), 81.1 (*C-8*), 71.7 (*C-2*), 61.3 (*C-7*), 32.3 (*C-6*), 29.9 (*C-3*), 28.1 (C-9), 27.5 (*C-4*), 25.9 (*C-12*), 18.4 (*C-11*), -4.7 (C-*10*), -5.3 (C-*10*) ppm. **HR-MS (ESI⁺):** m/z calc. $C_{17}H_{35}N_1O_5SiH$ $[M+H]^+$: 362.2357, found: 362.2344 m/z. **FT-IR:** film, $\tilde{\nu}$ = 2955 (w), 2932 (m), 2895 (w), 2857 (w), 1745 (m), 1669 (m), 1463 (w), 1414 (w), 1389 (w), 1367 (m), 1301 (w), 1251 (m), 1156 (w), 1128 (s), 1074 (w), 1002 (m), 977 (w), 938 (w), 834 (s), 777 (m), 716 (w), 665 (w), 573 (w), 495 (w), 473 (w), 436 (w) cm^{-1}. **S.r:** $[\alpha]_D^{20}$=+16.5 (*c* 0.5, $CHCl_3$).

2.10 (*S*)-4-benzyl-3-((*R*)-2-methylpent-4-enoyl)oxazolidin-2-one (22b)

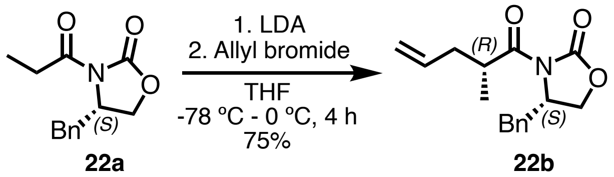

Oxazolidin-2-one **22b** was prepared according to a modified procedure reported by T.W. Moore *et al.*.[3] A solution of (*S*)-Oxazolidinone **22a** (5.00 g, 21.43 mmol, 1.00 eq.) in THF (30 mL) was added into a solution of lithium diisopropyl amide (2 M, 12.86 mL, 25.72 mmol, 1.20 eq.) in THF (100 mL) at −78 °C over 15 minutes. After allowing the solution to stir for 15 minutes at −78 °C, allyl bromide (5.55 mL, 64.30 mmol, 3.00 eq.) was added dropwise over 10 minutes. The reaction was stirred for 30 min at −78 °C followed by additional stirring for 2.5 h at 0 °C. Upon completion sat. NH_4Cl-solution (40 mL) was added and the reaction mixture was extracted with CH_2Cl_2 (3x30 mL). The combined organic layers were washed with brine, dried over anhydrous $MgSO_4$ and the solvent was removed under reduced pressure. The crude product was purified by column chromatography (*n*-pentane/Et_2O 4:1) to give (*R,S*)-Oxazolidinone **22b** (4.35 g, 15.9 mmol, 75%) as a yellow oil. The analytical data are in accordance with the literature.[3]

TLC: R_f = 0.50 (*n*-pentane/EtOAc 4:1). **^{1}H-NMR:** (300 MHz, $CDCl_3$) δ = 7.41-7.21 (m, 5H, 5x$H_{arom.}$), 5.94-5.88 (m, 1H, C*H*), 5.18–5.05 (m, 2H, C*H₂*), 4.76-4.67 (m, 1H, C*H*), 4.26−4.16 (m, 2H, CH_2), 3.89 (*J* = 6.8 Hz, 1H, C*H*), 3.32 (dd, *J* = 12.9, 3.1 Hz, 1 H, CH_2), 2.73 (dd, *J* = 12.9, 10.3 Hz, 1H, CH_2), 2.61-2.50 (m, 1H, CH_2), 2.32-2.21 (dtt, *J* = 14.0, 7.3, 1.3 Hz, 1 H, CH_2), 1.22 (d, *J* = 6.9 Hz, 3H, CH_3) ppm.

2.11 (*R*)-2-methylpent-4-en-1-ol (22c)

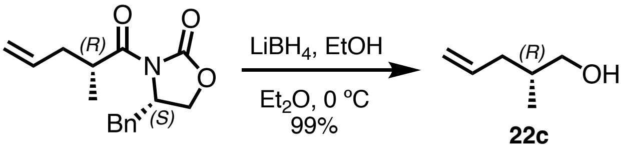

Alcohol **22c** was prepared according to a modified procedure reported by T.W. Moore *et al.*.[3] $LiBH_4$ (0.52 g, 23.87 mmol, 1.50 eq.) and EtOH (1.48 mL, 25.46 mmol, 1.60 eq.) were added to a solution of Oxazolidinone **22b** (4.35 g, 15.91 mmol, 1.00 eq.) in Et_2O (130 mL) at 0 °C. The solution was stirred for 1 h at 0 °C and then stirred for additional 2 h at room temperature. Upon completion the reaction was quenched with 1 M NaOH (40 mL) and extracted with Et_2O (3x20 mL). The combined organic layers were washed with brine, dried over anhydrous $MgSO_4$ and the solvent was removed under reduced pressure. The crude product was purified by column chromatography (*n*-pentane/Et_2O 1:1) to give alcohol **22c** (1.60 g, 15.9 mmol, 99%) as a colorless oil. The analytical data are in accordance with the literature.[3] (Note: the solvent was removed with max. 500 mbar, because of volatility of the product.)

TLC: R_f = 0.50 (*n*-pentane/EtOAc 1:1). **^{1}H-NMR:** (300 MHz, $CDCl_3$) δ = 5.89-5.73 (m, 1H, CH_2=C*H*), 5.10-4.95 (m, 2H, C*H*$_2$=CH), 3.57-3.40 (m, 2H, C*H*$_2$), 2.24-2.11 (m, 1H, C*H*$_2$), 2.02-1.87 (m, 1H, C*H*$_2$), 1.80-1.63 (m, 1H, C*H*), 1.45-1.37 (br s, 1H, O*H*), 0.92 (d, *J* = 6.7 Hz, 3H, C*H*$_3$) ppm.

2.12 (*R*)-5-bromo-4-methylpent-1-ene (22)

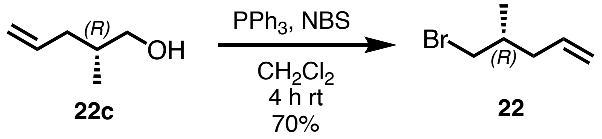

Bromide **22** was prepared according to a modified literature procedure by T. KOCHI *et al.*.[4] NBS (1.17 g, 6.58 mmol, 1.10 eq.) was added to a solution of alcohol **22c** (600 mg, 5.99 mmol, 1.00 eq.) and PPh_3 (1.88 g, 7.19 mmol, 1.20 eq.), in CH_2Cl_2 (38 mL) at 0 °C. The solution was stirred for 19 h at room temperature. Upon completion water (40 mL) was added and the resulting mixture separated and extracted with Et_2O (3x20 mL). The combined organic layers were washed with brine (10 mL), then dried over $MgSO_4$ and the solvent was removed under reduced pressure. The crude product was purified by column chromatography (*n*-pentane) to give bromide **22** (677 mg, 4.15 mmol, 70%) as a colorless oil. The analytical data are in accordance with the literature.[4] (Note: the solvent was removed with max. 500 mbar, because of volatility of the product.)

TLC: R_f = 0.70 (*n*-pentane/Et_2O 20:1). **^{1}H-NMR:** (300 MHz, $CDCl_3$) δ = 5.82-5.67 (m, 1H, $CH_2=CH$), 5.13-5.02 (m, 2H, $CH_2=CH$), 3.43-3.27 (m, 2H, CH_2), 2.56-2.16 (m, 1H, CH_2), 2.10-1.99 (m, 1H, CH_2), 1.93-1.82 (m, 1H, CH), 1.03 (d, J = 6.6 Hz, 3H, CH_3) ppm.

2.13 *tert*-butyl (2*R*,7*R*)-2-((*tert*-butyldimethylsilyl)oxy)-7-methyl-5-oxodec-9-enoate (24)

<u>Preparation of Grignard reagent 23:</u>

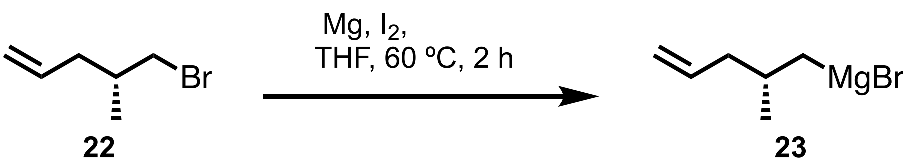

A flame dried round bottom flask equipped with a reflux condenser was charged with mortared magnesium turnings (138 mg, 5.67 mmol, 2.50 eq., activated by heating at 300 °C for 10 min and THF was added (2.5 mL). In a separate flask a solution of the alkyl bromide **22** (370 mg, 2.27 mmol, 1.00 eq.) in THF (2.5 mL) was prepared and drops of this solution were added. Once the Grignard reaction had started, the remaining solution of the alkyl halide **22** was added dropwise, so as to maintain gentle reflux and was stirred at 60 °C for 2-4 h. A small aliquot of the thus obtained Grignard solution **23** was titrated using iodine to determine the concentration (c = 0.45 M) and directly used in the subsequent step.

Grignard Addition:

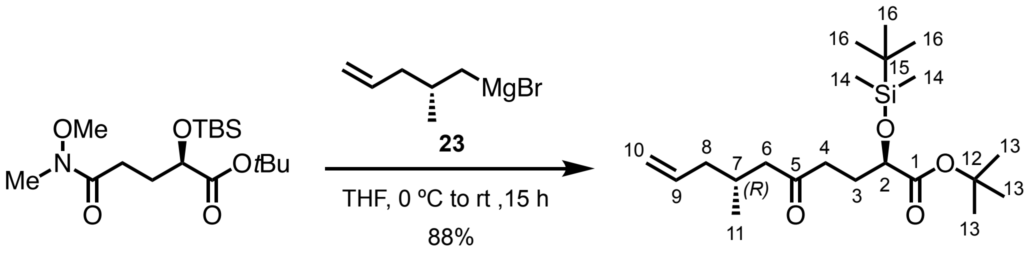

The freshly prepared Grignard solution **23** (2.51 mL, 1.11 mmol, 2.0 eq., c = 0.45 M) was added dropwise into a solution of Weinreb amide **21** (200 mg, 0.55 mmol, 1.0 eq.) in THF (1.0 mL) at 0 °C. The solution was stirred at 0 °C allowing to warm up to rt and stirred for 15 h. Upon completion water (1 mL) was added to the reaction mixture and the aqueous layer was extracted with ethyl acetate (3x10 mL). The combined organic layers were washed with brine (10 mL), dried over $MgSO_4$ and the solvent was removed under reduced pressure. The crude product was purified by column chromatography (*n*-pentane/EtOAc 20:1) to give compound **24** (188 mg, 0.49 mmol, 88%) as a colorless oil.

TLC: R_f = 0.80 (*n*-pentane/EtOAc 20:1). **^{1}H-NMR:** (500 MHz, $CDCl_3$) δ = 5.78-5.69 (m, 1H, *H-9*), 5.01 (s, 1H, *H-10*), 4.99-4.79 (m, 1H, *H-10*), 4.12-4.09 (m, 1H, *H-2*), 2.55- 2.39 (m, 3H, *H-6, H-4*), 2.22-2.18 (m, 1H, *H-6*), 2.13-2.07 (m, 1H, *H-7*), 2.04-1.94 (m, 3H, *H-8, H-3*), 1.94-1.86 (m, 1H, *H-3*), 1.45 (s, 9H, *H-13*), 0.90 (s, 9H, *H-16*), 0.89 (d, *J* = 6.6 Hz, 3H, *H-11*), 0.08 (s, 3H, *H-14*), 0.35 (s, 3H, *H-14*) ppm. **^{13}C-NMR:** (500 MHz, $CDCl_3$) δ (ppm) = 210.2 (*C-5*), 172.7 (*C-1*), 136.8 (*C-9*), 116.6 (*C-10*), 81.2 (*C-12*), 71.5 (*C-2*), 49.5 (*C-6*), 41.3 (*C-8*), 38.5 (*C*-4), 29.8 (*C-7*), 29.1 (*C-3*), 28.2 (*C-13*), 25.9 (*C-16*), 19.92 (*C-11*), 18.4 (*C-15*), -4.7 (*C-14*), -5.2 (*C-14*) ppm. **HR-MS (ESI$^+$):** m/z calc. $C_{21}H_{40}O_4Si_1Na_1$ $[M+Na]^+$: 407.2588, found: 407.2583 m/z. **FT-IR:** film, $\tilde{\nu}$ = 3077 (w), 2955 (w), 2929 (m), 2857 (w), 1746 (w), 1717 (m), 1641 (w), 1461 (w), 1411 (w), 1391 (w), 1367 (m), 1299 (w), 1252 (m), 1130 (s), 1049 (w), 1005 (w), 972 (w), 939 (w), 912 (w), 835 (s), 811 (w), 778 (m), 719 (w), 666 (w), 626 (w), 575 (w), 496 (w), 473 (w), 408 (w) cm^{-1}. **S.r:** $[\alpha]_D^{20}$=+31.3 (*c* 0.5, $CHCl_3$).

2.14 *tert*-butyl (2*S*,7*R*,*E*)-2-((*tert*-butyldimethylsilyl)oxy)-10-(6-((*S*)-4-((*tert*-butyldimethylsilyl)oxy)butan-2-yl)pyridin-2-yl)-7-methyl-5-oxodec-9-enoate (25)

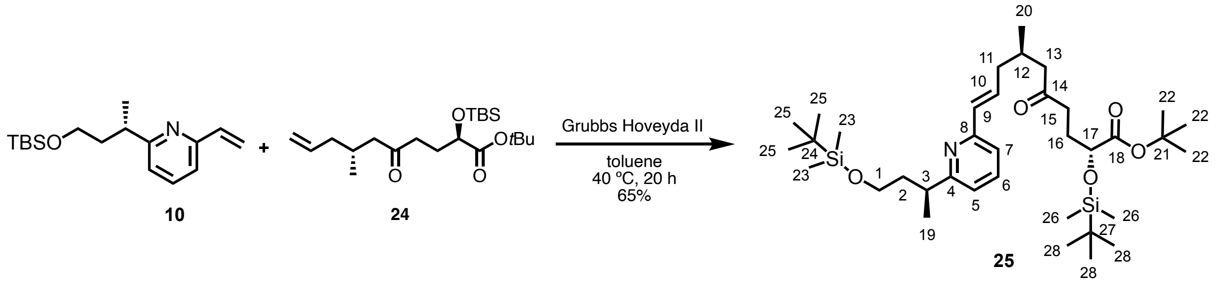

Grubbs II cat. (21.5 mg, 0.034 mmol, 0.10 eq.) was added into a solution of Alkene **24** (238 mg, 0.61 mmol, 1.80 eq.) and vinyl pyridine **10** (100 mg, 0.618 mmol, 1.00 eq.) in toluene (30 mL) and stirred for 20 h at 40 °C. Grubbs II cat. (12.0 mg, 0.017 mmol, 0.05 eq.) was added and stirred for 20 h at 40 °C. The reaction mixture was filtered over Celite, reduced in volume to 0.5 mL and purified with column chromatography (*n*-pentane/EtOAc 30:1 to 10:1) to give compound **25** (145 mg, 0.223 mmol, 65%) as a pale yellow oil.

TLC: R_f = 0.48 (*n*-pentane/EtOAc 20:1). **^{1}H-NMR:** (500 MHz, $CDCl_3$) δ = 7.49 (t, 1H, *J* = 7.7 Hz, *H-6*), 7.06 (d, 1H, *J* = 7.7 Hz, *H-7)*, 6.94 (d, 1H, *J* = 7.7 Hz, *H-5)*, 6.65-6.59 (m, 1H, *H-10*), 6.45 (d, 1H, *J* = 15.7 Hz, *H-9)*, 4.12-4.09 (m, 1H, *H-17*), 3.59-3.50 (m, 2H, *H-1*), 3.02-2.97 (m, 1H, *H-3*), 2.52-2.46 (m, 3H, *H-11*, *H-13*), 2.30-2.13 (m, 4H, *H-12*, *H-13, H-15*), 2.04-1.97 (m, 2H, *H-2*, *H-16*), 1.92-1.85 (m, 3H, *H-16*), 1.83-1.77 (m, 1H, *H-2*), 1.44 (s, 9H, *H-22),* 1.27 (d, 3H, *J* = 7.0 Hz, *H-19)*, 0.95 (d, 3H, *J* = 6.0 Hz, *H-20)*, 0.89 (s, 9H, *H-25)*, 0.86 (s, 9H, *H-28), 0.*07 (s, 3H, *H-23)*, 0.02 (s, 3H, *H-23*), 0.07 (s, 6H, *H-26)* ppm. **^{13}C-NMR:** (126 MHz, $CDCl_3$) δ = 210.1 (*C14*), 172.7 (*C-18*), 165.8 (*C-4*), 155.2 (*C-8*), 136.6 (*C-6*), 132.8 (*C-10*), 132.6 (*C-9*), 119.7 (*C-7*), 118.2 (*C-5*), 81.2 (*C-21*), 71.5 (*C-17*), 61.6 (*C-1*), 49.8 (*C-13*), 40.3 (*C-15*), 39.8 (*C-2*), 38.5 (*C-11, C-3*), 29.4 (*C-12*), 28.9 (*C-16*), 28.2 (*C-22*), 26.1 (*C-25*), 25.9 (*C-28*), 21.9 (*C-19*), 20.1 (*C-20*), 18.4 (*C-27, C-21*), -4.7 (*C-26*), -5.2 (*C-23*) ppm. **HR-MS (ESI$^+$):** m/z calc. $C_{36}H_{65}NO_5Si_2Na$ $[M+Na]^+$: 670.4293, found: 670.4270 m/z. **FT-IR:** film, $\tilde{\nu}$ =2955 (w), 2930 (m), 2888 (w), 2857 (w), 1746 (w), 1716 (m), 1654 (w), 1571 (w), 1461 (w), 1409 (w), 1390 (w), 1367 (w), 1299 (w), 1253 (m), 1129 (m), 1006 (w), 973 (w), 939 (w), 899 (w), 835 (s), 777 (m), 745 (w), 664 (w), 476 (w) cm^{-1}. **S.r:** $[\alpha]_D^{20}$=+33.5 (*c* 0.5, $CHCl_3$).

2.15 *tert*-butyl (2*S*,7*R*,9*S*,10*S*)-2-((*tert*-butyldimethylsilyl)oxy)-10-(6-((*S*)-4-((*tert*-butyldimethyl-silyl)oxy)butan-2-yl)pyridin-2-yl)-9,10-dihydroxy-7-methyl-5-oxodecanoate (27)

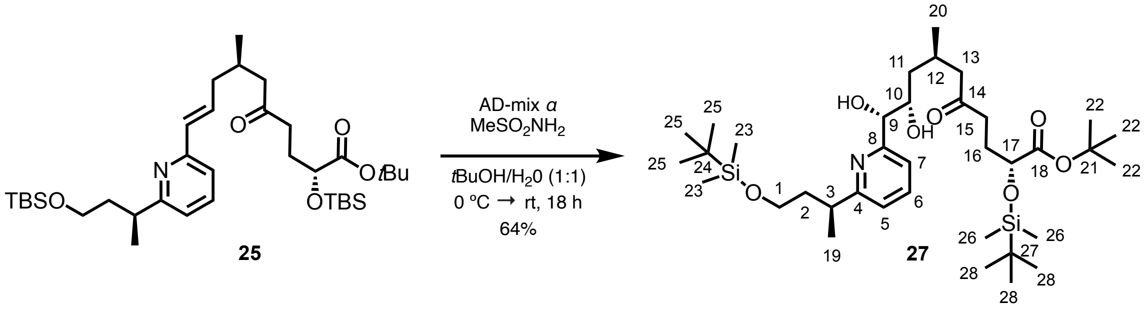

Diol **27** was prepared according to a modified procedure by Sharpless *et al.*.[5]

Olefin **25** (50.0 mg, 0.077 mmol, 1.00 eq.) was dissolved in *t*BuOH (1.00 mL) and degassed H_2O (1.0 mL) at 0 °C. A self-prepared AD-mix α, consisted of $K_2[OsO_2(OH)_4]$ (5.70 mg, 0.015 mmol, 0.20 eq.), $(DHQ)_2$Phal (24.0 mg, 0.031 mmol, 0.40 eq.), K_2CO_3 (32.0 mg, 0.231 mmol, 3.00 eq.), $K_3[Fe(CN)_6]$ (76.20 mg, 0.231 mmol, 3.00 eq.) and Methanesulfonamine (8.1 mg, 0.085 mmol, 1.10 eq.) was added and stirred for 18 h at 0 °C allowing to warm up to room temperature. Upon completion sat. Na_2SO_3 (10 mL) was added. The aqueous layers were extracted with EtOAc (3x10 mL). The combined organic layers were washed with brine, dried over anhydrous $MgSO_4$ and the solvent was removed under reduced pressure. The crude product was purified by column chromatography (*n*-pentane/EtOAc 3:1) to give (*S*,*S*)-alcohol **27** (33.5 mg, 0.049 mmol, 64%, dr > 25:1) as a colorless oil.

TLC: R_f = 0.31 (*n*-pentane/EtOAc 4:1). **^{1}H-NMR:** 500 MHz, $CDCl_3$; *δ* = 7.61 (t, 1H, *J* = 7.7 Hz, *H-6*), 7.14 (d, 1H, *J* = 7.7 Hz, *H-7)*, 7.08 (d, 1H, *J* = 7.8 Hz, *H-5)*, 4.53 (d, 1H, *J* = 3.3 Hz, *H-9), 4*.11-4.09 (m, 1H, *H-17*), 3.88-3.84 (m, 1H, *H-10),* 3.58-3.53 (m, 1H, *H-1),* 3.49-3.44 (m, 1H, *H-1),* 3.09-3.04 (q, 1H, *J* = 7.1 Hz, *H-3*), 2.55-2.48 (m, 3H, *H-15, H-13*), 2.30-2.22 (m, 2H, *H-13, H-11*), 2.05-1.84 (m, 3H, *H-2*, *H-16*), 1.81-1.75 (m, 1H, *H-2*), 1.56-1.50 (m, 2H, *H-12, H-11*), 1.45 (s, 9H, *H-22),* 1.26 (d, 3H, *J* = 7.1 Hz, *H-19)*, 0.95 (d, 3H, *J* = 6.4 Hz, *H-20)*, 0.89 (s, 9H, *H-25)*, 0.86 (s, 9H, *H-28), 0*.07 (s, 3H, *H-23)*, 0.02 (s, 3H, *H-23*), -0.03 (s, 3H, *H-26),*-0.02 (s, 3H, *H-26)* ppm. (Note: The OH protons of the alcohol groups are not observed in the ^{1}H-NMR spectrum due to rapid proton exchange and were therefore not included in the integration.) **^{13}C-NMR:** 126 MHz, $CDCl_3$; *δ* (ppm) = 210.7 (*C-14*), 172.7 (*C-18*), 164.8 (*C-4*), 158.9 (*C-8*), 137.5 (*C-6*), 121.4 (*C-5*), 119.2 (*C-7*), 81.2 (*C-21*), 74.4 (*C-9*), 72.5 (*C-10*)*,* 71.5 (*C-17*), 61.1 (*C-1*), 49.8 (*C-13*), 40.3 (*C-12*), 39.7 (*C-2*), 38.4 (*C-15*), 37.9 (*C-3*), 29.9 (*C-21*), 29.0 (*C-16*), 28.1 (*C-22*), 26.1 (*C-25*), 26.0 (*C-11*), 25.9 (*C-28*), 21.0 (*C-20*), 20.9 (*C-19*), 18.4 (*C-27,C-24*), -4.7 (*C-26*), -5.2 (*C-23*), -5.3 (*C-26*) ppm. **HR-MS (ESI$^+$):** m/z calc. $C_{36}H_{67}NO_7Si_2Na$ $[M+Na]^+$: 704.4348, found: 704.4343 m/z. **FT-IR:** film, $\tilde{\nu}$ = 3406 (w), 2956 (w),

2932 (s), 2891 (w), 2858 (w), 2060 (w), 1746 (w), 1717 (m), 1593 (w), 1576 (w), 1463 (w), 1408 (w), 1390 (w), 1367 (w), 1298 (w), 1254 (m), 1154 (w), 1130 (m), 1105 (w), 1006 (w), 973 (w), 940 (w), 837 (s), 812 (w), 778 (m), 726 (w), 699 (w), 665 (w), 639 (w), 597 (w), 502 (w), 458 (w), 425 (w) cm^{-1}. **S.r**$[\alpha]_D^{20}$=+30.5 (*c* 0.5, $CHCl_3$).

2.16 *tert*-butyl (2*S*,7*R*)-2-((*tert*-butyldimethylsilyl)oxy)-8-((2*S*,3*S*)-3-(6-((*S*)-4 ((*tert*butyldimethyl-silyl)oxy)butan-2-yl)pyridin-2-yl)oxiran-2-yl)-7-methyl-5-oxooctanoate (26)

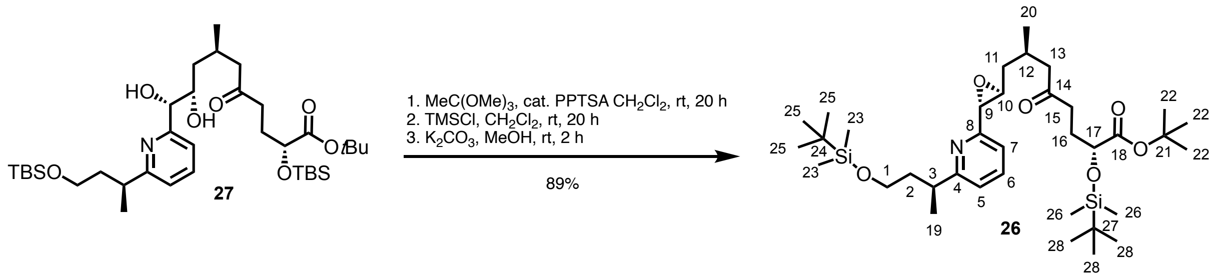

Stepwise mechanism:

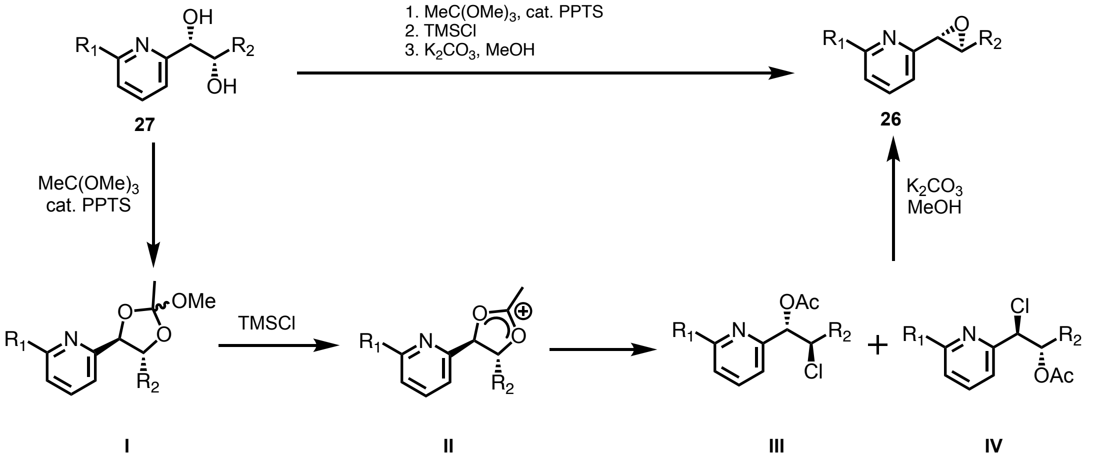

Epoxide **26** was prepared according to an adapted procedure by Sharpless *et al.*.[6]

Diol **27** (58.0 mg, 0.085 mmol, 1.00 eq.) was dissolved in CH_2Cl_2 (1.0 mL). $MeC(OMe)_3$ (33.0 µL, 0.255 mmol, 3.00 eq.) and PPTA (2.10 mg, 0.01 mmol, 0.10 eq.) were added. The reaction mixture was stirred for 2 h at room temperature. After consumption of the starting material the solvent and volatiles were removed under vacuo and redissolved in CH_2Cl_2 (1.0 mL). TMSCl (32.0 µL, 0.255 mmol, 3.00 eq.) was added and stirred for 1 h. The solvent and volatiles were removed under vacuo and redissolved in MeOH (1.0 mL). K_2CO_3 (59.0 mg, 0.425 mmol, 5.00 eq.) was added and stirred for 2 h. The reaction was quenched with a sat. NH_4Cl-sol. The aqueous layer was extracted with CH_2Cl_2 (3x10 mL). The combined organic phases were washed with brine, dried over anhydrous $MgSO_4$ and the solvent was removed under reduced pressure. The crude product was purified by column chromatography (*n*-pentane/EtOAc 8:1) to give (*S*,*S*)-epoxide **26** (50.0 mg, 0.075 mmol, 89%) as a colorless oil.

TLC: R_f = 0.83 (*n*-pentane/EtOAc 5:1). **^{1}H-NMR:** (500 MHz, $CDCl_3$) δ = 7.54 (t, 1H, *J* = 7.7 Hz, *H-6*), 7.03 (d, 1H, *J* = 7.7 Hz, *H-5)*, 6.98 (d, 1H, *J* = 7.8 Hz, *H-7)*, *4*.12-4.09 (m, 1H, *H-17*), 3.70 (d, 1H, *J* = 2.1 Hz, *H-9),* 3.58-3.48 (m, 2H, *H-1),* 3.08-2.99 (m, 2H, *H-10, H-3),* 2.57-2.47 (m, 3H, *H-13, H-15*), 2.41-2.31 (m, 2H, *H-13, H-11*), 2.07-1.95 (m, 2H, *H-2*, *H-16*), 1.89-1.76 (m, 3H, *H-2*, *H-16*, *H-11*), 1.55-1.49 (m, 1H, *H-12*), 1.45 (s, 9H, *H-22),* 1.27 (d, 3H, *J* = 6.8 Hz, *H-19)*, 0.95 (d, 3H, *J* = 6.3 Hz, *H-20)*, 0.89 (s, 9H, *H-25)*, 0.86 (s, 9H, *H-28), 0.*08 (s, 3H, *H-26*), -0.01 (s, 3H, *H-26*), -0.02 (s, 6H, *H-23)* ppm. (Note: The OH protons of the alcohol groups are not observed in the ^{1}H-NMR spectrum due to rapid proton exchange and were therefore not included in the integration.) **^{13}C-NMR:** (126 MHz, $CDCl_3$) δ (ppm) = 209.6 (*C-14*), 172.6 (*C-18*), 166.8 (*C-4*), 156.8 (*C-8*), 136.9 (*C-6*), 121.0 (*C-5*), 116.6 (*C-7*), 81.2 (*C-21*), 71.4 (*C-17*), 61.4 (*C-1*), 60.8 (*C-10*)*, 58*.8 (*C-9*), 49.6 (*C-13*), 39.5 (*C-2*), 39.0 (*C-12*), 38.5 (*C-15*), 38.3 (*C-3*), 28.9 (*C-16*), 28.1 (*C-22*), 27.7 (*C-11*), 26.0 (*C-28*), 25.8 (*C-25*), 21.2 (*C-19*), 20.2 (*C-20*), 18.4 (*C-27,C-24*), -4.7 (*C-26*), -5.2 (*C-23*), -5.3 (*C-23*) ppm. **HR-MS (ESI$^+$):** m/z calc. $C_{36}H_{65}NO_6Si_2Na$ $[M+Na]^+$: 686.4243, found: 686.4240 m/z. **FT-IR:** film, $\tilde{\nu}$ = 2955 (w), 2929 (s), 2857 (w), 1746 (w), 1718 (m), 1592 (w), 1576 (w), 1463 (m), 1411 (w), 1367 (w), 1300 (w), 1253 (m), 1131 (m), 1106 (w), 1007 (w), 974 (w), 940 (w), 895 (w), 836 (s), 812 (w), 778 (m), 753 (w), 664 (w), 626 (w), 498 (w), 449 (w) cm^{-1}. **S.r:** $[\alpha]_D^{20}$=+26.6 (*c* 0.5, $CHCl_3$).

2.17 *tert*-butyl (2*S*,7*R*)-2-hydroxy-8-((2*S*,3*S*)-3-(6-((*S*)-4-hydroxybutan-2-yl)pyridin-2-yl)oxiran-2-yl)-7-methyl-5-oxooctanoate (28)

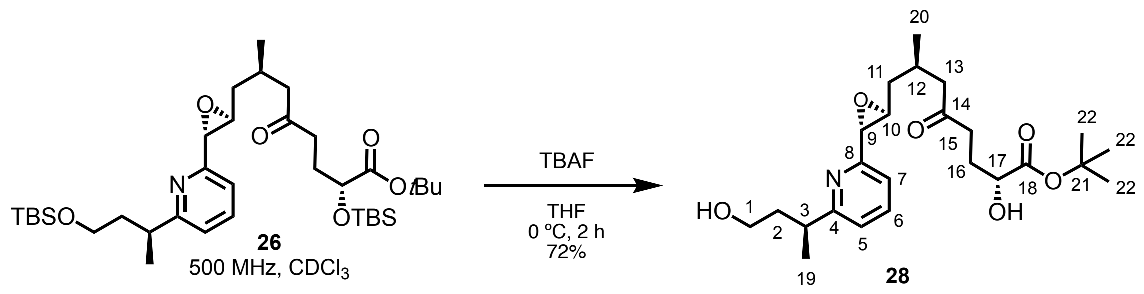

TBAF (1 mol/L in THF, 0.42 mL, 0.422 mmol, 2.50 eq.) was added to a solution of di-TBS-Ether **26** (112.0 mg, 0.169 mmol, 1.00 eq.) in THF (3.0 mL) at 0°C and the mixture was stirred for 3 h. After completion of the reaction water was added and the aqueous layer was extracted with EtOAc (3x15 mL). The combined organic layers were dried over anhydrous $MgSO_4$ and the solvent was removed under reduced pressure. The crude product was purified by flash column chromatography (*n*-pentane/EtOAc 1:2) to give di-alcohol **28** (53.0 mg, 0.122 mmol, 72%) as a colorless oil.

TLC: R_f = 0.45 (*n*-pentane/EtOAc 1:2). **^{1}H-NMR:** (500 MHz, $CDCl_3$) δ = 7.61 (t, 1H, *J* = 7.8 Hz, *H-6*), 7.11 (d, 1H, *J* = 7.8 Hz, *H-5)*, 7.02 (d, 1H, *J* = 7.8 Hz, *H-7)*, *4.*02 (dd, 1H, *J* = 7.8, 4.0 Hz, *H-17*), 3.68 (d, 1H, *J* = 1.8 Hz, *H-9)*, 3.65-3.60 (m, 1H, *H-1)*, 3.59-3.53 (m, 1H, *H-1)*, 3.04-3.02 (m, 1H, *H-10)*, 2.64-2.50 (m, 3H, *H-13, H-15*), 2.40-2.29 (m, 2H, *H-13, H-12*), 2.13-2.03 (m, 1H, *H-16*), 2.05-2.00 (m, 1H, *H-11*), 1.92-1.87 (m, 4H, *H-2, H-3*, *H-11*, *H-16*), 1.62-1.55 (m, 1H, *H-2*), 1.47 (s, 9H, *H-22)*, 1.32 (d, 3H, *J* = 6.8 Hz, *H-19)*, 0.95 (d, 3H, *J* = 6.4 Hz, *H-20)* ppm. (Note: The OH protons of the alcohol groups are not observed in the ^{1}H-NMR spectrum due to rapid proton exchange and were therefore not included in the integration.) **^{13}C-NMR:** 126 MHz, $CDCl_3$; δ (ppm) = 209.9 (*C-14*), 174.2 (*C-18*), 165.4 (*C-4*), 156.4 (*C-8*), 137.8 (*C-6*), 120.7 (*C-5*), 117.1 (*C-7*), 82.7 (*C-21*), 69.7 (*C-17*), 61.0 (*C-10*), 60.4 (*C-1*), *58.*7 (*C-9*), 49.6 (*C-13*), 38.9 (*C-3, C-11*), 38.8 (*C-2*), 38.6 (*C-15*), 28.2 (*C-16*), 28.1 (*C-22*), 27.6 (*C-12*), 20.5 (*C-19*), 20.4 (*C-20*) ppm. **HR-MS (ESI$^+$):** m/z calc. $C_{24}H_{37}NO_6Na$ $[M+Na]^+$: 458.2513, found: 458.2505 m/z. **FT-IR:** film, $\tilde{\nu}$ =3419 (w), 2962 (w), 2929 (m), 2875 (w), 1713 (s), 1593 (w), 1575 (m), 1460 (m), 1412 (w), 1369 (m), 1279 (w), 1253 (w), 1156 (s), 1116 (w), 1048 (w), 996 (w), 958 (w), 890 (w), 847 (w), 813 (m), 753 (w), 669 (w), 628 (w), 473 (w) cm^{-1}. **S.r**: $[\alpha]_D^{20}$=+18.9 (*c* 0.5, $CHCl_3$).

2.18 *tert*-butyl (2*S*,5*R*,7*S*,9*R*)-7-((*S*)-hydroxy(6-((*S*)-4-hydroxybutan-2-yl)pyridin-2-yl)methyl)-9-methyl-1,6-dioxaspiro[4.5]decane-2-carboxylate (29)

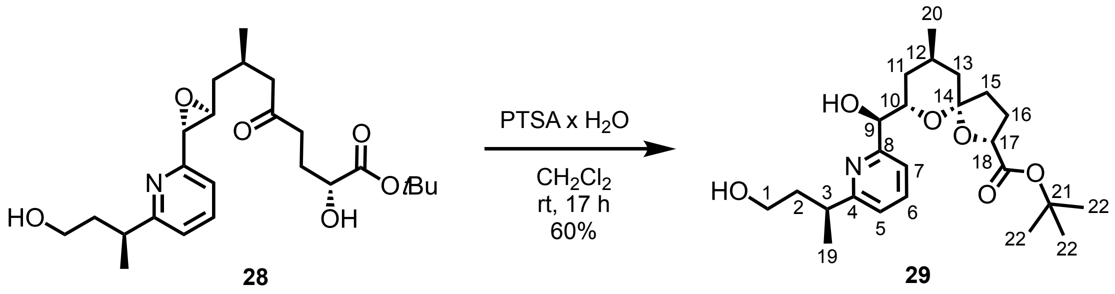

PTSA x H_2O (4.5 mg, 0.023 mmol, 1.00 eq) was added to a solution of epoxy-ketone **28** (10.0 mg, 0.023 mmol, 1.00 eq) in CH_2Cl_2 (1.0 mL) and the mixture was stirred for 17 h. After completion of the reaction sat.-$NaHCO_3$ sol. was added and the aqueous layer was extracted with EtOAc (3x10 mL). The organic layers were combined and washed with sat.-$NaHCO_3$, brine, dried over anhydrous $MgSO_4$ and the solvent was removed under reduced pressure. The crude product was purified by flash column chromatography (*n*-pentane/EtOAc 1:2) to give spiroketal **29** (6.0 mg, 0.0138 mmol, 60%) as a colorless oil.

TLC: R_f = 0.50 (*n*-pentane/EtOAc 1:2). **^{1}H-NMR:** (500 MHz, $CDCl_3$) δ = 7.62 (t, 1H, *J* = 8.1 Hz, *H-6*), 7.36 (d, 1H, *J* = 7.8 Hz, *H-7)*, 7.06 (d, 1H, *J* = 7.8 Hz, *H-5)*, 4.77 (d, 1H, *J* = 3.1 Hz, *H-9), 4.*60 (dd, 1H, *J* = 12.2, 3.2 Hz, *H-10*), 4.51 (t, 1H, *J* = 8.0 Hz, *H-17*), 3.63-3.59 (m, 1H, *H-1),* 3.54-3.48 (m, 1H, *H-1),* 3.16-3.11 (m, 1H, *H-3),* 2.27-2.19 (m, 2H ,*H-16*), *2*.14-2.07 (m, 1H, *H-15),* 2.04-2.00 (m, 1H, *H-2, H-12),* 1.95-1.85 (m, 3H, *H-13, H-2), 1*.71-1.63 (m, 2H, *H-15*, *H-11),* 1.51-1.48 (m, 1H, *H-2*), 1.46 (s, 9H, *H-22)*, 1.32 (d, 3H, *J* = 7.0 Hz, *H-19)*, 1.12 (d, 3H, *J* = 6.6 Hz, *H-20).* 0.93-0.86 (m, 1H, *H-11).* (Note: The OH protons of the alcohol groups are not observed in the ^{1}H-NMR spectrum due to rapid proton exchange and were therefore not included in the integration.) **^{13}C-NMR:** (126 MHz, $CDCl_3$) δ = 172.8 (*C-18*), 163.6 (*C-4*), 158.6 (*C-8*), 137.2 (*C-6*), 120.2 (*C-5*), 119.6 (*C-7*), 108.4 (*C-14*), 81.0 (*C-21*), 78.0 (*C-17*), 75.1 (*C-9*), 69.8 (*C-10*)*, 60.*9 (*C-1*), 39.6 (*C-2*), 39.1 (*C-15*), 38.3 (*C-3*), 37.6 (*C-13*), 29.0 (*C-11*), 28.3 (*C-22*), 27.5 (*C-16*), 25.2 (*C-12*), 22.8 (*C-20*), 20.3 (*C-19*) ppm. **HR-MS (ESI$^+$):** m/z calc. $C_{24}H_{37}NO_6H$ $[M+H]^+$: 436.2694, found: 436.2689 m/z. **FT-IR:** film, $\tilde{\nu}$ = 3463 (w), 2974 (m), 2904 (w), 1773 (m), 1620 (w), 1605 (w), 1486 (m), 1438 (w), 1415 (w), 1392 (m), 1337 (w), 1248 (w), 1179 (m), 1156 (w), 1095 (w), 1072 (s), 1019 (w), 986 (w), 909 (w), 861 (w), 827 (w), 771 (w), 744 (w), 599 (w), 521 (w) cm^{-1}. **S.r:** $[\alpha]_D^{20}$=+23.55 (*c* 0.3, $CHCl_3$).

2.19 *tert*-butyl (2*R*,5*S*,7*R*,9*R*)-7-((*S*)-hydroxy(6-((*S*)-4-hydroxybutan-2-yl)pyridin-2-yl)methyl)-9-methyl-1,6-dioxaspiro[4.5]decane-2-carboxylate (30)

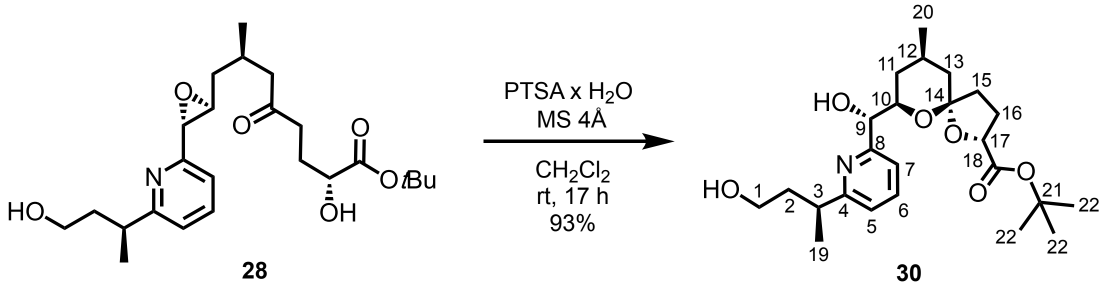

PTSA x H_2O (7.5 mg, 0.038 mmol, 1.20 eq.) was added to a solution of epoxy-ketone **28** (14.0 mg, 0.032 mmol, 1.00 eq.) and molecular sieve 4Å in CH_2Cl_2 (1.0 mL) and the mixture was stirred for 17 h. After completion of the reaction sat.-$NaHCO_3$ sol. was added and the aqueous layer was extracted with EtOAc (3x10 mL). The organic layers were combined and washed with sat.-$NaHCO_3$, brine, dried over anhydrous $MgSO_4$ and the solvent was removed under reduced pressure. The crude product was purified by flash column chromatography (*n*-pentane/EtOAc 1:2) to give spiroketal **30** (13.0 mg, 0.030 mmol, 93%) as a colorless oil.

TLC: R_f = 0.55 (*n*-pentane/EtOAc 1:2). **^{1}H-NMR:** (500 MHz, $CDCl_3$) δ = 7.59 (t, 1H, *J* = 7.7 Hz, *H-6*), 7.15 (d, 1H, *J* = 7.7 Hz, *H-7)*, 7.07 (d, 1H, *J* = 7.8 Hz, *H-5)*, 4.60 (d, 1H, *J* = 5.7 Hz, *H-9), 4.*13 (dd, 1H, *J* = 8.92, 4.4 Hz, 1H, *H-17*), 3.86 (ddd, 1H, *J* = 11.5, 8.0, 2.0 Hz 1H, *H-10*), 3.63-3.57 (m, 1H, *H-1),* 3.55-3.49 (m, 1H, *H-1),* 3.15-3.07 (m, 1H, *H-3),* 2.30-2.21 (m, 1H , *H-16*), 2.04-1.94 (m, 2H, *H-2, H-12),* 1.89-1.82 (m, 3H, *H-13, H-15, H-16), 1.*77-1.71 (m, 1H, *H-15), 1.*52-1.47 (m, 1H, *H-11),* 1.44 (s, 9H, *H-22)*, 1.31 (d, 3H, *J* = 6.8 Hz, *H-19)*, 1.31 (q, 1H, *J* = 12.9 Hz, *H-13)*, 1.05 (q, 1H, *J* = 12.5 Hz, *H-11)*, 0.88 (d, 3H, *J* = 6.6 Hz, *H-20).* (Note: The OH protons of the alcohol groups are not observed in the ^{1}H-NMR spectrum due to rapid proton exchange and were therefore not included in the integration.) **^{13}C-NMR:** (126 MHz, $CDCl_3$) δ = 172.3 (*C-18*), 164.2 (*C-4*), 158.6 (*C-8*), 136.8 (*C-6*), 120.6 (*C-5*), 119.5 (*C-7*), 108.2 (*C-14*), 81.3 (*C-21*), 76.6 (*C-17*), 74.8 (*C-9*), 74.4 (*C-10*), *60.*9 (*C-1*), 41.2 (*C-13*), 39.4 (*C-2*), 38.3 (*C-3*), 36.7 (*C-15*), 33.5 (*C-11*), 28.7 (*C-16*), 28.1 (*C-22*), 26.3 (*C-12*), 22.3 (*C-20*), 20.9 (*C-19*) ppm. **HR-MS (ESI$^+$):** m/z calc. $C_{24}H_{37}NO_6Na$ $[M+Na]^+$: 458.2513, found: 458.2507 m/z. **FT-IR:** film, $\tilde{\nu}$ = 3419 (w), 2952 (m), 2872 (w), 1740 (m), 1593 (w), 1576 (w), 1457 (m), 1369 (m), 1331 (w), 1295 (w), 1211 (w), 1156 (m), 1132 (w), 1059 (s), 1003 (w), 962 (w), 909 (w), 877 (w), 849 (m), 815 (w), 757 (w), 731 (m), 645 (w), 573 (w), 470 (w) cm^{-1}. **S.r:** $[\alpha]_D^{20}$=+12.5 (*c* 0.5, $CHCl_3$).

2.20 (2*R*,5*S*,7*R*,9*R*)-7-((*S*)-hydroxy((*S*)-1-methyl-2,3-dihydro-1*H*-indolizin-4-ium-5-yl)methyl)-9-methyl-1,6-dioxaspiro[4.5]decane-2-carboxylate, Voratin C (3)

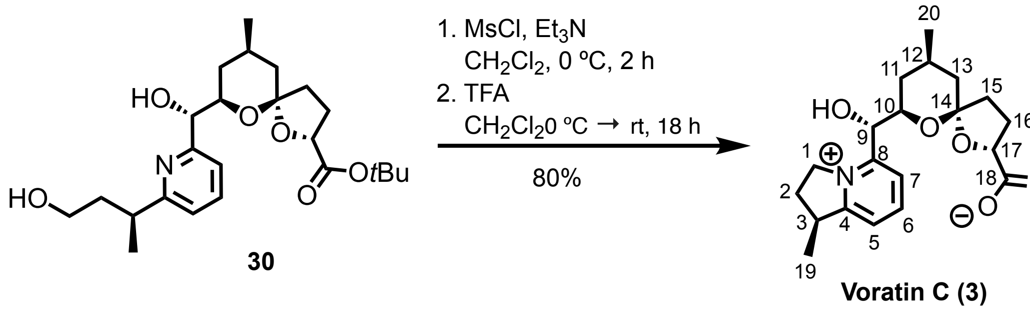

Et_3N (3.0 µL, 0.025 mmol, 1.2 eq.) and MsCl (2.0 µL, 0.025 mmol, 1.10 eq.) were added to a solution of spiroketal **30** (9.0 mg, 0.021 mmol, 1.00 eq.) in CH_2Cl_2 (0.5 mL) at 0 °C and the mixture was stirred for 2 h allowing to warm up to rt. After completion of the reaction volatiles were removed under reduced pressure. The residue was redissolved in CH_2Cl_2 (1.0 mL). TFA (80 µL, 0.99 mmol, 50.00 eq.) was added dropwise into the solution at 0 °C and stirred for 18 h allowing to warm up to rt. The volatiles were removed under reduced pressure. The crude product was purified by semi-preparative RP-HPLC ACE SuperC$_{18}$-column (150 × 10 mm, 5 µm, 90Å), MeCN + 0.085% TFA/H_2O + 0.01% TFA, 10% to 70% in 20 min) to obtain voratin C as TFA salt (**31**) as a colorless resin. Voratin C x TFA (**31**) was converted to its zwitterionic form by stepwise ion exchange using the following procedure:

1. Dissolve and freeze dry through lyophilization in aq. HCl (10 mL 50 mM), 2x(5 mL, 20 mM).
2. Dissolve and freeze dry through lyophilization in aq. ammonium formate (10 mL 20 mM), 2x(5 mL, 10 mM).
3. Dissolve in water (10 mL, 2x5 mL) and freeze dry through lyophilization.

After ion exchange voratin C (**3**) was obtained (6.0 mg, 0.016 mmol, 80%) as a white powder.

RP-TLC: R_f = 0.40 (MeCN/H_2O 3:7). **^{1}H-NMR:** (500 MHz, $CDCl_3$) δ = 8.52 (t, 1H, *J* = 8.1 Hz, *H-6*), 8.04 (d, 1H, J = 8.1 Hz, *H-7*), 7.95 (d, 1H, *J* = 8.1 Hz, *H-5*), 5.05 (ddd, 1H, *J* = 12.5, 9.2, 3.5 Hz, *H-1a*), 4.70 (d, 1H, J = 8.8 Hz, *H-9*), 4.70 (ddd, 1H, J = 12.5, 8.7, 8.1 Hz, *H-1b*), 3.82 (ddd, 1H, *J* = 11.0, 8.8, 1.8 Hz, *H-10*), 3.78 (tq, 1H, *J* = 8.8, 7.1 Hz, *H-3*), 3.51 (dd, 1H, *J* = 9.3, 5.3 Hz, *H-17*), 2.74 (dtd, 1H, *J* = 12.9, 8.5, 3.8 Hz, *H-2a*), 2.16 (br d, 1H, *J* = 12.8 Hz, *H-11a*), 2.09 (m, 1H, H-12), 2.02 (dq, 1H, *J* = 12.6, 8.8 Hz, *H-2b*), 1.90 (dd, 1H, *J* = 13.0, 2.8 Hz, *H-13a*), 1.70–1.57 (m, 4H, *H-15, H-16*), 1.54 (d, 3H, *J* = 7.1 Hz, *H-19*), 1.34 (t, 1H, *J* = 12.8 Hz, *H-13b*), 1.07 (dd, 1H, *J* = 12.1, 12.0 Hz, *H-11b*), 0.98 (d, 3H, *J* = 6.4 Hz, *H-20*). (Note: The OH protons of the alcohol groups are not observed in the ^{1}H-NMR spectrum due to rapid proton exchange and were therefore not included in the integration.) **^{13}C-NMR:** (126 MHz, $CDCl_3$) *δ* = 178.3 (br, *C-18*), 162.9 (*C-4*), 159.9 (*C-8*), 147.0 (*C-6*), 124.7 (*C-7*), 123.2 (*C-5*), 108.8 (*C-14*), 79.0 (*C-17*), 76.6 (*C-10*), 72.9 (*C-9*), 57.4 (*C-1*), 41.7 (*C-13*), 40.4 (*C-3*), 37.8 (*C-11*), 37.4 (*C-15*), 31.4 (*C-2*), 29.6 (*C-16*), 27.4 (*C-12*), 22.5 (*C-20*), 18.3 (*C-19*) ppm. **HR-MS (ESI$^+$):** m/z

calc. $C_{20}H_{27}NO_5H$ [M+H]$^+$: 362.1962, found: 362.1955 m/z. **FT-IR:** film, $\tilde{\nu}$ = 3299 (w), 3140 (w), 3007 (w), 2927 (w), 1760 (w), 1706 (s), 1656 (w), 1614 (w), 1518 (w), 1482 (w), 1413 (w), 1362 (w), 1221 (s), 1153 (w), 1116 (w), 1077 (w), 1000 (w), 979 (w), 913 (w), 891 (w), 832 (w), 813 (w), 732 (w), 528 (w), 431 (w) cm^{-1}. **S.r:** $[\alpha]_D^{20}$=+8.9 (*c* 0.07, MeOH). Lit.[6] $[\alpha]_D^{20}$=+7.0 (*c* 0.07, MeOH). **UV:** MeOH (c 0.05 × 10^{-3} M) λ_{max} (log ε) 273 (2.80) nm. **CD:** MeOH (*c* 2.8 × 10^{-3} M) λ_{max} (Δε) 217 (−7.214), 273 (+16.50) nm.

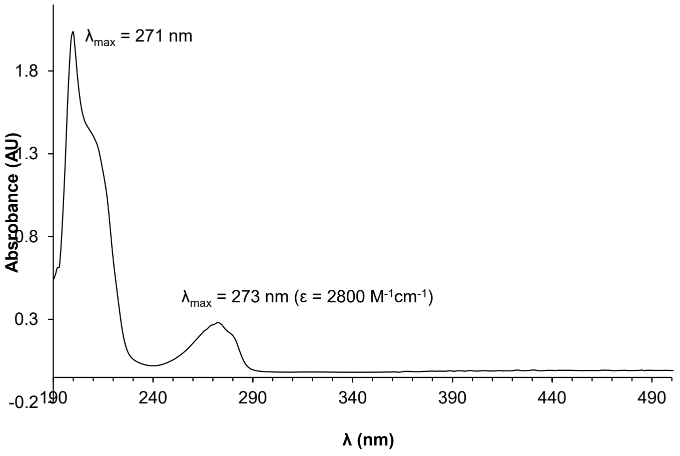

Figure S1: UV-Vis spectrum of synthetic voratin C (**3**) in MeOH (c 0.05 mM) at rt (ε = 2800 M^{-1} cm^{-1} at 273 nm) with a cell path of 0.2 cm.

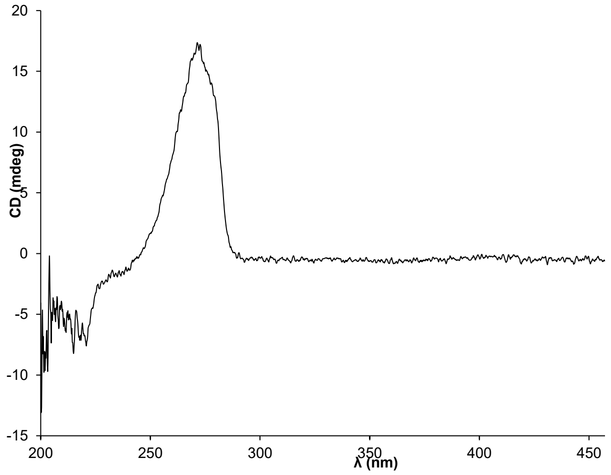

Figure S2: CD spectrum of synthetic voratin C (**3**) in MeOH (c 2.8 mM) at rt with a cell path of 1.0 mm.

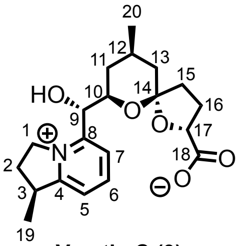

Voratin C (3)

no.	Isolated product[7] MeOH-d$_4$ (500 MHz) δH (*J* in Hz)	synthetic product MeOH-d$_4$ (500 MHz) δH (*J* in Hz)	Isolated product[7] MeOH-d$_4$ (125 MHz) δC	synthetic product MeOH-d$_4$ (126 MHz) δC
**1**	a: 5.05, ddd (12.7, 9.1, 3.7)<br>b: 4.70 ddd (12.7, 8.8, 8.1)	a: 5.05, ddd (12.5, 9.2, 3.5)<br>b: 4.70 ddd (12.5, 8.7, 8.1)	57.3	57.3
**2**	a: 2.74, dtd, (12.9, 8.1, 3.7)<br>b: 2.02, dq (12.9, 8.8)	a: 2.74, dtd, (12.9, 8.5, 3.8)<br>b: 2.02, dq (12.6, 8.8)	31.4	31.4
**3**	3.78, tq (8.8, 7.1)	3.78, tq (8.8, 7.1)	40.4	40.4
**4**	—	—	162.8	162.9
**5**	7.95, d (8.1)	7.96, d (8.1)	123.3	123.2
**6**	8.52, t (8.1)	8.52, t (8.1)	147.0	147.0
**7**	8.03, d (8.1)	8.04, d (8.1)	124.8	124.7
**8**	—	—	159.8	159.9
**9**	4.90, d (8.8)	4.90, d (8.9)	73.0	72.9
**10**	3.82, ddd (11.0, 8.8, 2.0)	3.82, ddd (11.0, 8.8, 1.8)	76.5	76.6
**11**	a: 2.16, br d (12.5)<br>b: 1.07 ddd (12.5, 12.5, 12.5)	a: 2.16, br d (12.8)<br>b: 1.07 dd (12.1, 12.0)	37.8	37.8
**12**	2.09, m	2.09, m	27.4	27.4
**13**	a: 1.90, dd (12.7, 2.7)<br>b: 1.31, t (12.7)	a: 1.90, dd (13.0, 2.8)<br>b: 1.34, t (12.8)	41.8	41.7
**14**	—	—	108.8	108.8
**15**	1.68, m<br>1.66, m	1.68, m<br>1.66, m	37.5	37.4
**16**	A: 1.70, m<br>B: 1.57, m	A: 1.70, m<br>B: 1.57, m	29.6	29.6
**17**	3.48, dd (9.1, 5.1)	3.51, dd (9.3, 5.3)	79.1	79.0
**18**	—	—	178.3	178.3[a]
**19**	1.54, d (7.1)	1.54, d (7.1)	18.2	18.3
20	0.98, d (6.4)	0.99, d (6.4)	22.5	22.5

[a] C-18 appeared as a broad signal in our spectra, presumably because of slow relaxation. Assignment was made with HMBC correlation from *H-17*.

3 NMR-Spectra

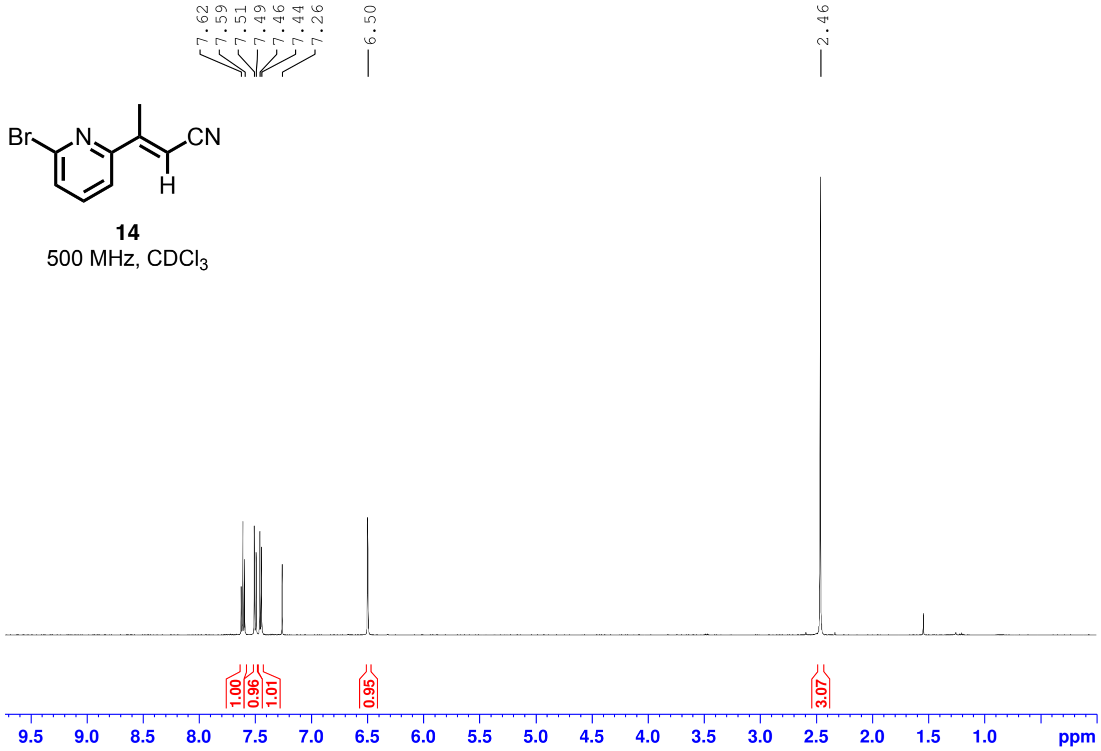

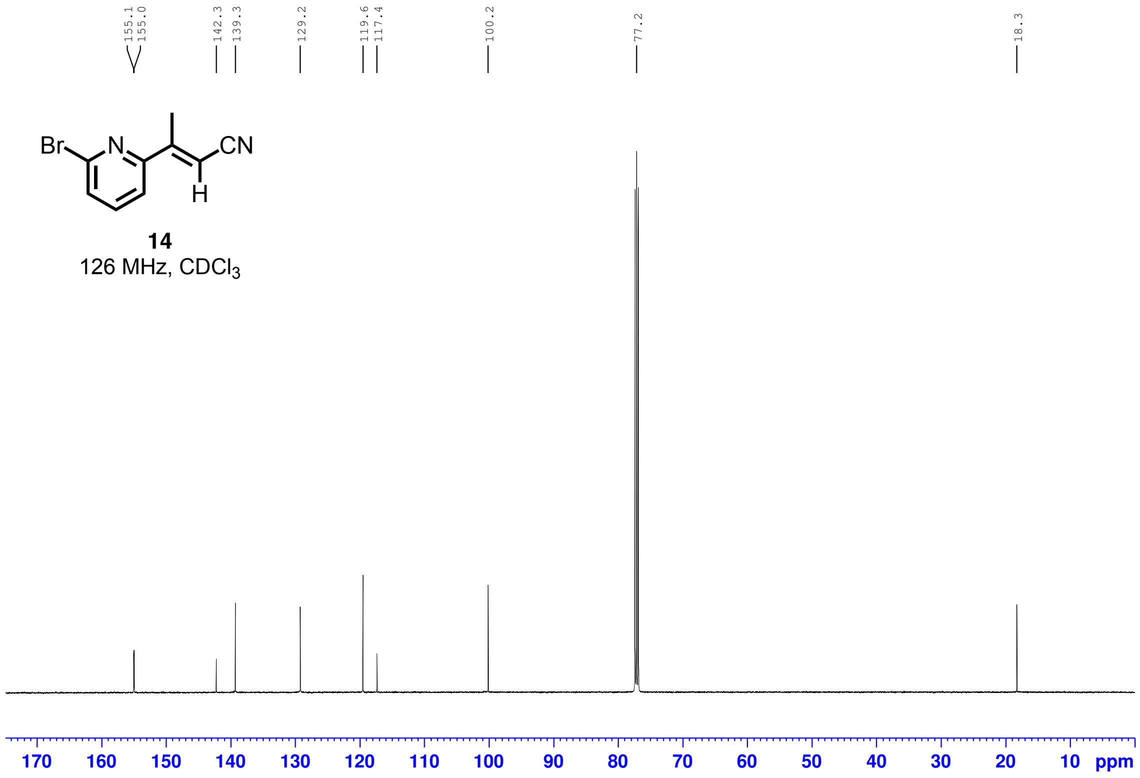

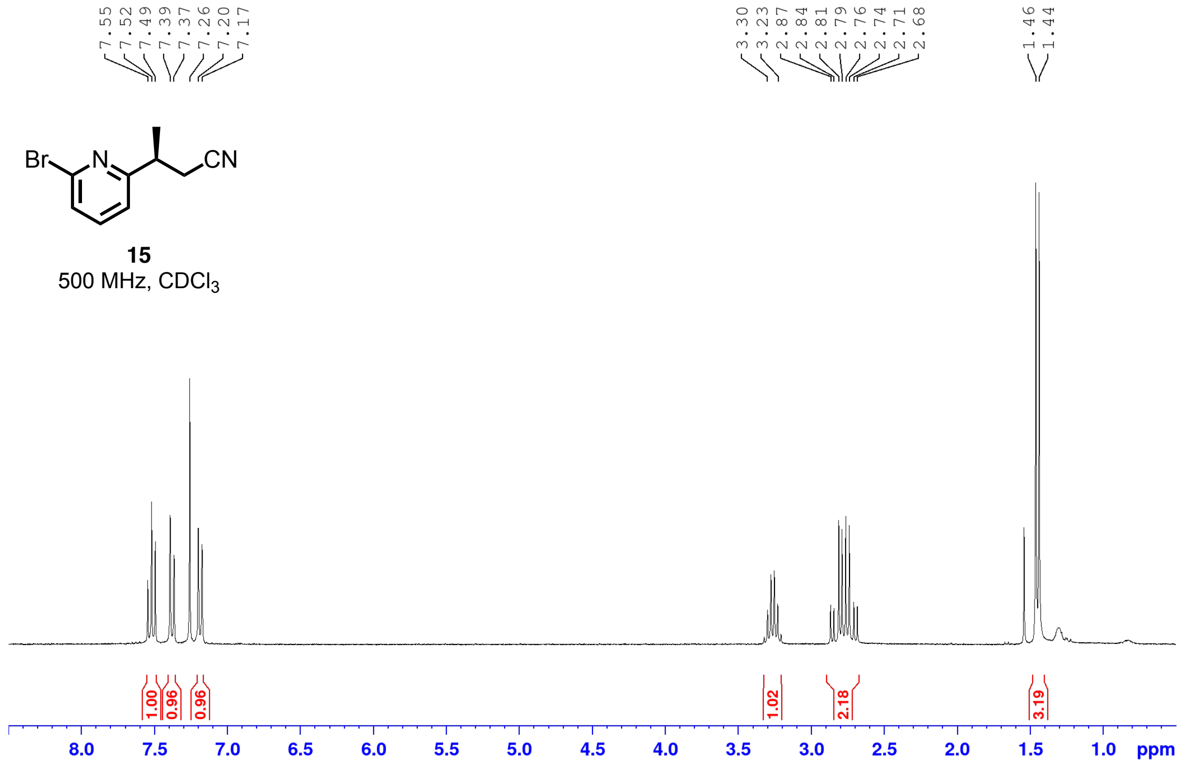

15
500 MHz, $CDCl_3$

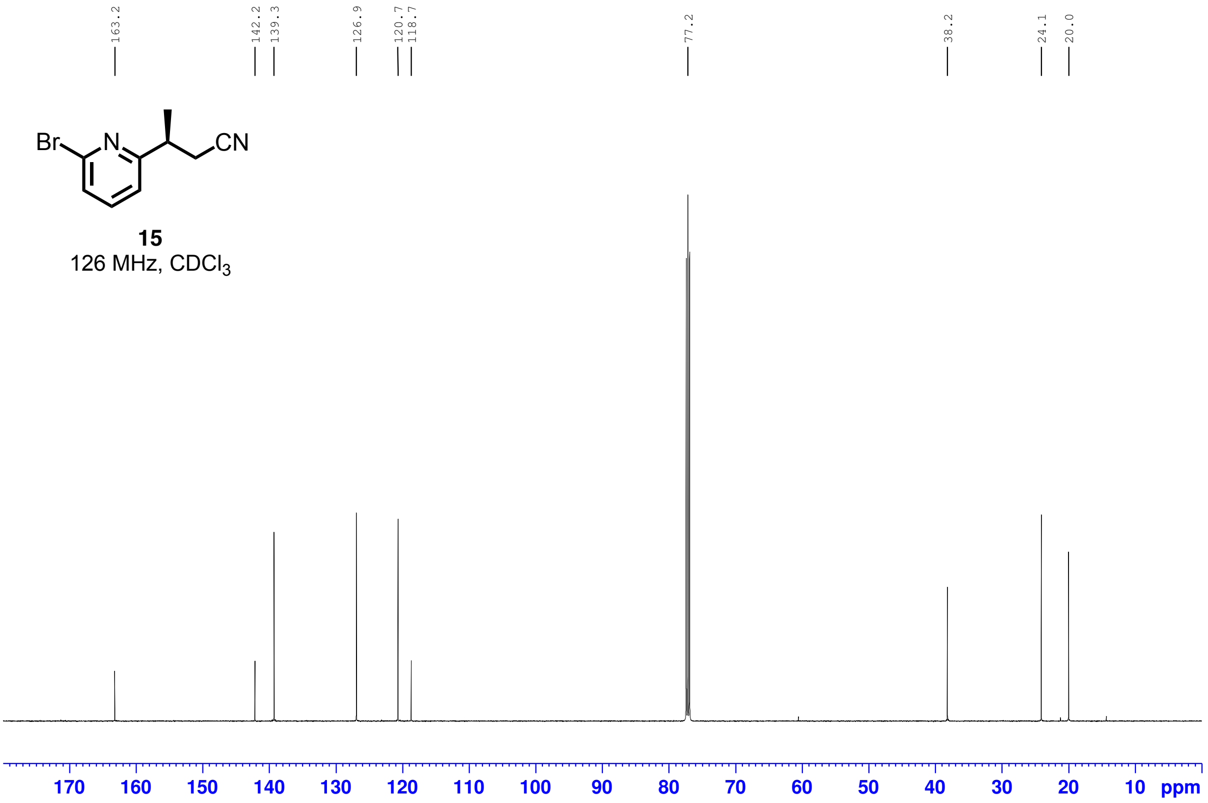

15
126 MHz, $CDCl_3$

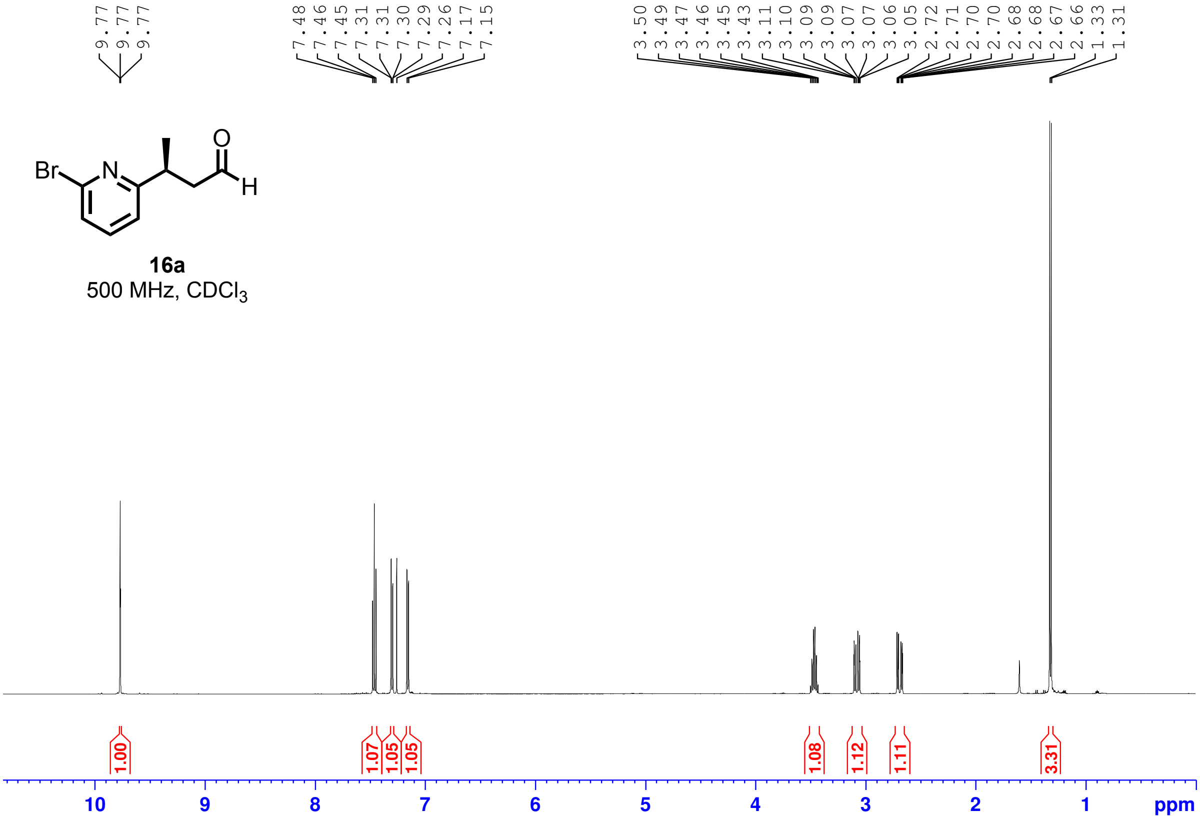
9.77
9.77
9.77
7.48
7.46
7.45
7.31
7.31
7.30
7.29
7.26
7.17
7.15
3.50
3.49
3.47
3.46
3.45
3.43
3.11
3.10
3.09
3.09
3.07
3.07
3.06
3.05
2.72
2.71
2.70
2.70
2.68
2.68
2.67
2.66
1.33
1.31
Br
N
O
H
16a
500 MHz, CDCl3
1.00
1.07
1.05
1.05
1.08
1.12
1.11
3.31
10
9
8
7
6
5
4
3
2
1
ppm

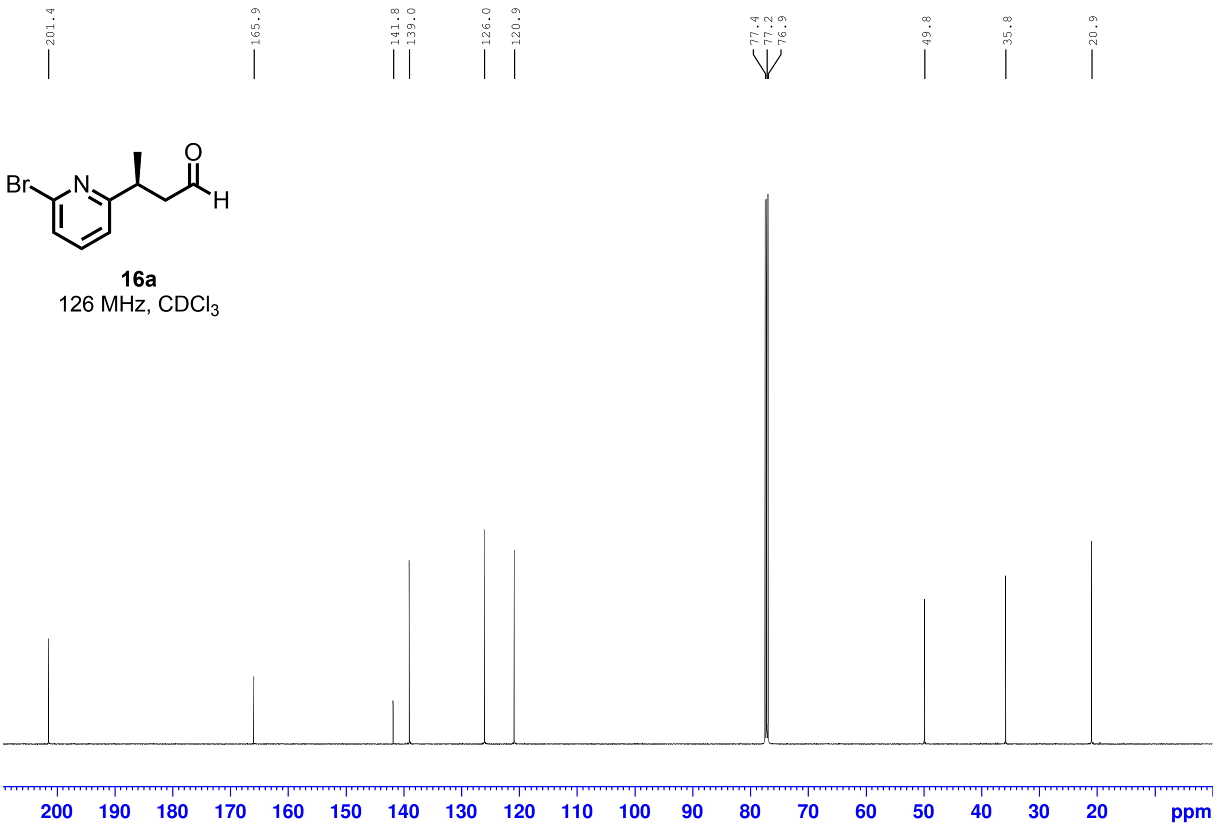
201.4
165.9
141.8
139.0
126.0
120.9
77.4
77.2
76.9
49.8
35.8
20.9
Br
N
O
H
16a
126 MHz, CDCl3
200
190
180
170
160
150
140
130
120
110
100
90
80
70
60
50
40
30
20
ppm

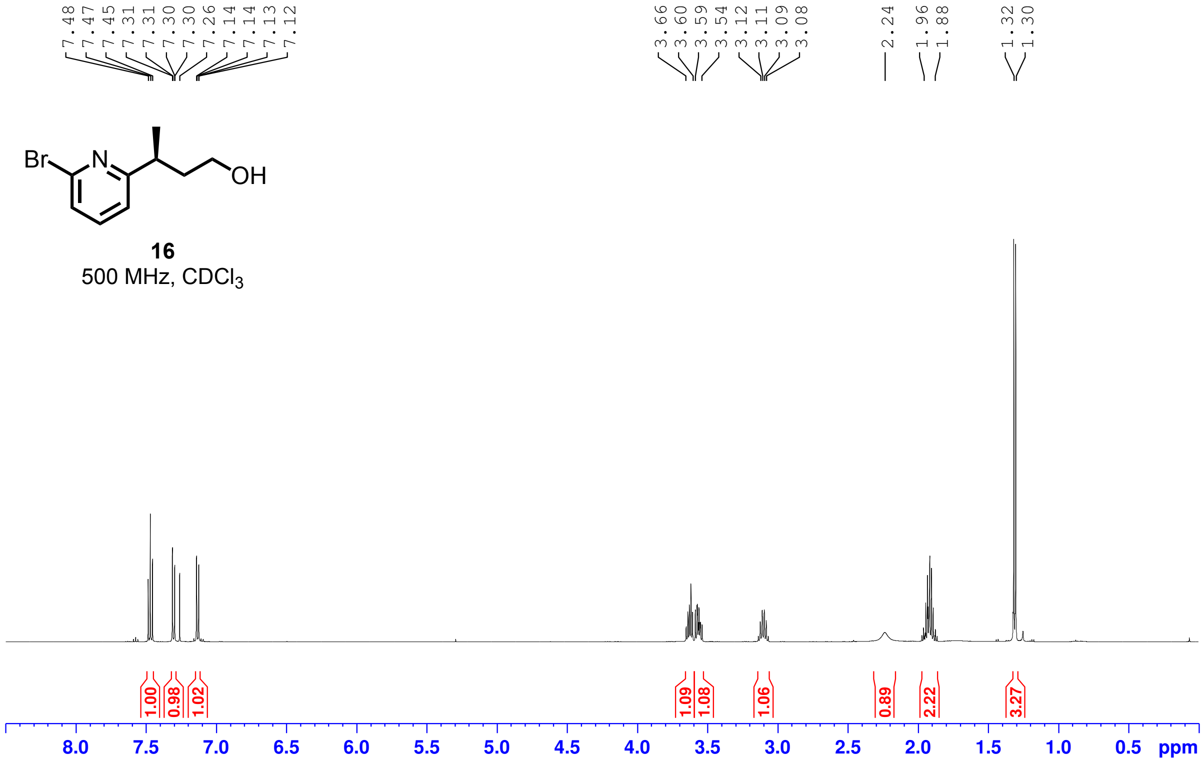
7.48 7.47 7.45 7.31 7.30 7.30 7.26 7.14 7.14 7.13 7.12
3.66 3.60 3.59 3.54 3.12 3.11 3.09 3.08
2.24 1.96 1.88
1.32 1.30
Br
N
OH
16
500 MHz, $CDCl_3$
1.00 0.98 1.02 1.09 1.08 1.06 0.89 2.22 3.27
8.0 7.5 7.0 6.5 6.0 5.5 5.0 4.5 4.0 3.5 3.0 2.5 2.0 1.5 1.0 0.5 ppm

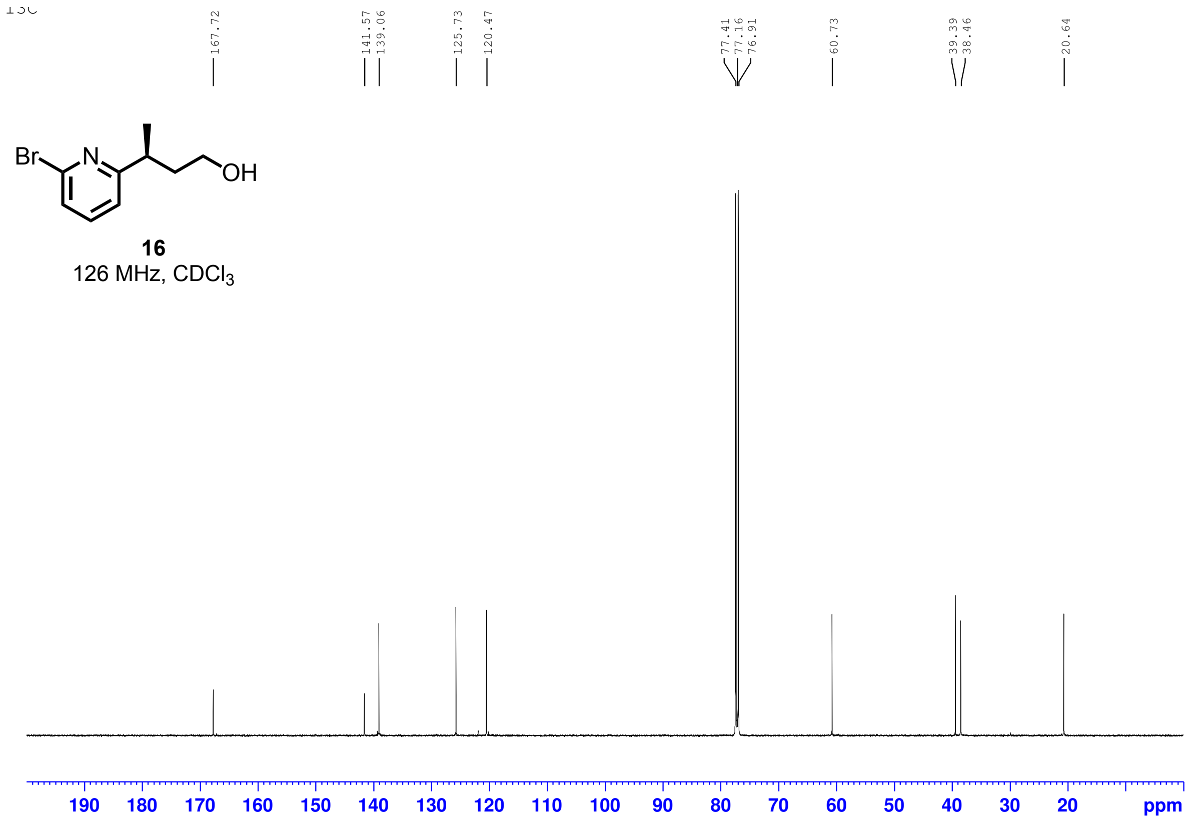
167.72
141.57 139.06
125.73 120.47
77.41 77.16 76.91
60.73
39.39 38.46
20.64
Br
N
OH
16
126 MHz, $CDCl_3$
190 180 170 160 150 140 130 120 110 100 90 80 70 60 50 40 30 20 ppm

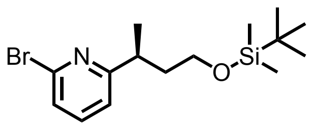

17
500 MHz, $CDCl_3$

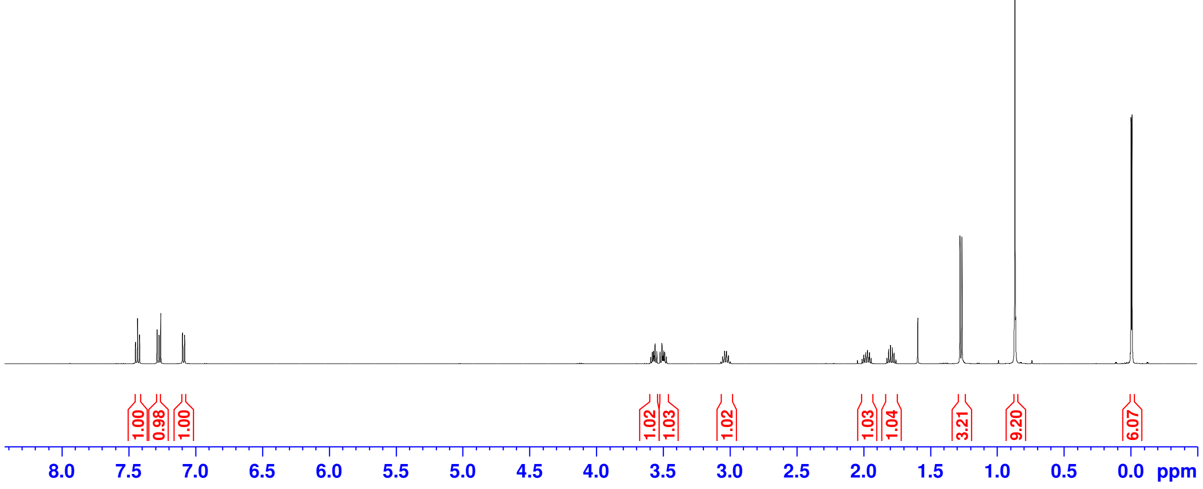

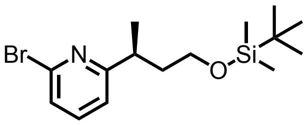

17
126 MHz, $CDCl_3$

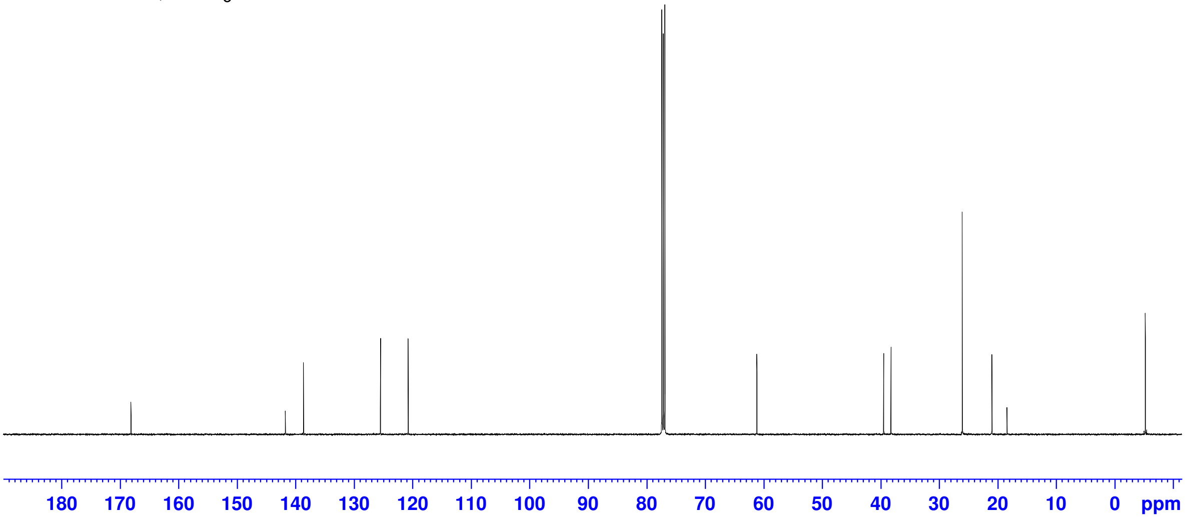

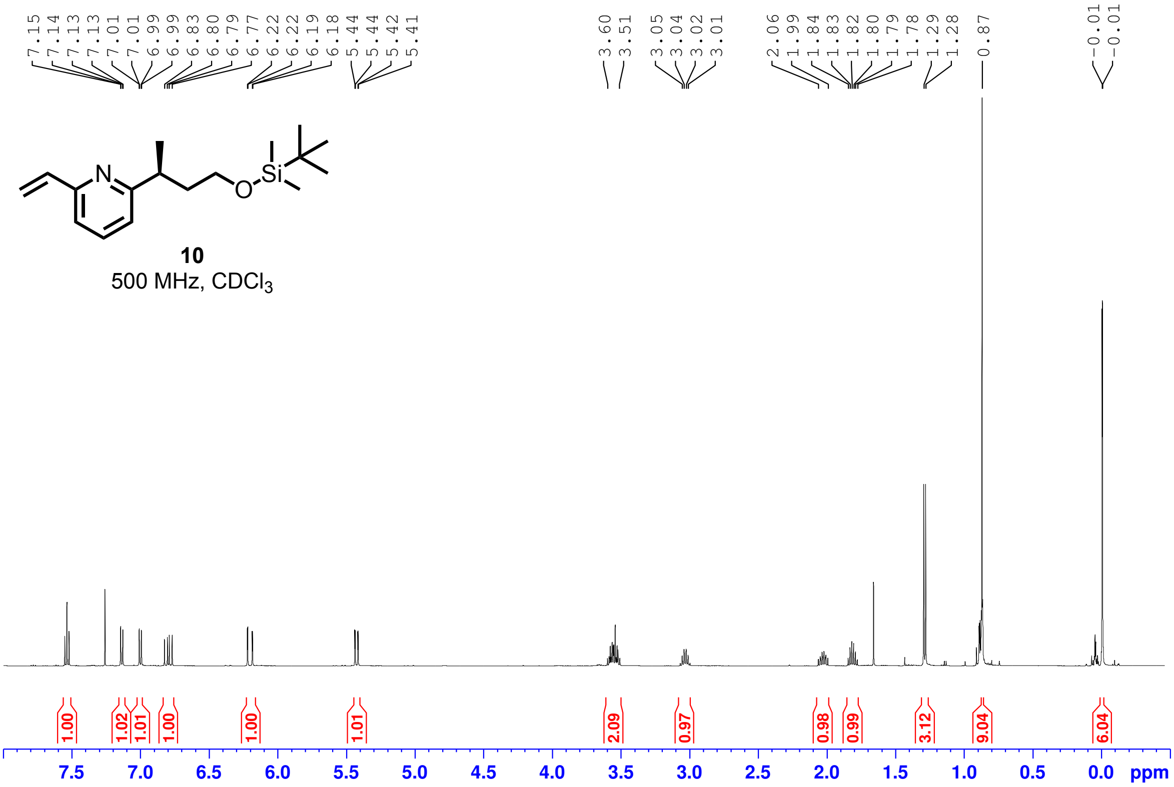
10
500 MHz, CDCl3
ppm

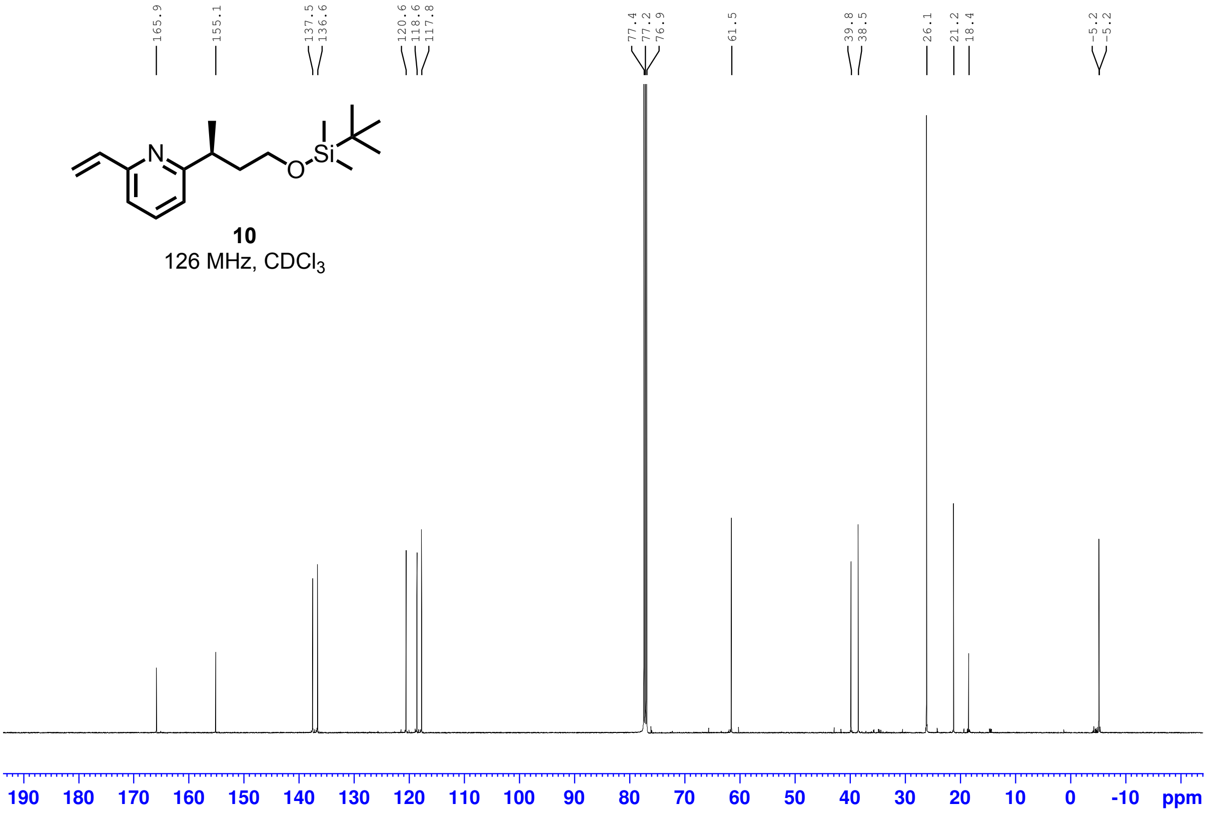
10
126 MHz, CDCl3
ppm

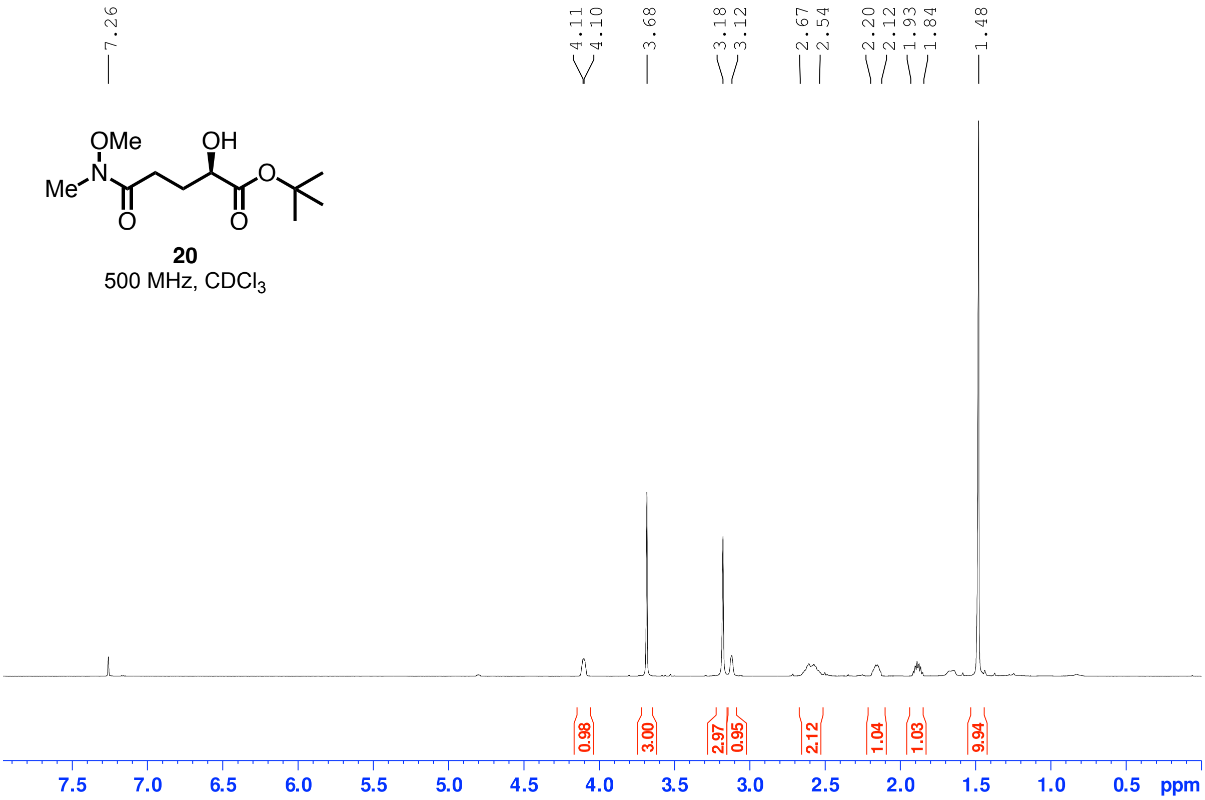
7.26
4.11
4.10
3.68
3.18
3.12
2.67
2.54
2.20
2.12
1.93
1.84
1.48
OMe
OH
N
Me
O
O
O
20
500 MHz, $CDCl_3$
0.98
3.00
2.97
0.95
2.12
1.04
1.03
9.94
7.5 7.0 6.5 6.0 5.5 5.0 4.5 4.0 3.5 3.0 2.5 2.0 1.5 1.0 0.5 ppm

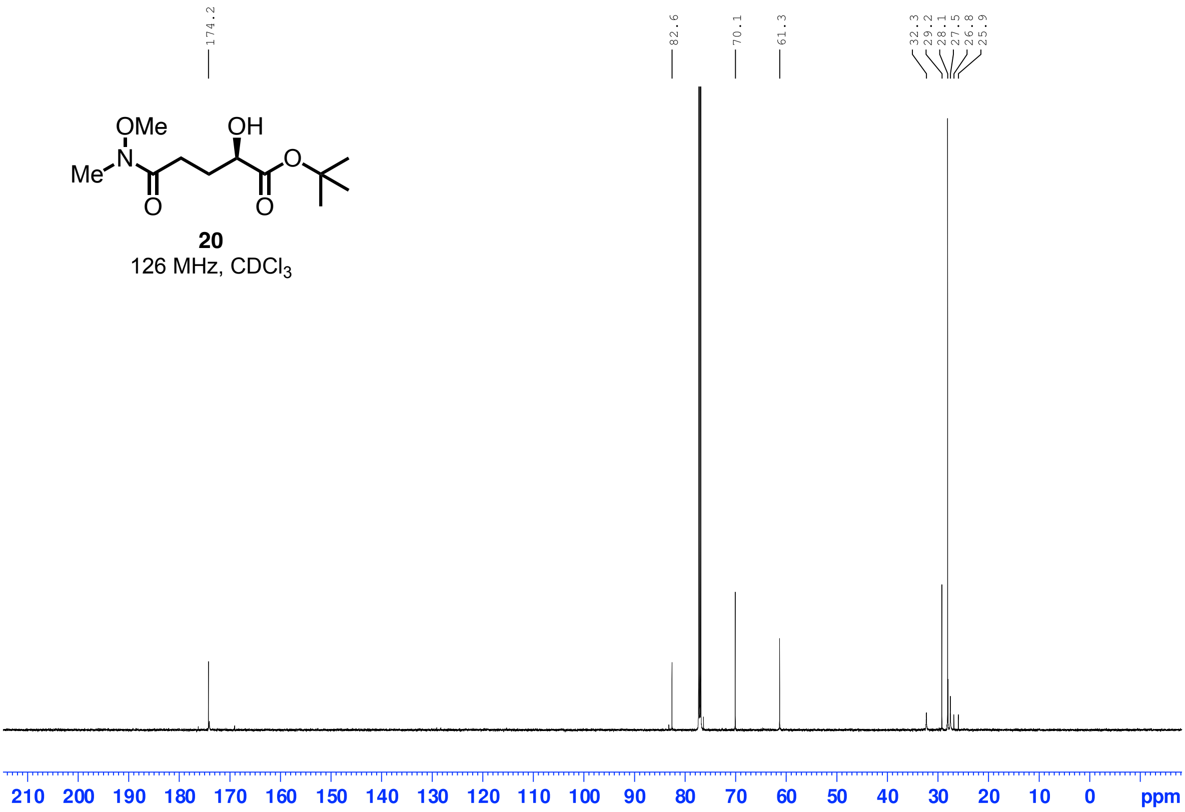
174.2
82.6
70.1
61.3
32.3
29.2
28.1
27.5
26.8
25.9
OMe
OH
N
Me
O
O
O
20
126 MHz, $CDCl_3$
210 200 190 180 170 160 150 140 130 120 110 100 90 80 70 60 50 40 30 20 10 0 ppm

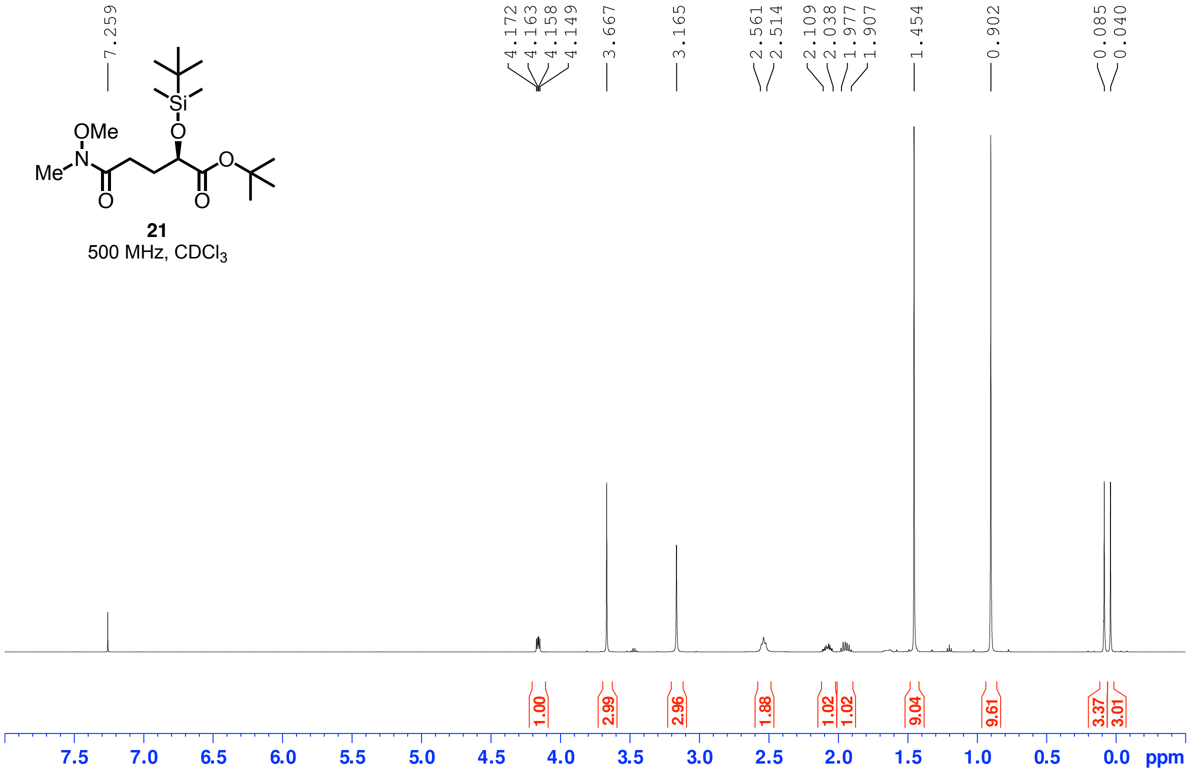

OMe
Me
N
O
Si
O
O
21
500 MHz, CDCl3
7.259
4.172
4.163
4.158
4.149
3.667
3.165
2.561
2.514
2.109
2.038
1.977
1.907
1.454
0.902
0.085
0.040
1.00
2.99
2.96
1.88
1.02
1.02
9.04
9.61
3.37
3.01
7.5 7.0 6.5 6.0 5.5 5.0 4.5 4.0 3.5 3.0 2.5 2.0 1.5 1.0 0.5 0.0 ppm

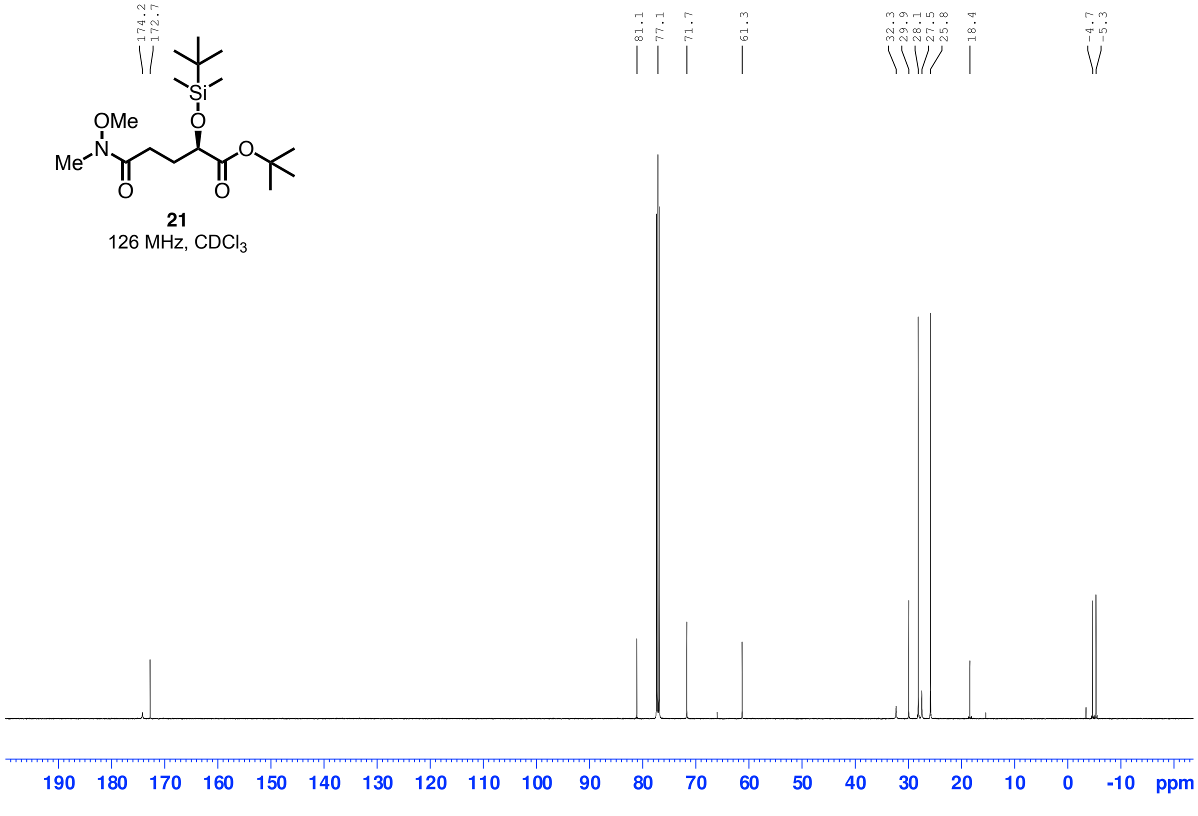

OMe
Me
N
O
Si
O
O
21
126 MHz, CDCl3
174.2
172.7
81.1
77.1
71.7
61.3
32.3
29.9
28.1
27.5
25.8
18.4
-4.7
-5.3
190 180 170 160 150 140 130 120 110 100 90 80 70 60 50 40 30 20 10 0 -10 ppm

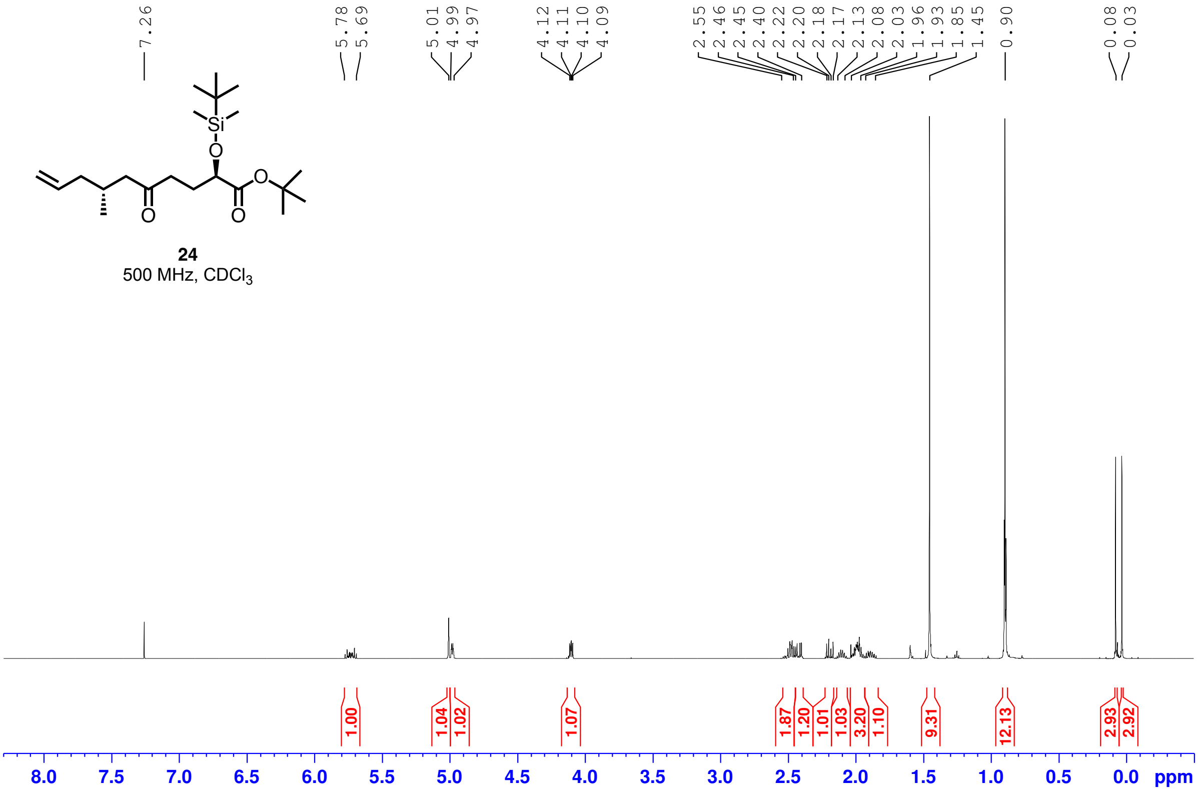
7.26
5.78
5.69
5.01
4.99
4.97
4.12
4.11
4.10
4.09
2.55
2.46
2.45
2.40
2.22
2.20
2.18
2.17
2.13
2.08
2.03
1.96
1.93
1.85
1.45
0.90
0.08
0.03
Si
O
O
O
O
24
500 MHz, $CDCl_3$
1.00
1.04
1.02
1.07
1.87
1.20
1.01
1.03
3.20
1.10
9.31
12.13
2.93
2.92
8.0
7.5
7.0
6.5
6.0
5.5
5.0
4.5
4.0
3.5
3.0
2.5
2.0
1.5
1.0
0.5
0.0
ppm

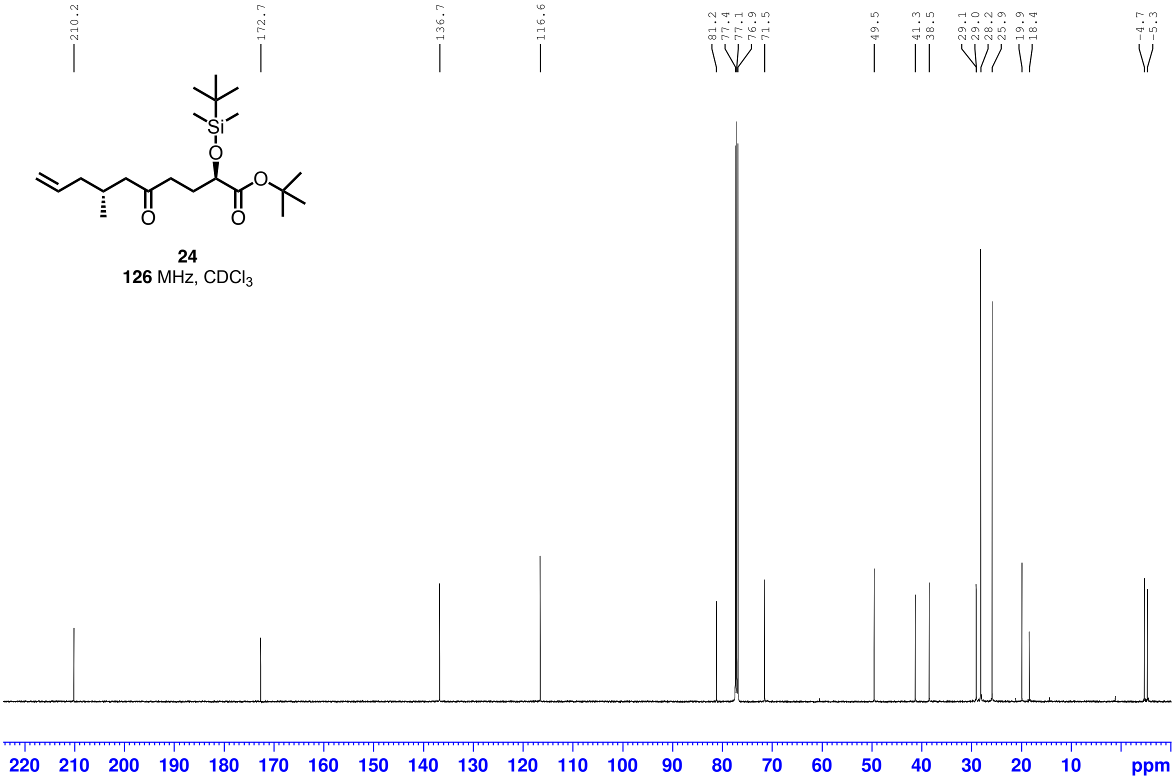
210.2
172.7
136.7
116.6
81.2
77.4
77.1
76.9
71.5
49.5
41.3
38.5
29.1
29.0
28.2
25.9
19.9
18.4
-4.7
-5.3
Si
O
O
O
O
24
126 MHz, $CDCl_3$
220
210
200
190
180
170
160
150
140
130
120
110
100
90
80
70
60
50
40
30
20
10
ppm

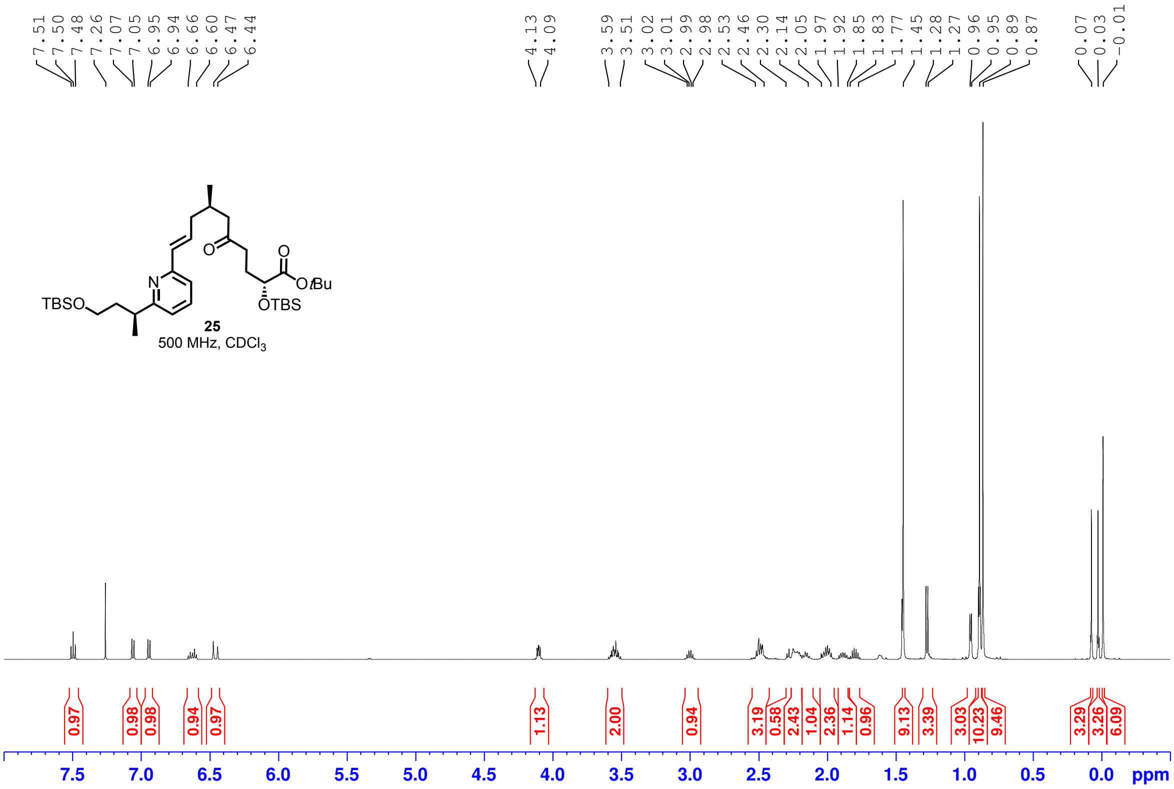
7.51
7.50
7.48
7.26
7.07
7.05
6.95
6.94
6.66
6.60
6.47
6.44
4.13
4.09
3.59
3.51
3.02
3.01
2.99
2.98
2.53
2.46
2.30
2.14
2.05
1.97
1.92
1.85
1.83
1.77
1.45
1.28
1.27
0.96
0.95
0.89
0.87
0.07
0.03
-0.01
TBSO
N
O
O
O*t*Bu
OTBS
25
500 MHz, CDCl3
0.97
0.98
0.98
0.94
0.97
1.13
2.00
0.94
3.19
0.58
2.43
1.04
2.36
1.14
0.96
9.13
3.39
3.03
10.23
9.46
3.29
3.26
6.09
7.5
7.0
6.5
6.0
5.5
5.0
4.5
4.0
3.5
3.0
2.5
2.0
1.5
1.0
0.5
0.0
ppm

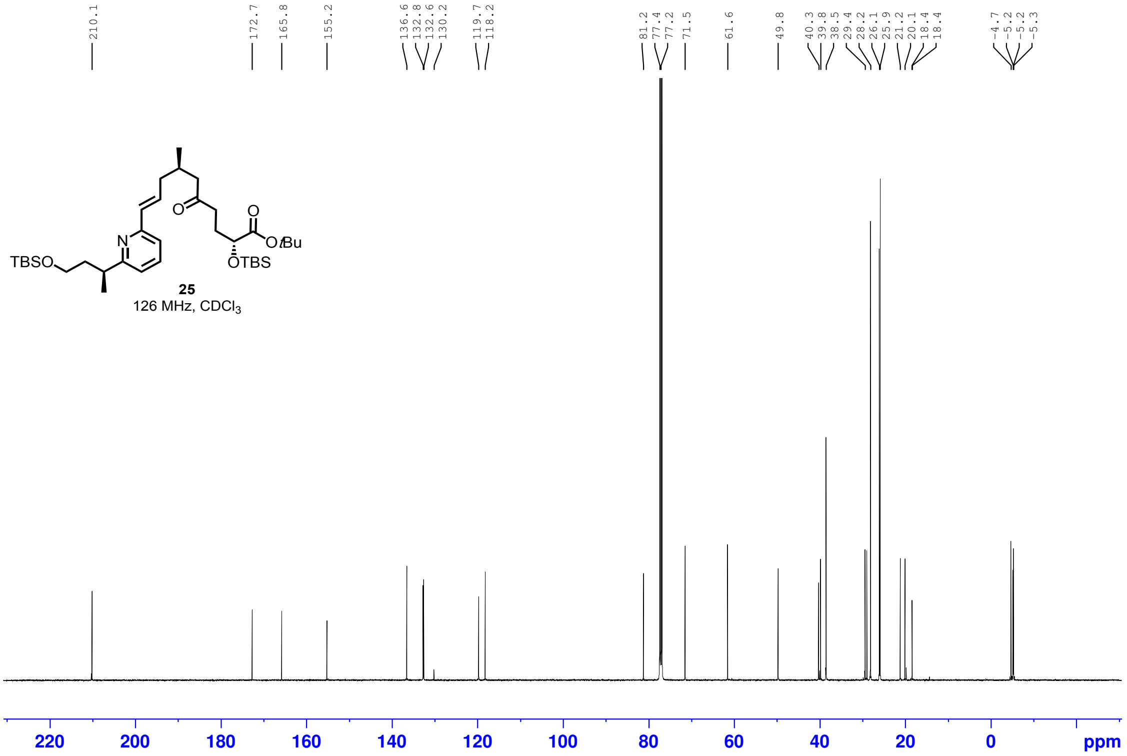
210.1
172.7
165.8
155.2
136.6
132.8
132.6
130.2
119.7
118.2
81.2
77.4
77.2
71.5
61.6
49.8
40.3
39.8
38.5
29.4
28.2
26.1
25.9
21.2
20.1
18.4
18.4
-4.7
-5.2
-5.2
-5.3
TBSO
N
O
O
O*t*Bu
OTBS
25
126 MHz, CDCl3
220
200
180
160
140
120
100
80
60
40
20
0
ppm

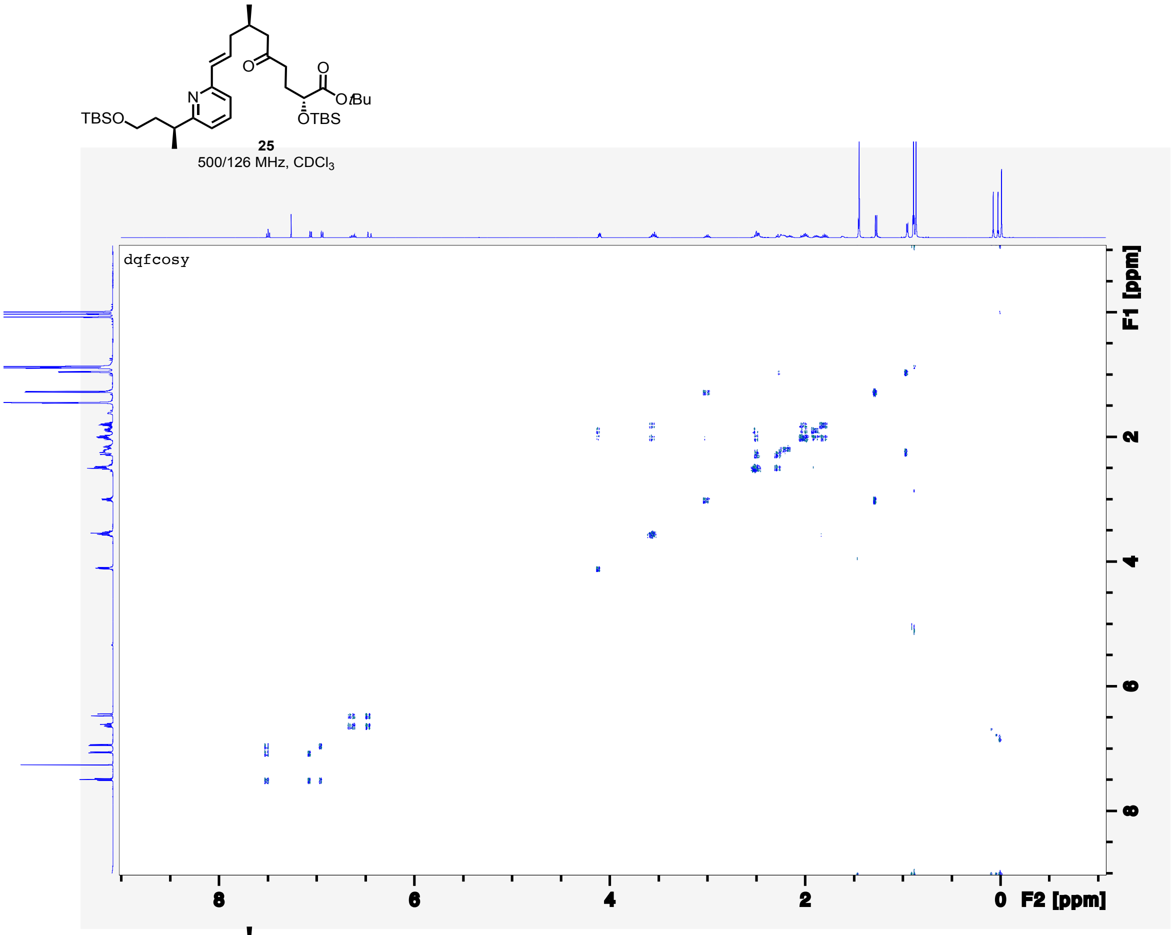
TBSO
N
O
O
OtBu
OTBS
25
500/126 MHz, CDCl3
dqfcosy
F1 [ppm]
F2 [ppm]

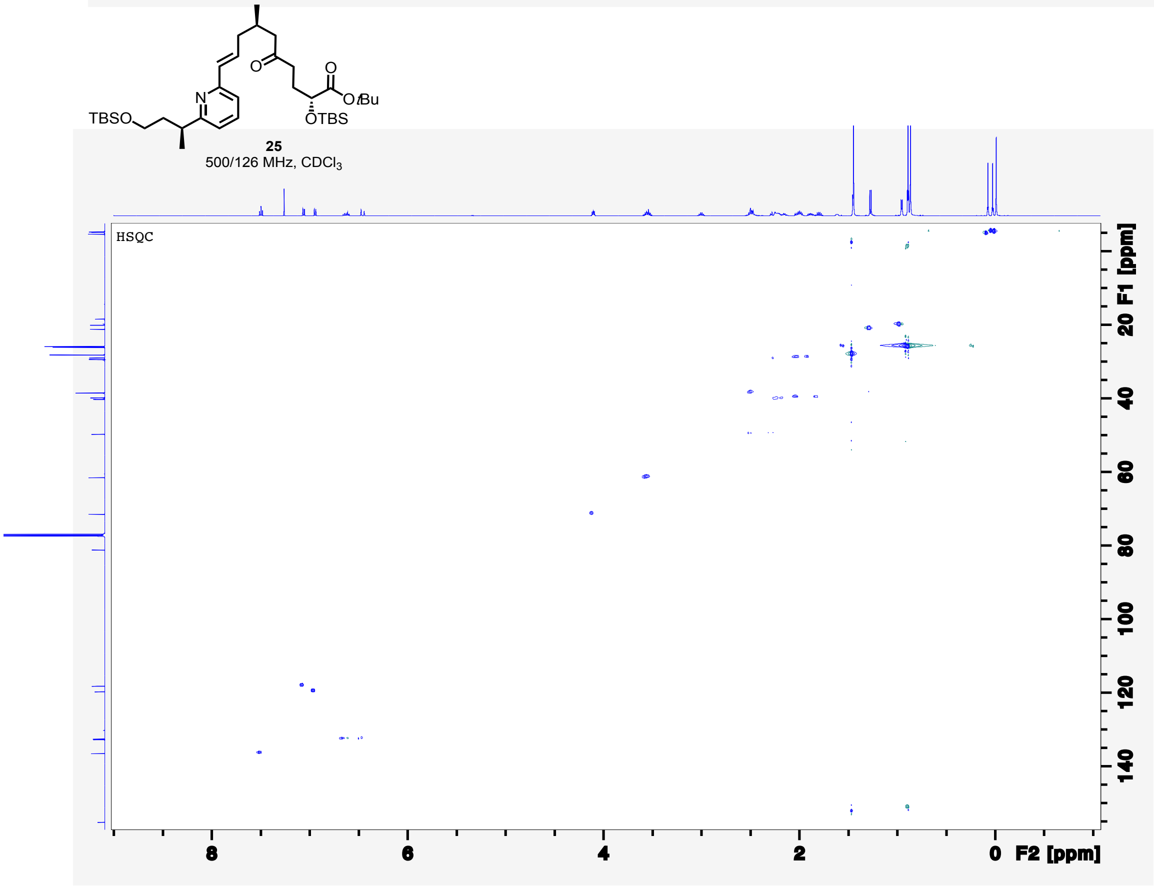
TBSO
N
O
O
OtBu
OTBS
25
500/126 MHz, CDCl3
HSQC
F1 [ppm]
F2 [ppm]

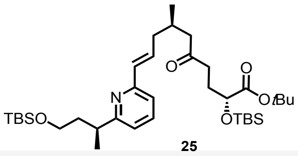

25
500/126 MHz, $CDCl_3$

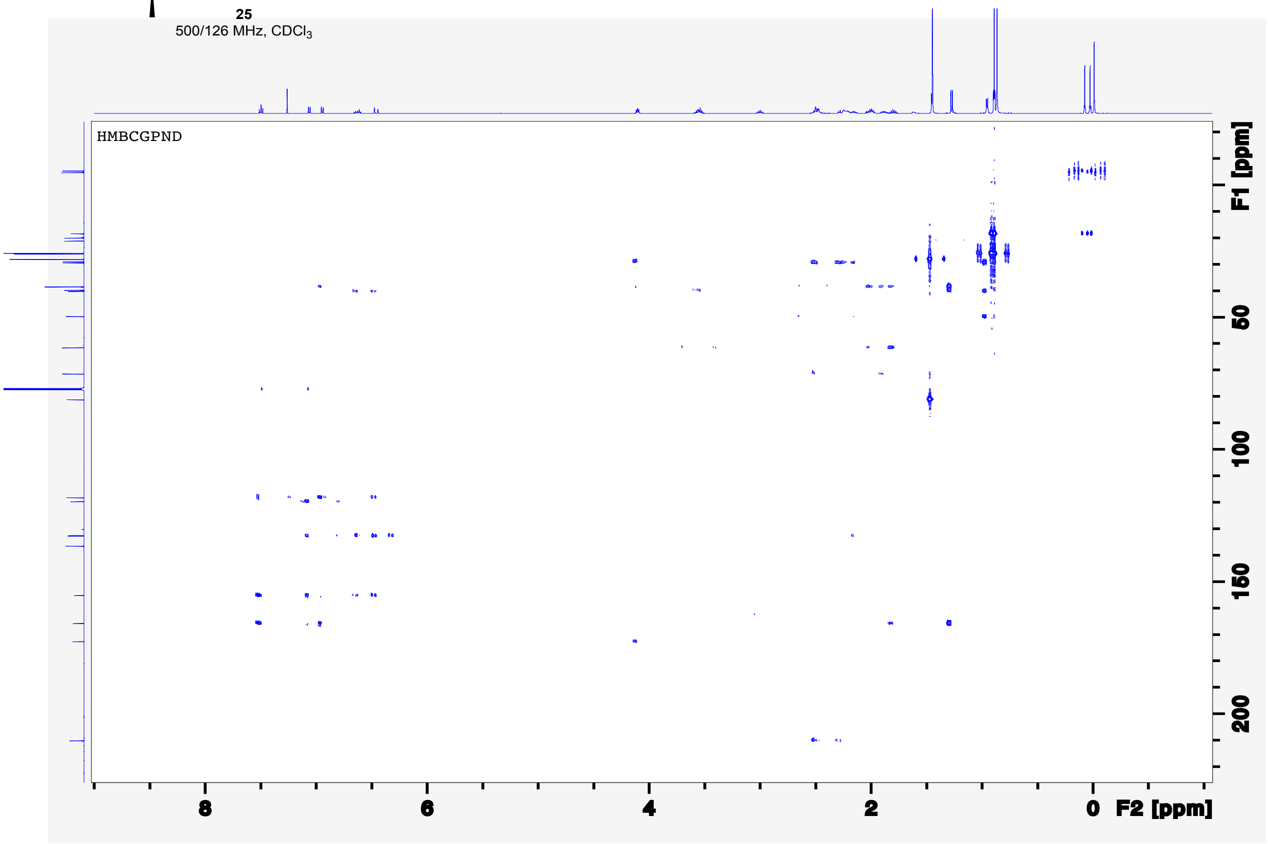

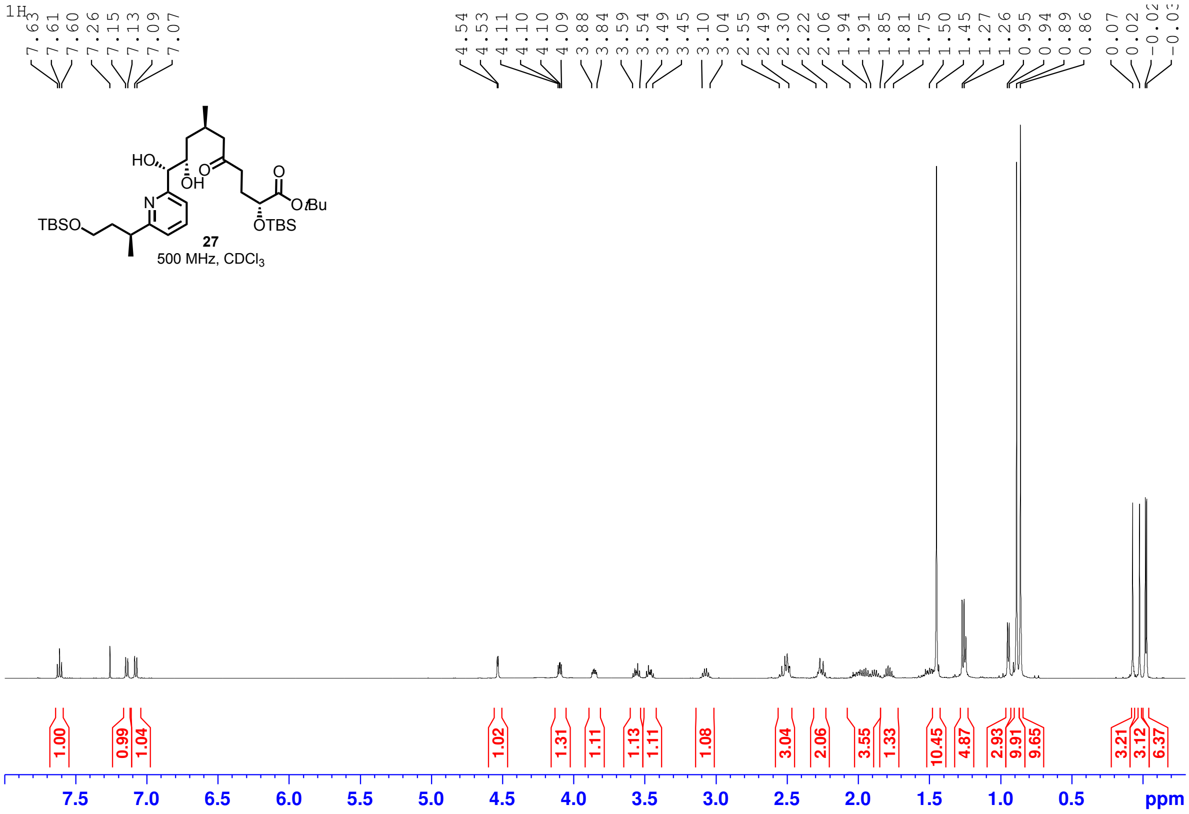
1H
7.63
7.61
7.60
7.26
7.15
7.13
7.09
7.07
4.54
4.53
4.11
4.10
4.10
4.09
3.88
3.84
3.59
3.54
3.49
3.45
3.10
3.04
2.55
2.49
2.30
2.22
2.06
1.94
1.91
1.85
1.81
1.75
1.50
1.45
1.27
1.26
0.95
0.94
0.89
0.86
0.07
0.02
HO
OH
O
O
N
O*t*Bu
OTBS
TBSO
27
500 MHz, CDCl3
1.00
0.99
1.04
1.02
1.31
1.11
1.13
1.11
1.08
3.04
2.06
3.55
1.33
10.45
4.87
2.93
9.91
9.65
3.21
3.12
6.37
7.5
7.0
6.5
6.0
5.5
5.0
4.5
4.0
3.5
3.0
2.5
2.0
1.5
1.0
0.5
ppm

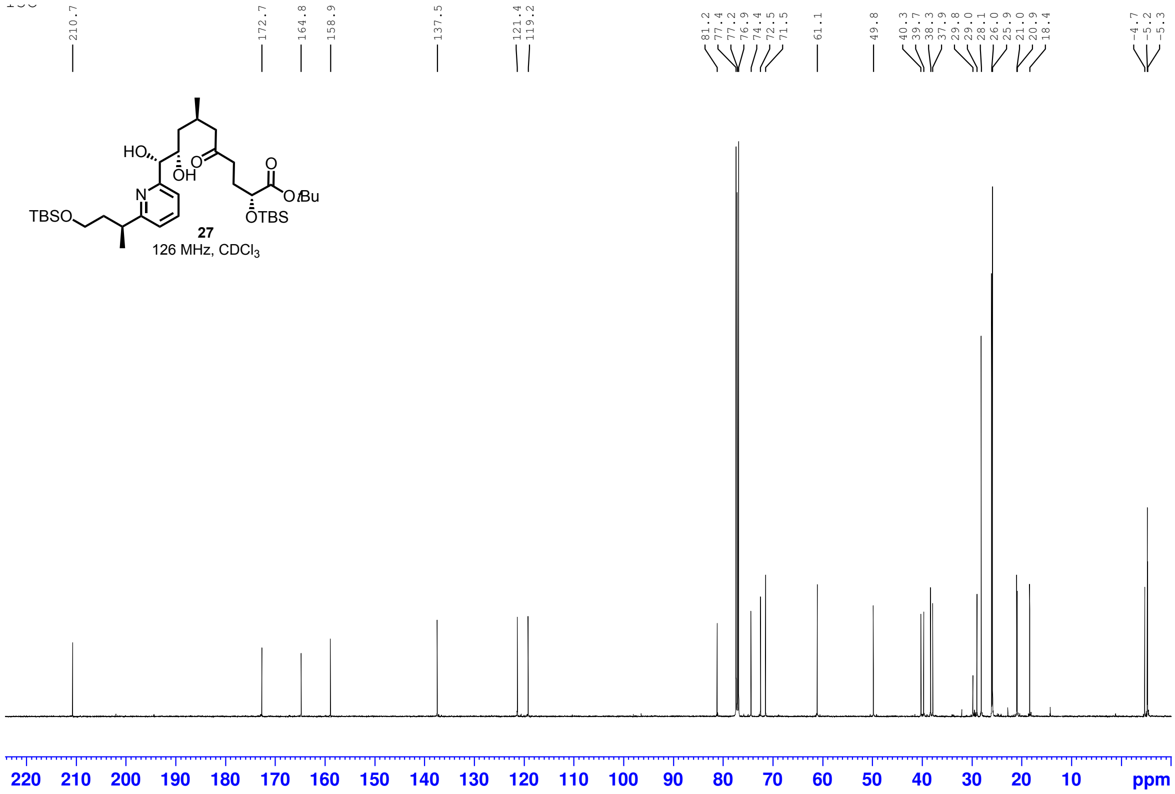
210.7
172.7
164.8
158.9
137.5
121.4
119.2
81.2
77.4
77.2
76.9
74.4
72.5
71.5
61.1
49.8
40.3
39.7
38.3
37.9
29.8
29.0
28.1
26.0
25.9
21.0
20.9
18.4
-4.7
-5.2
-5.3
HO
OH
O
O
N
O*t*Bu
OTBS
TBSO
27
126 MHz, CDCl3
220
210
200
190
180
170
160
150
140
130
120
110
100
90
80
70
60
50
40
30
20
10
ppm

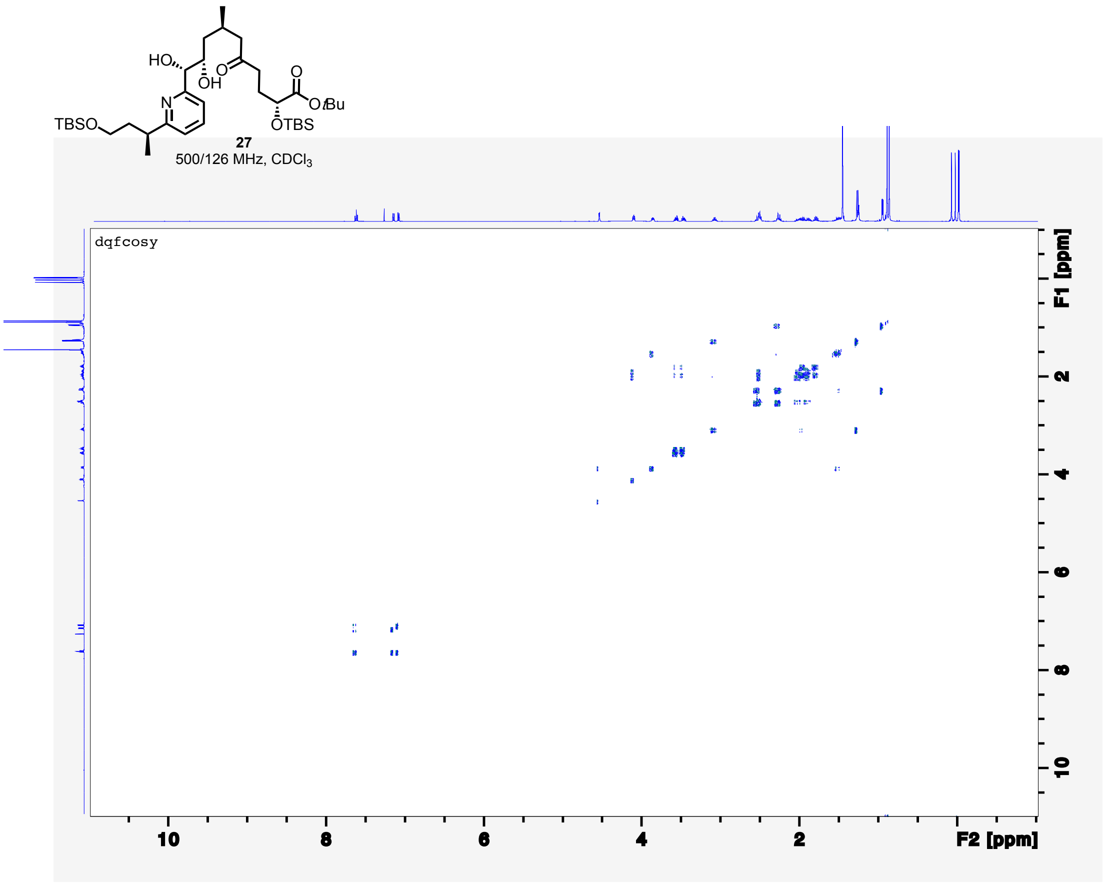

HO
OH
O
O
N
OtBu
TBSO
OTBS
27
500/126 MHz, CDCl3
dqfcosy
F1 [ppm]
F2 [ppm]

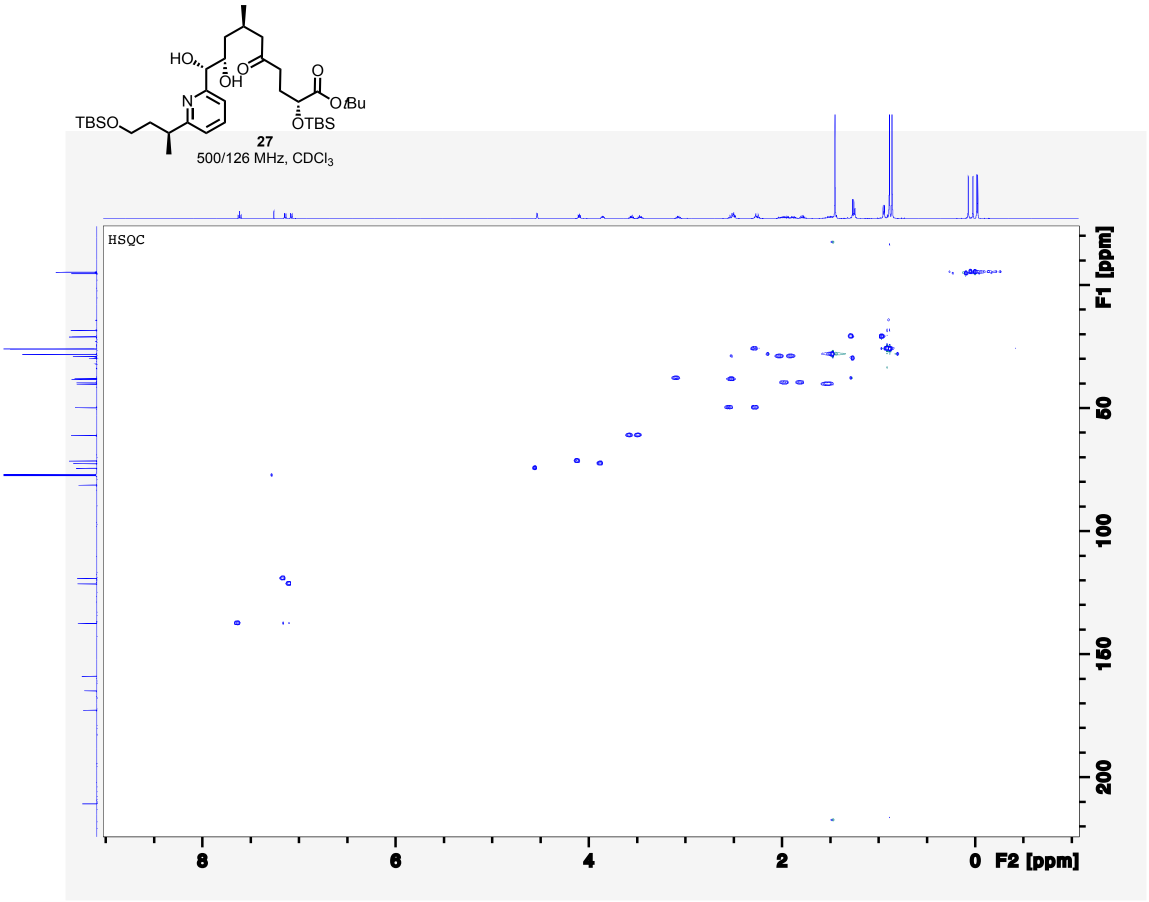

HO
OH
O
O
N
OtBu
TBSO
OTBS
27
500/126 MHz, CDCl3
HSQC
F1 [ppm]
F2 [ppm]

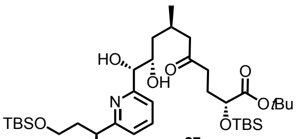

27
500/126 MHz, $CDCl_3$

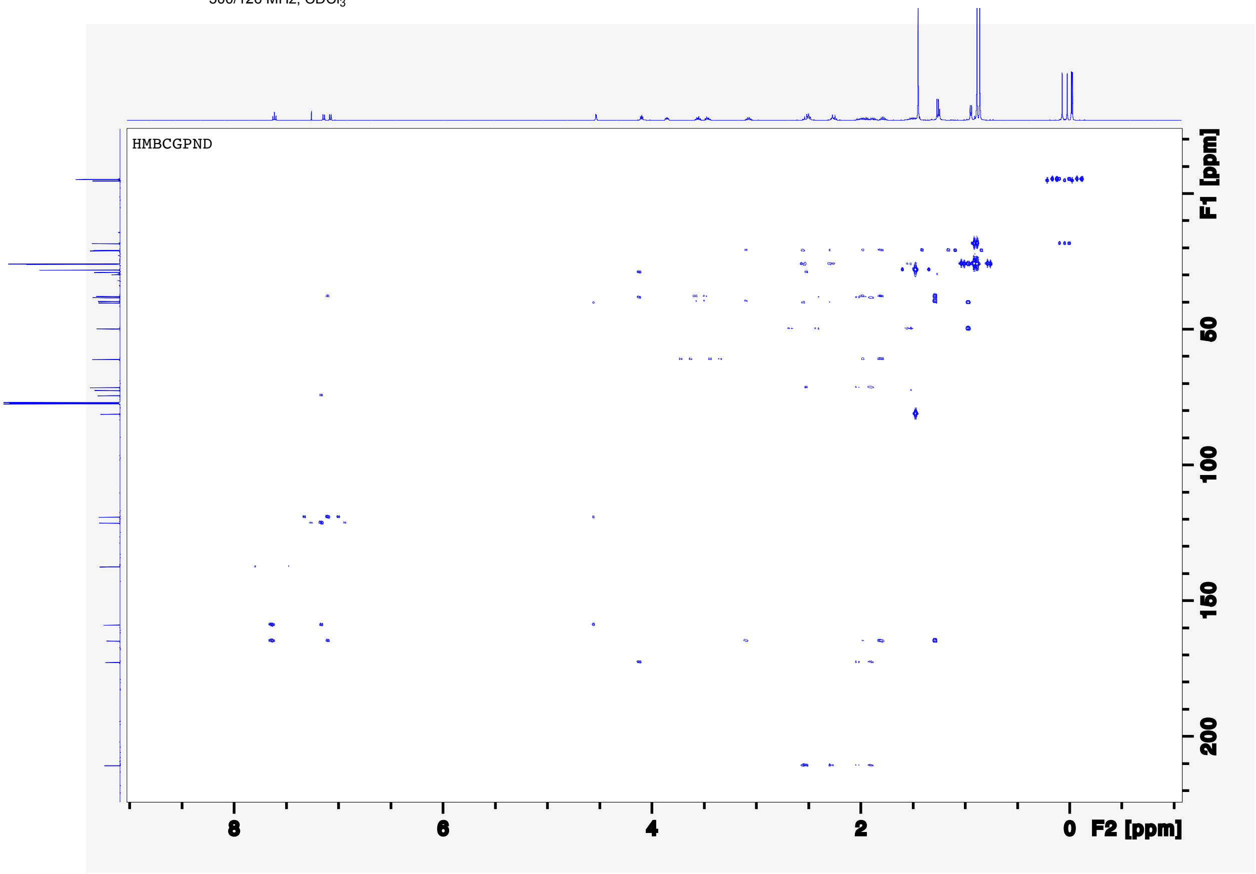

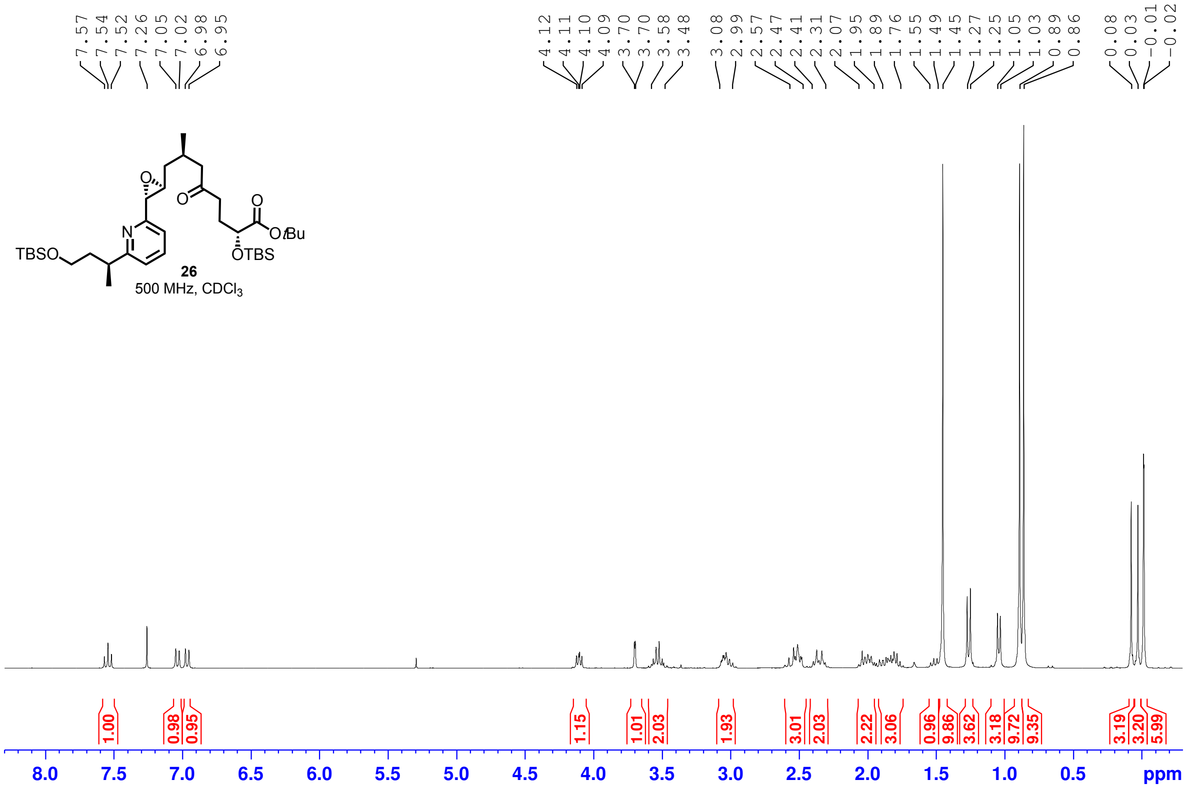

7.57
7.54
7.52
7.26
7.05
7.02
6.98
6.95
4.12
4.11
4.10
4.09
3.70
3.70
3.58
3.48
3.08
2.99
2.57
2.47
2.41
2.31
2.07
1.95
1.89
1.76
1.55
1.49
1.45
1.27
1.25
1.05
1.03
0.89
0.86
0.08
0.03
-0.01
-0.02
TBSO
N
O
O
O
OtBu
OTBS
26
500 MHz, CDCl3
1.00
0.98
0.95
1.15
1.01
2.03
1.93
3.01
2.03
2.22
3.06
0.96
9.86
3.62
3.18
9.72
9.35
3.19
3.20
5.99
8.0
7.5
7.0
6.5
6.0
5.5
5.0
4.5
4.0
3.5
3.0
2.5
2.0
1.5
1.0
0.5
ppm

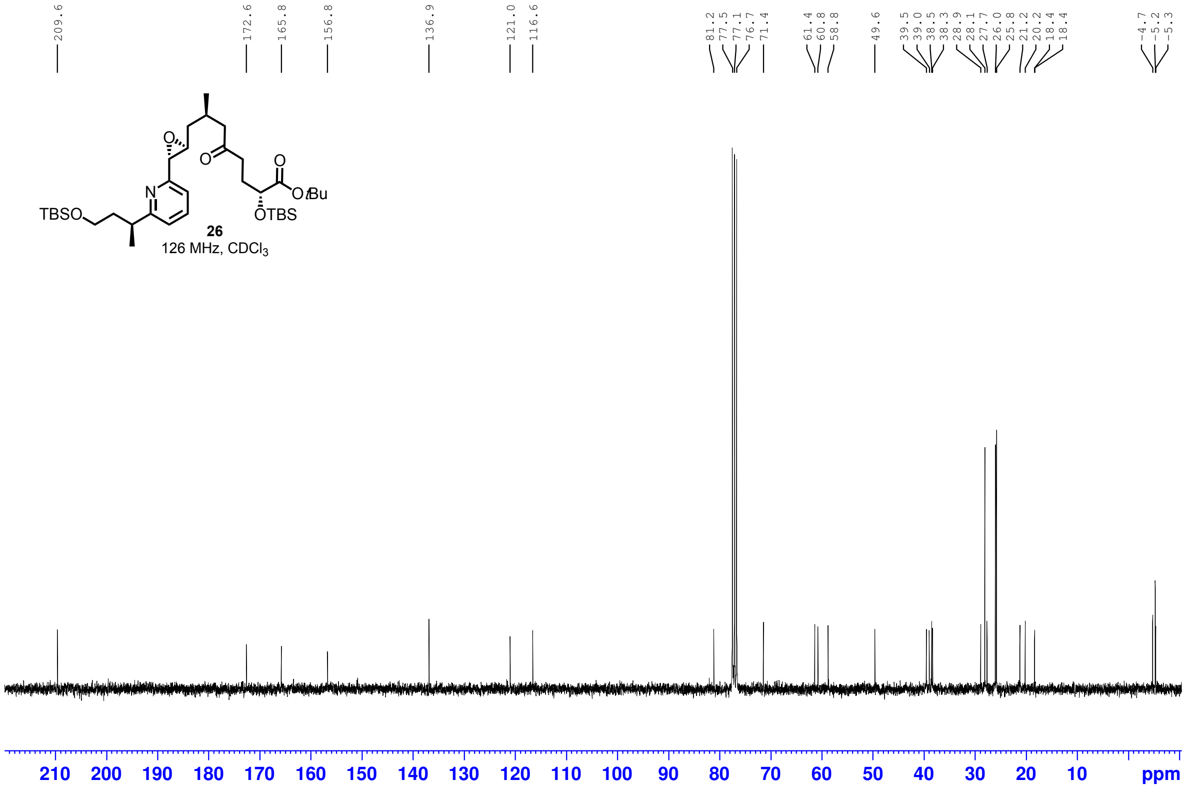

209.6
172.6
165.8
156.8
136.9
121.0
116.6
81.2
77.5
77.1
76.7
71.4
61.4
60.8
58.8
49.6
39.5
39.0
38.5
38.3
28.9
28.1
27.7
26.0
25.8
21.2
20.2
18.4
18.4
-4.7
-5.2
-5.3
TBSO
N
O
O
O
OtBu
OTBS
26
126 MHz, CDCl3
210
200
190
180
170
160
150
140
130
120
110
100
90
80
70
60
50
40
30
20
10
ppm

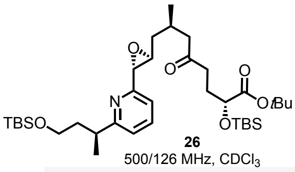

26

500/126 MHz, $CDCl_3$

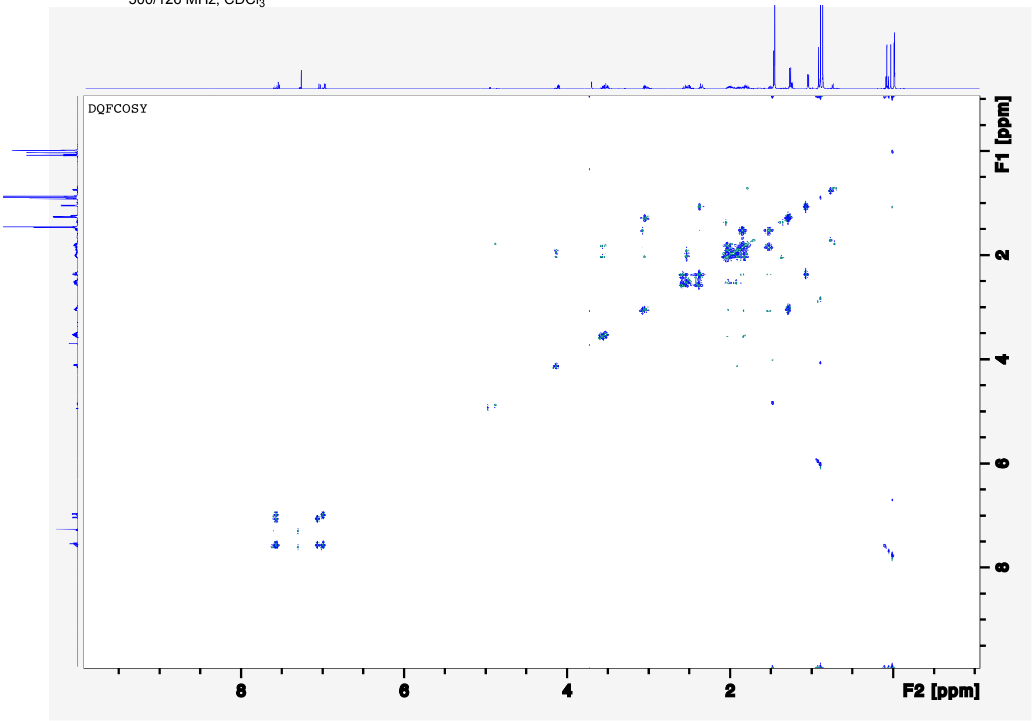

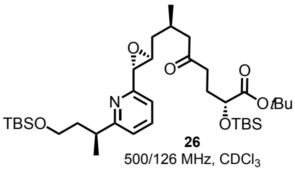

26

500/126 MHz, $CDCl_3$

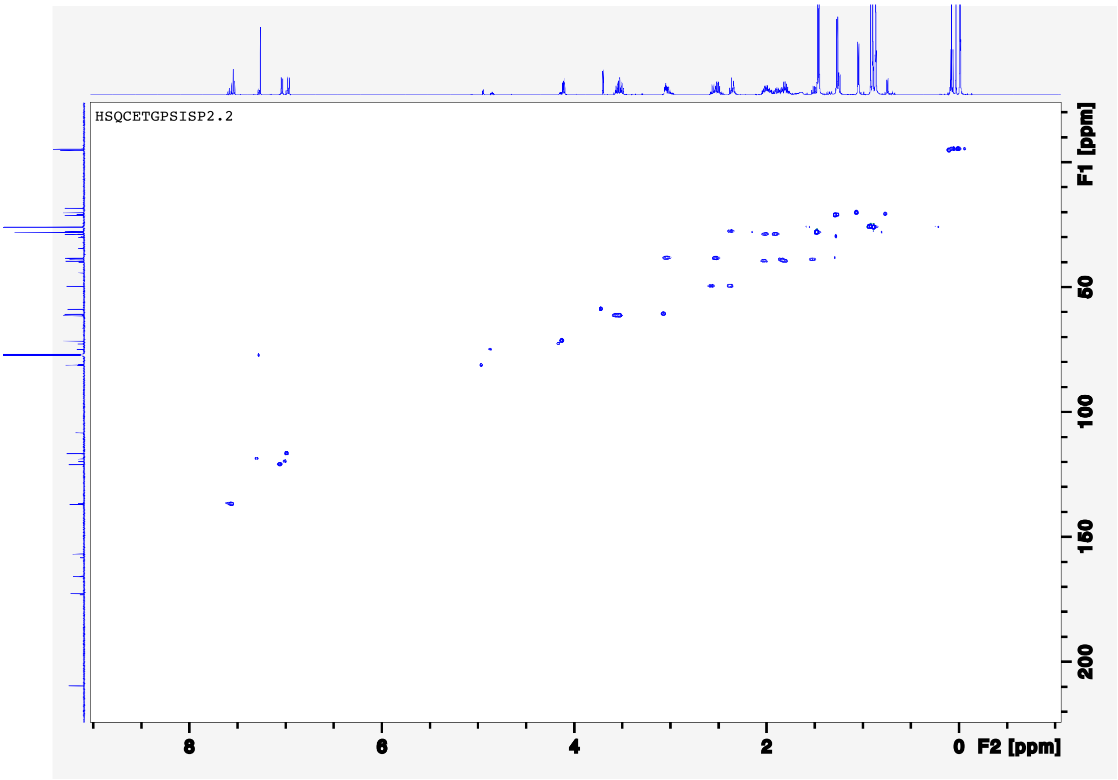

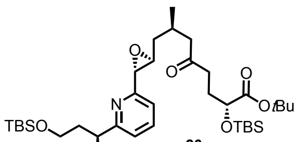

26
500/126 MHz, $CDCl_3$

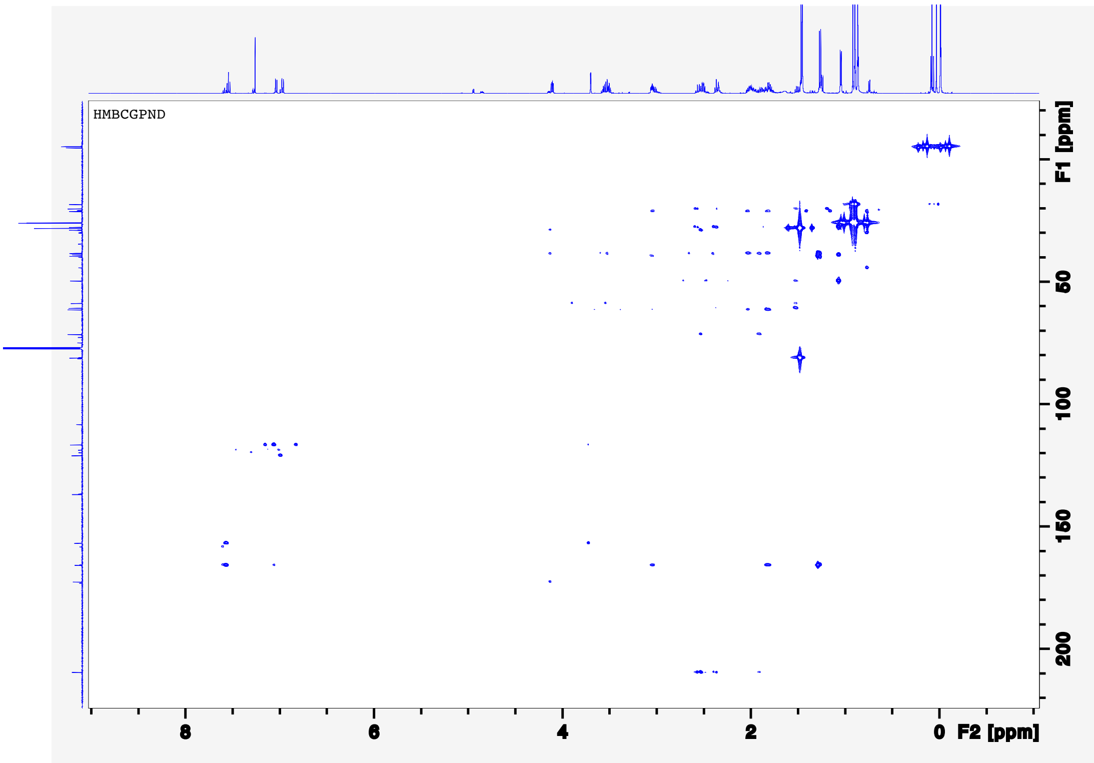

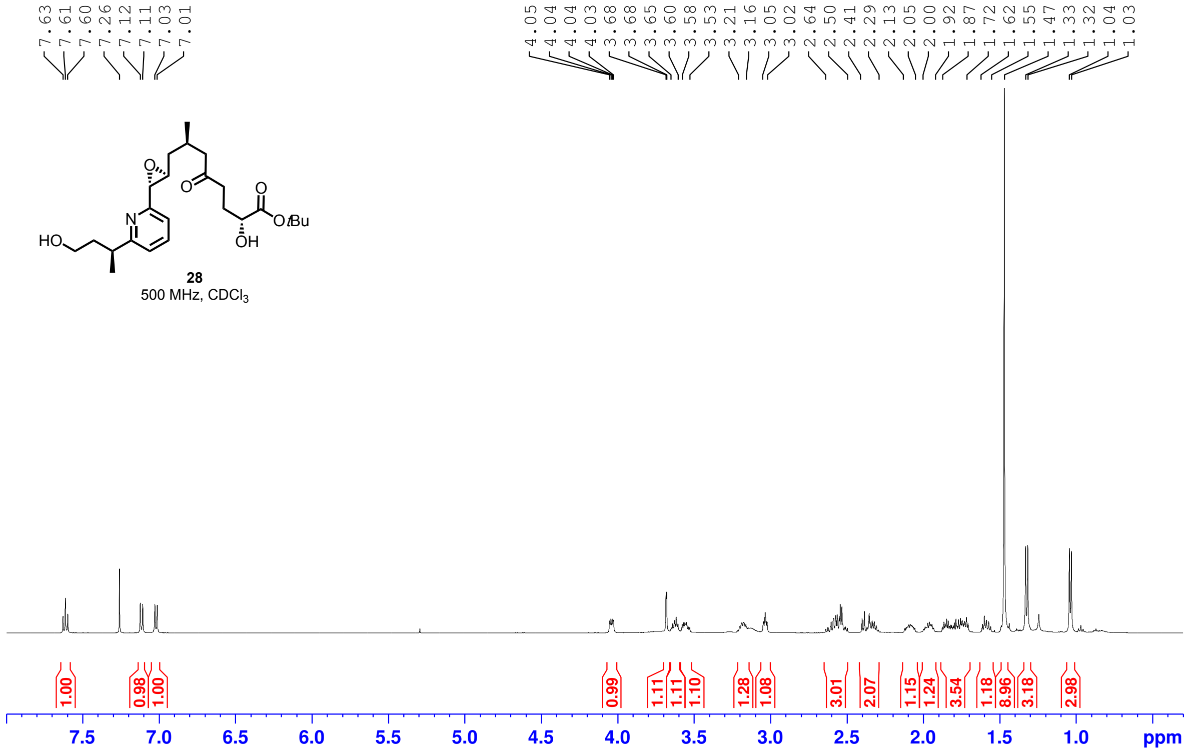

28
500 MHz, $CDCl_3$

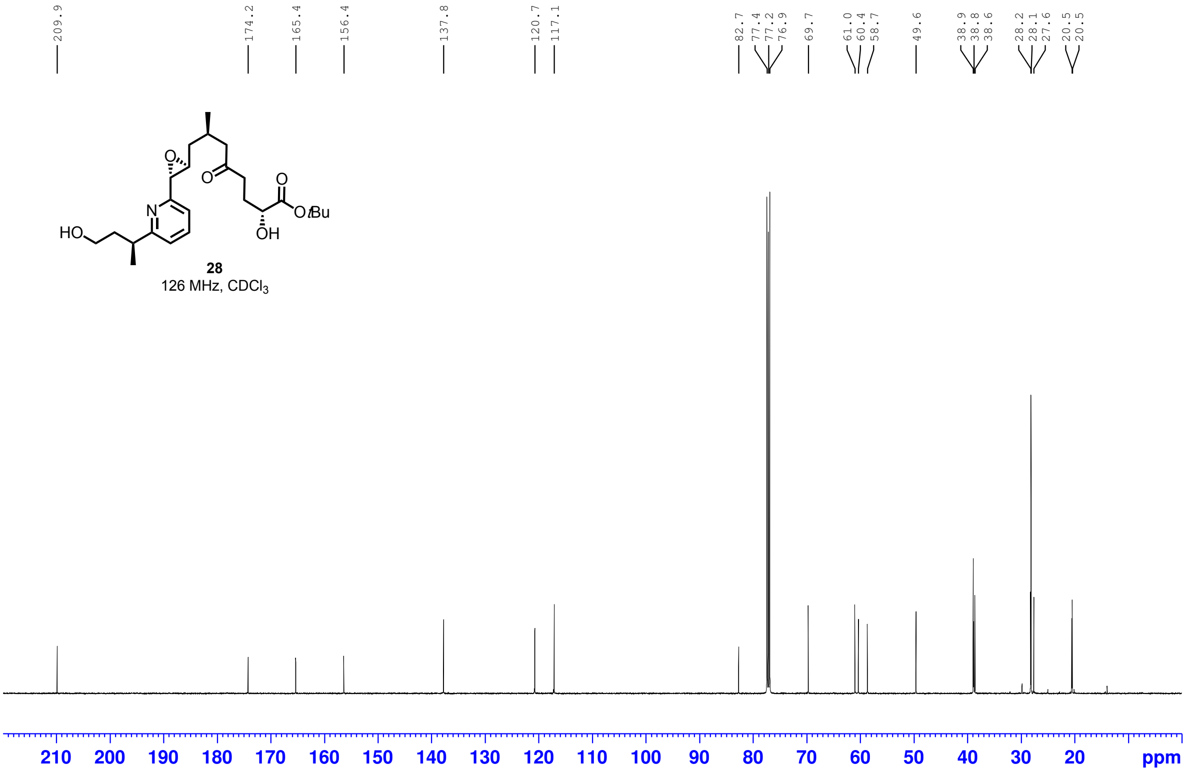

28
126 MHz, $CDCl_3$

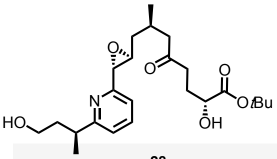

28
500/126 MHz, $CDCl_3$

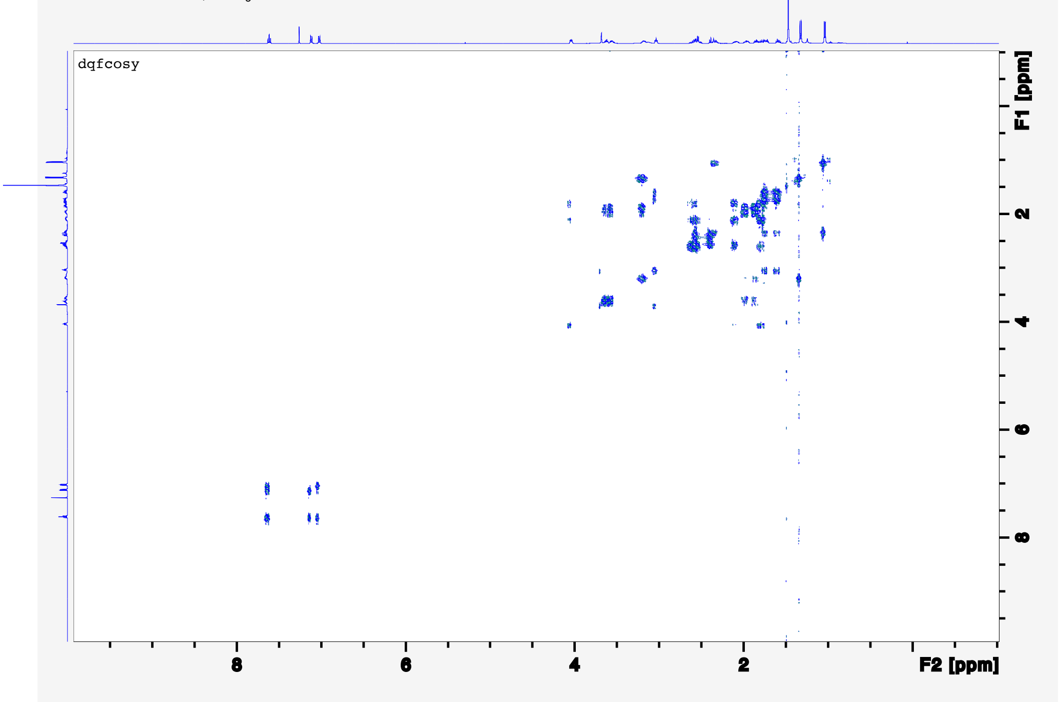

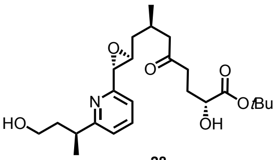

28
500/126 MHz, $CDCl_3$

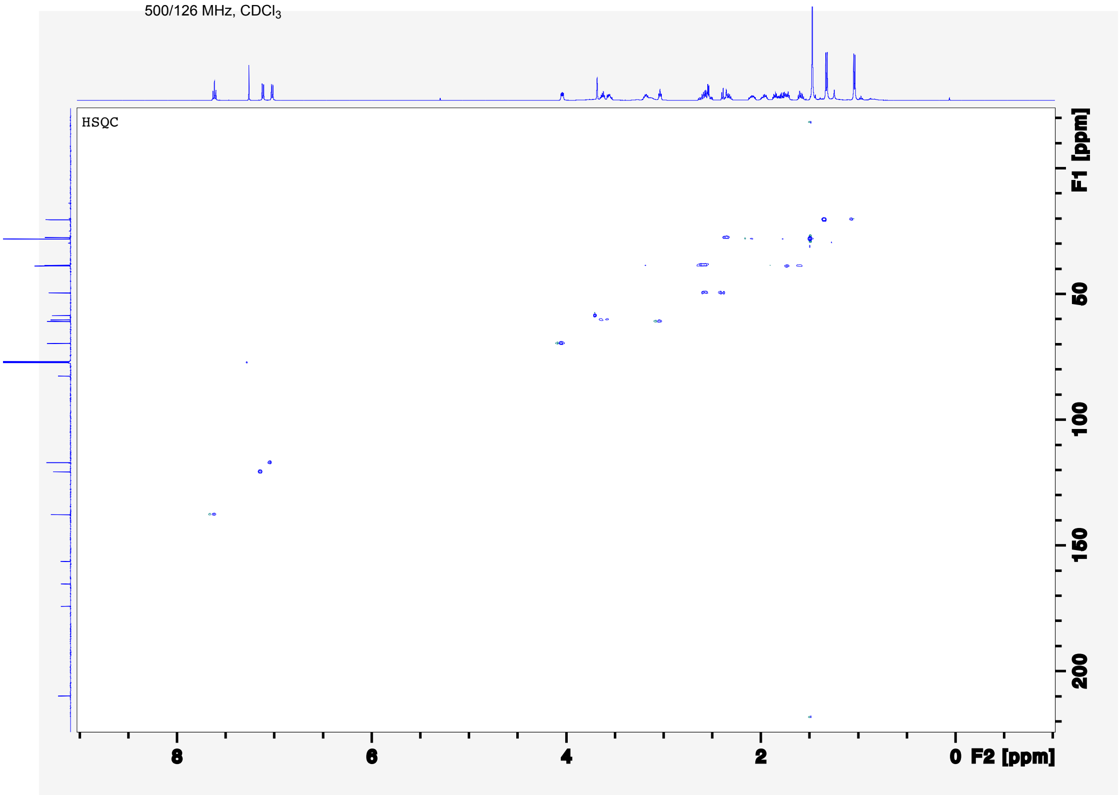

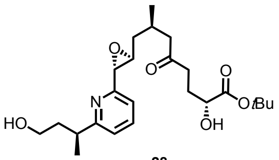

28
500/126 MHz, $CDCl_3$

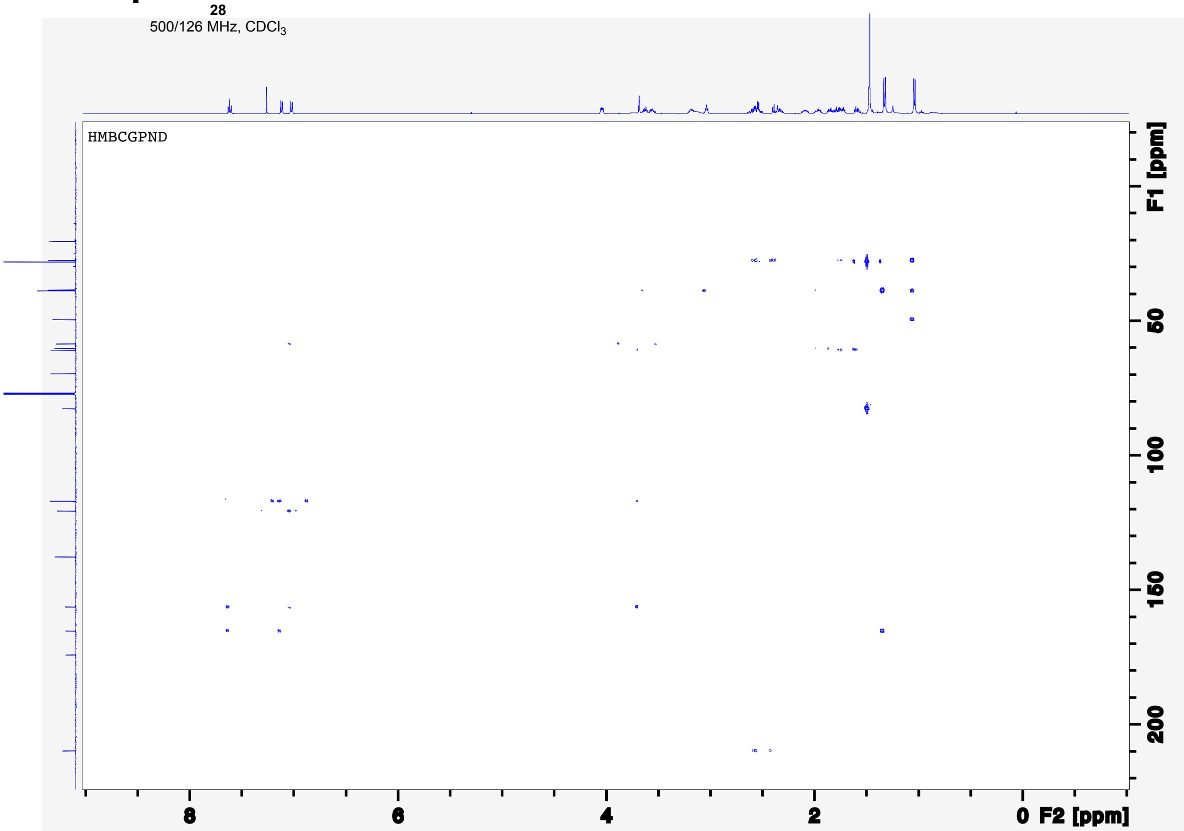

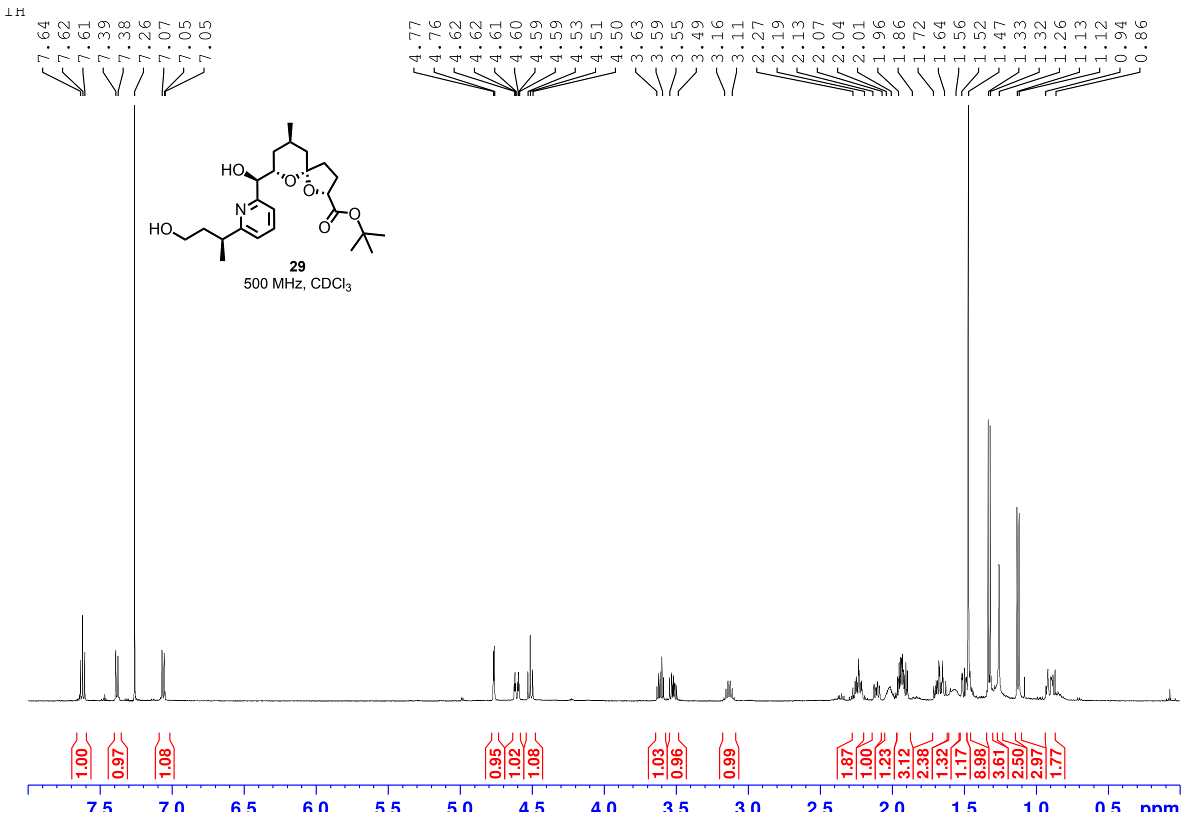

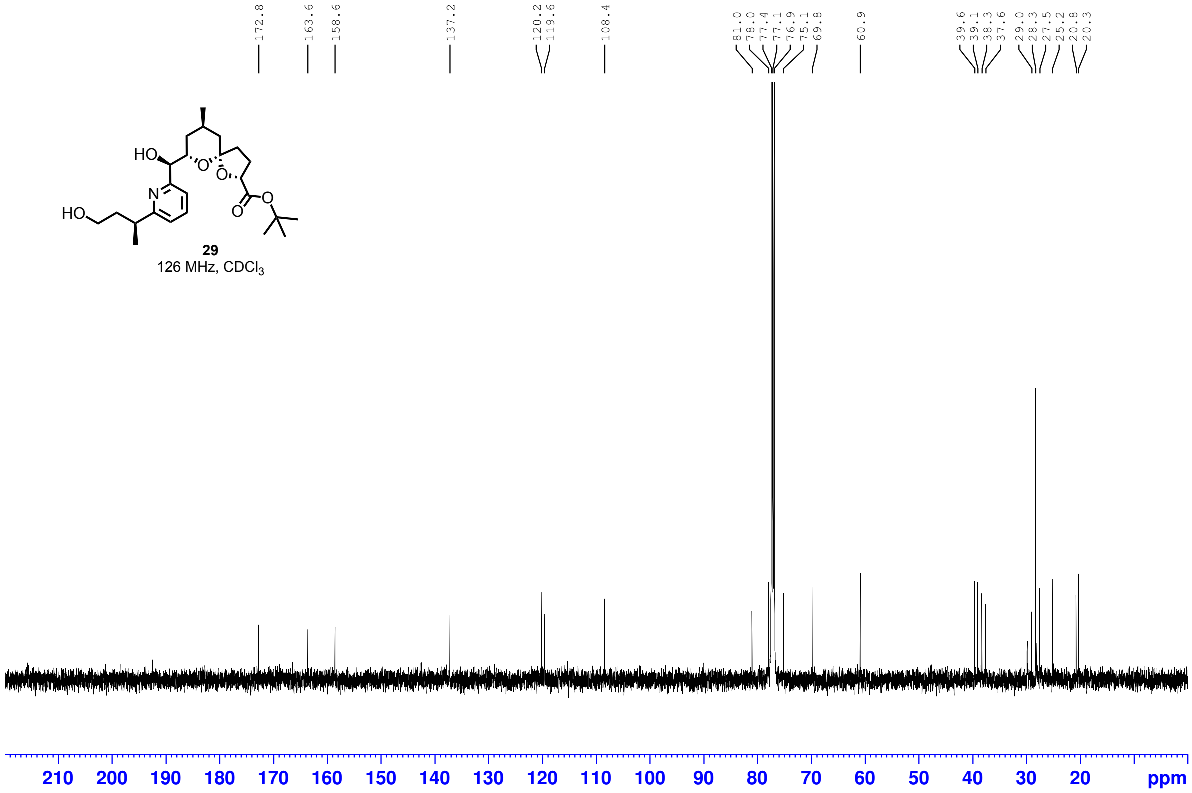

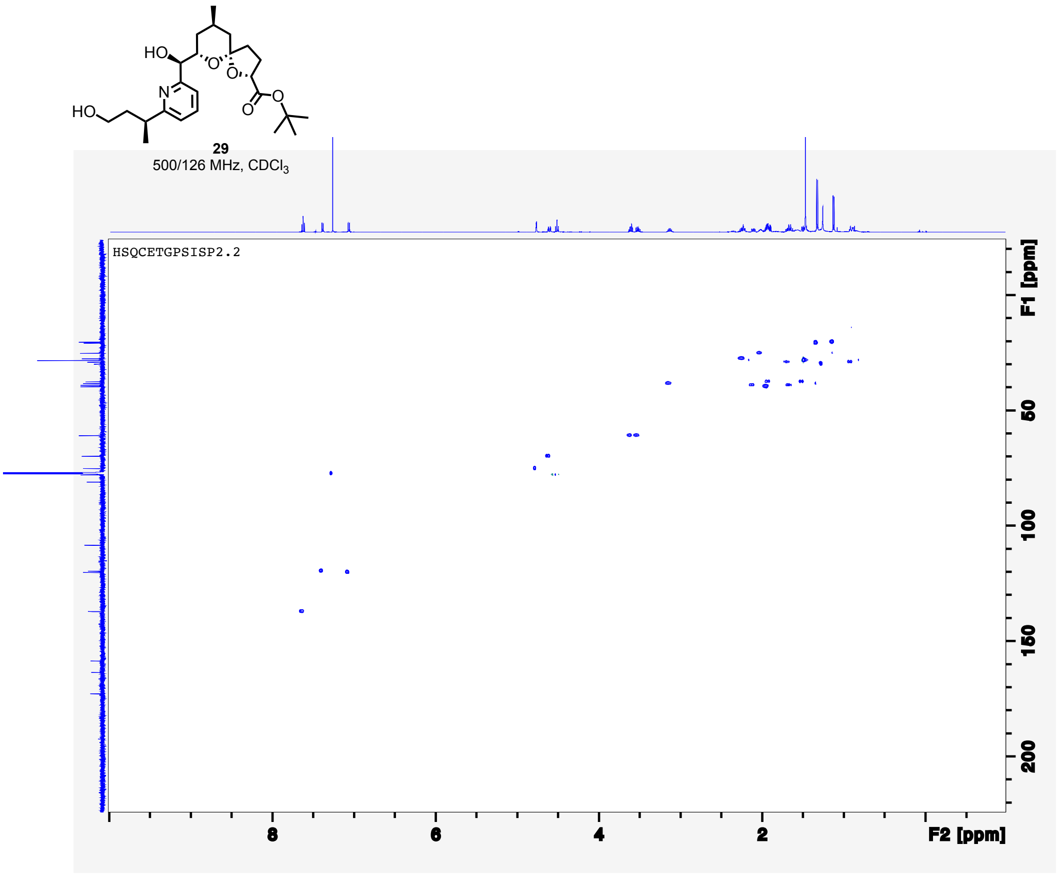
HO
N
HO
O
O
O
O
29
500/126 MHz, CDCl3
HSQCETGPSISP2.2
F1 [ppm]
50
100
150
200
8
6
4
2
F2 [ppm]

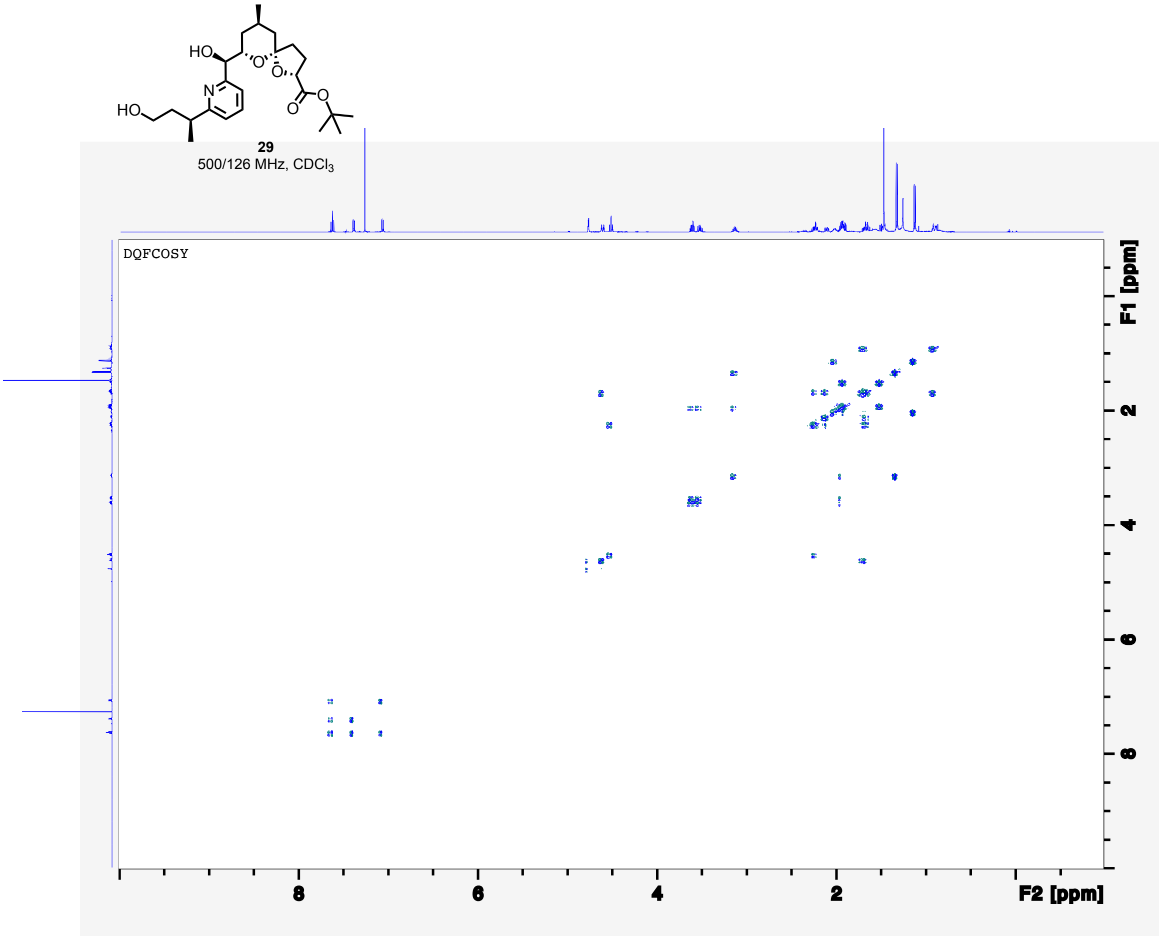
HO
N
HO
O
O
O
O
29
500/126 MHz, CDCl3
DQFCOSY
F1 [ppm]
2
4
6
8
F2 [ppm]

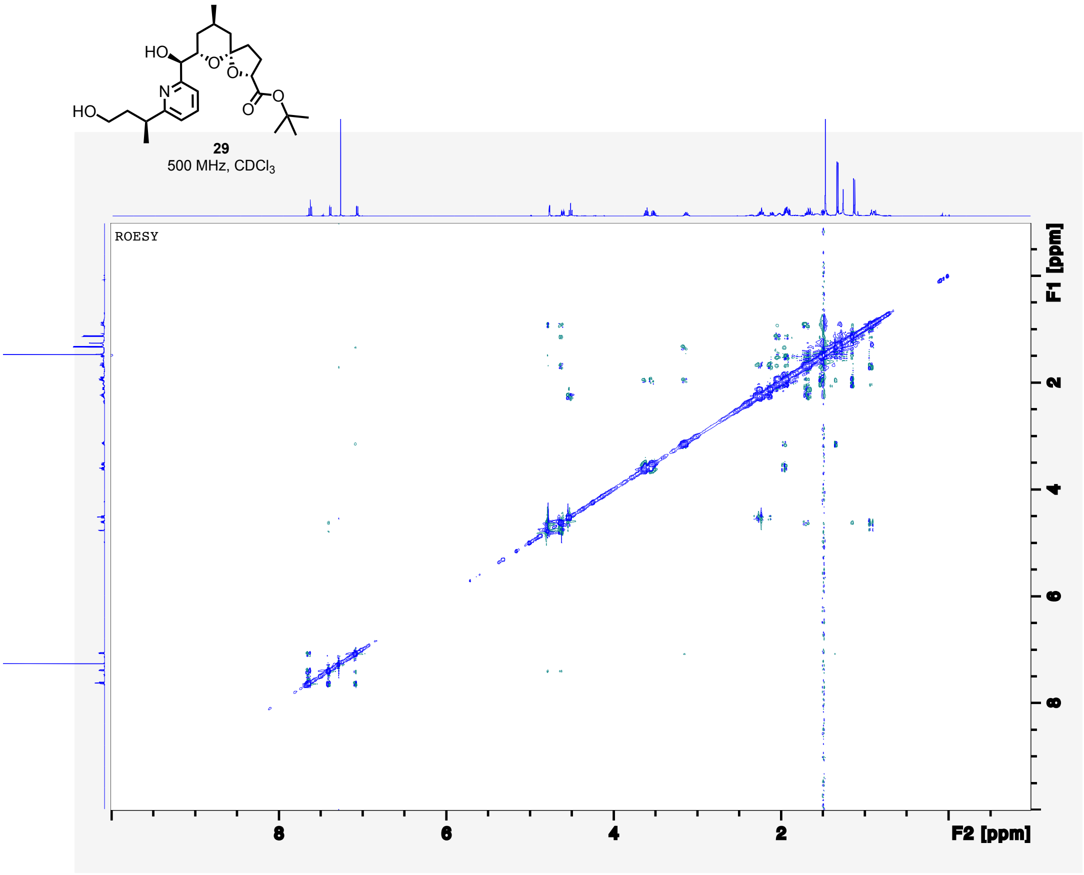

29
500 MHz, $CDCl_3$

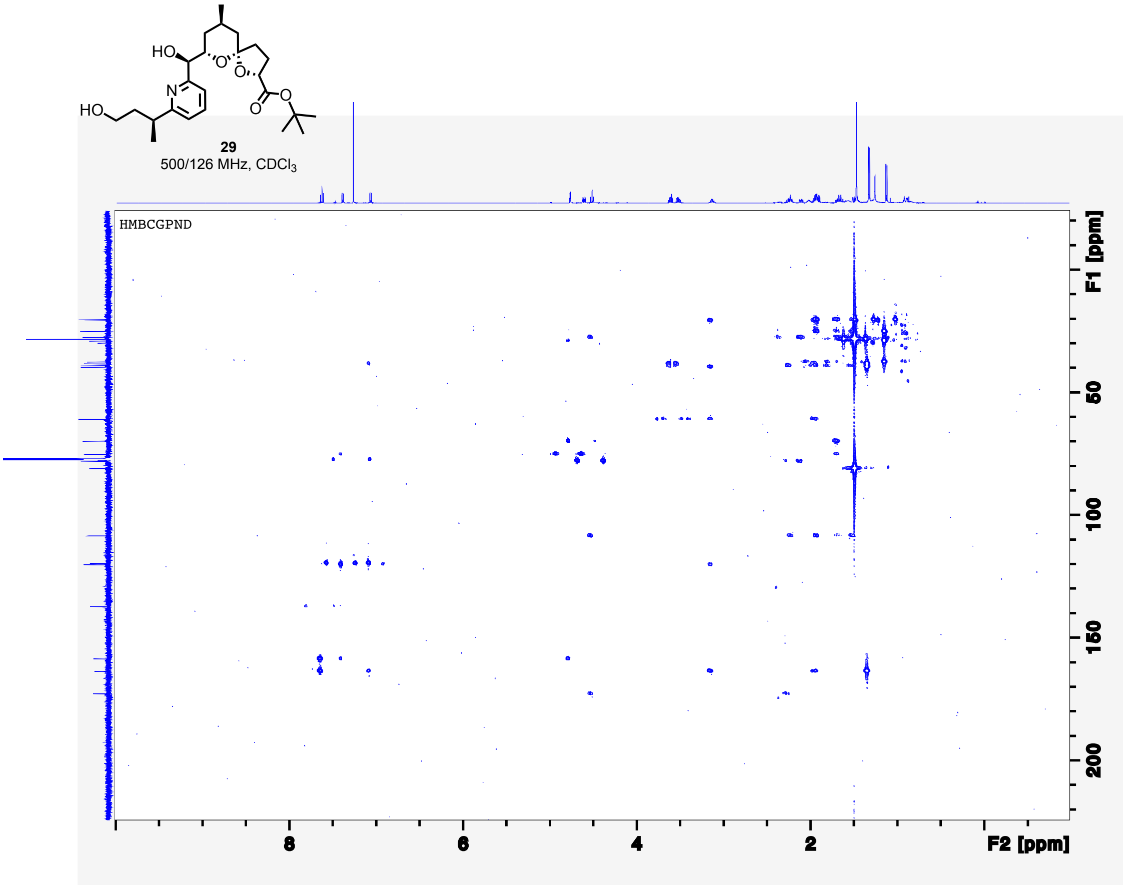

29
500/126 MHz, $CDCl_3$

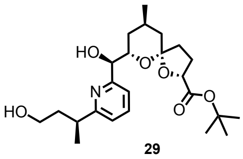

29
500 MHz, CDCl$_3$

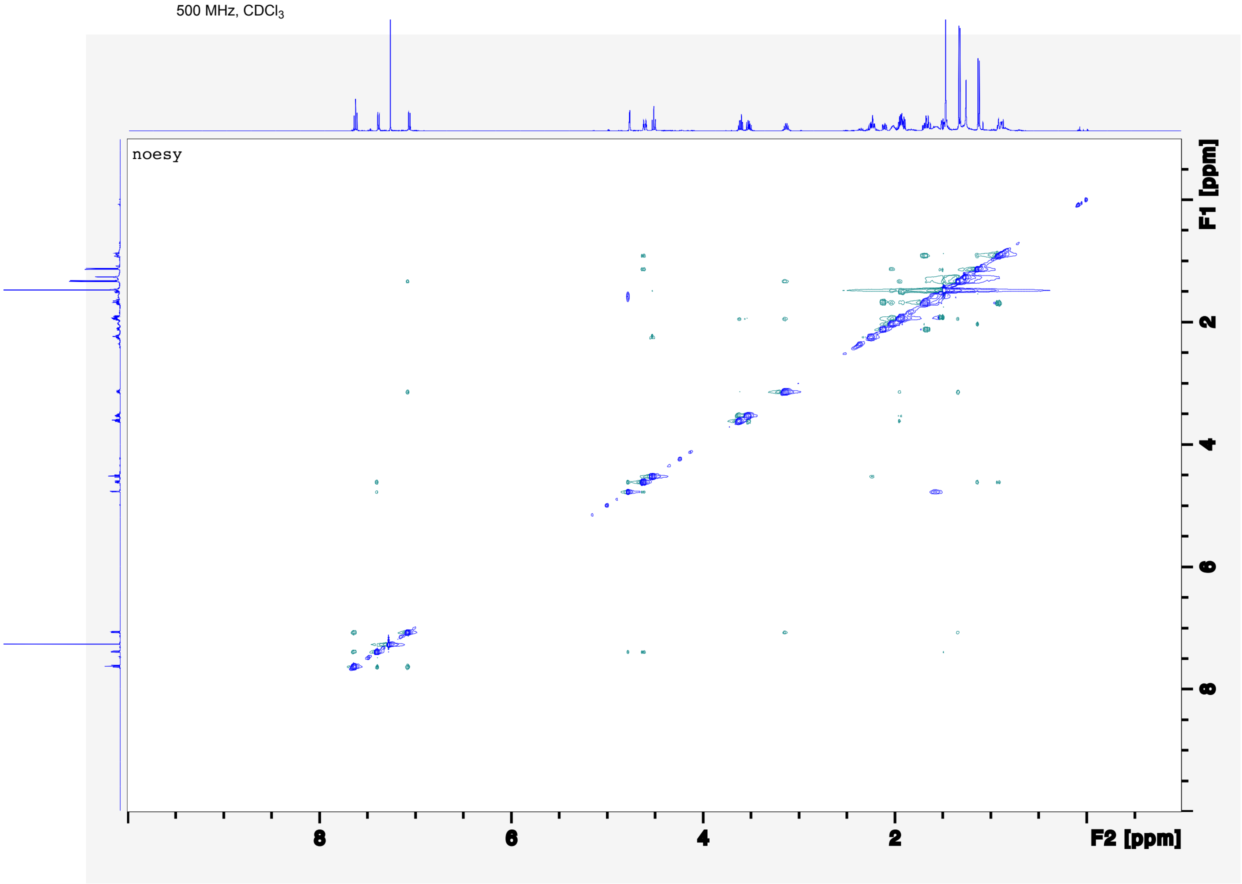

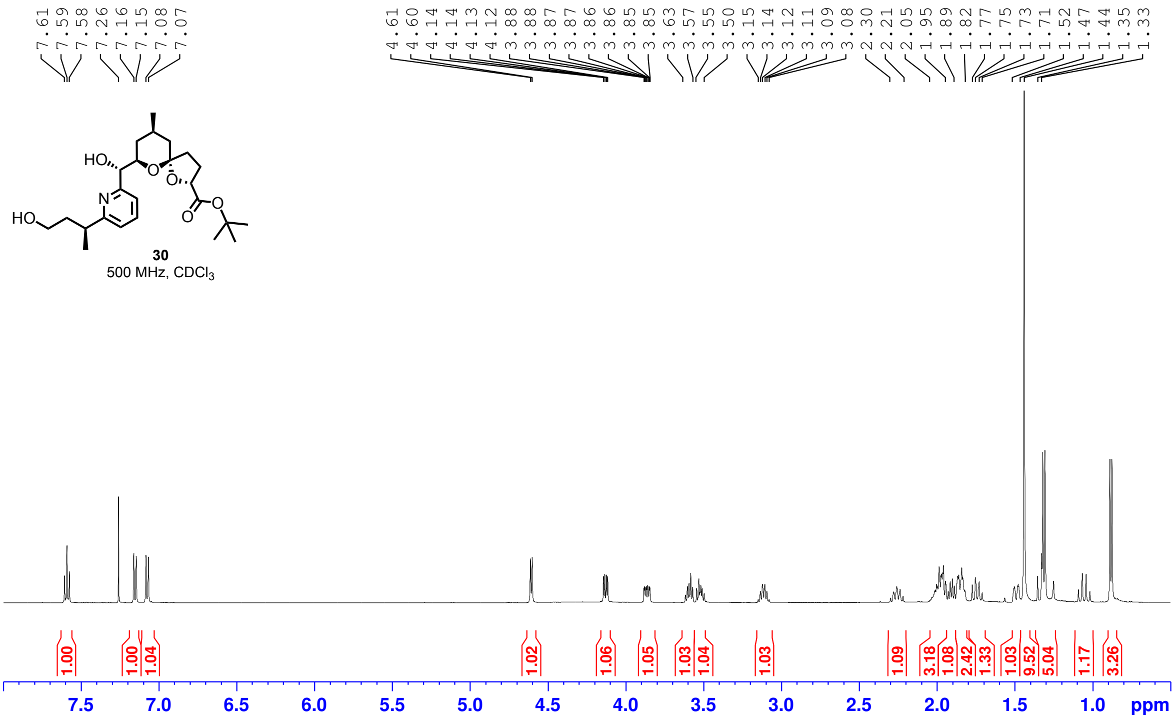
7.61 7.59 7.58 7.26 7.16 7.15 7.08 7.07
4.61 4.60 4.14 4.14 4.13 4.12 3.88 3.88 3.87 3.87 3.86 3.86 3.85 3.85 3.63 3.57 3.55 3.50 3.15 3.14 3.12 3.11 3.09 3.08 2.30 2.21 2.05 1.95 1.89 1.82 1.77 1.75 1.73 1.71 1.52 1.47 1.44 1.35 1.33
HO
N
O
O
O
HO
30
500 MHz, CDCl3
1.00 1.00 1.04 1.02 1.06 1.05 1.03 1.04 1.03 1.09 3.18 1.08 2.42 1.33 1.03 9.52 5.04 1.17 3.26
7.5 7.0 6.5 6.0 5.5 5.0 4.5 4.0 3.5 3.0 2.5 2.0 1.5 1.0 ppm

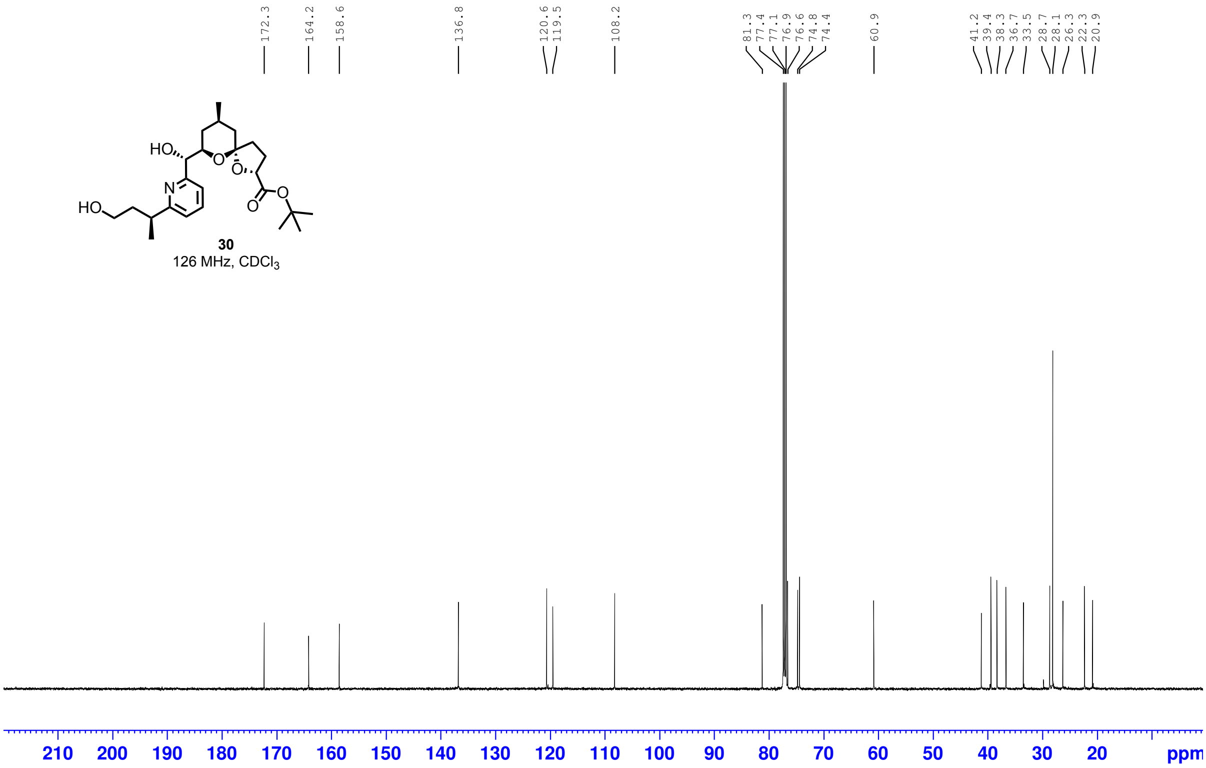
172.3 164.2 158.6 136.8 120.6 119.5 108.2 81.3 77.4 77.1 76.9 76.6 74.8 74.4 60.9 41.2 39.4 38.3 36.7 33.5 28.7 28.1 26.3 22.3 20.9
HO
N
O
O
O
HO
30
126 MHz, CDCl3
210 200 190 180 170 160 150 140 130 120 110 100 90 80 70 60 50 40 30 20 ppm

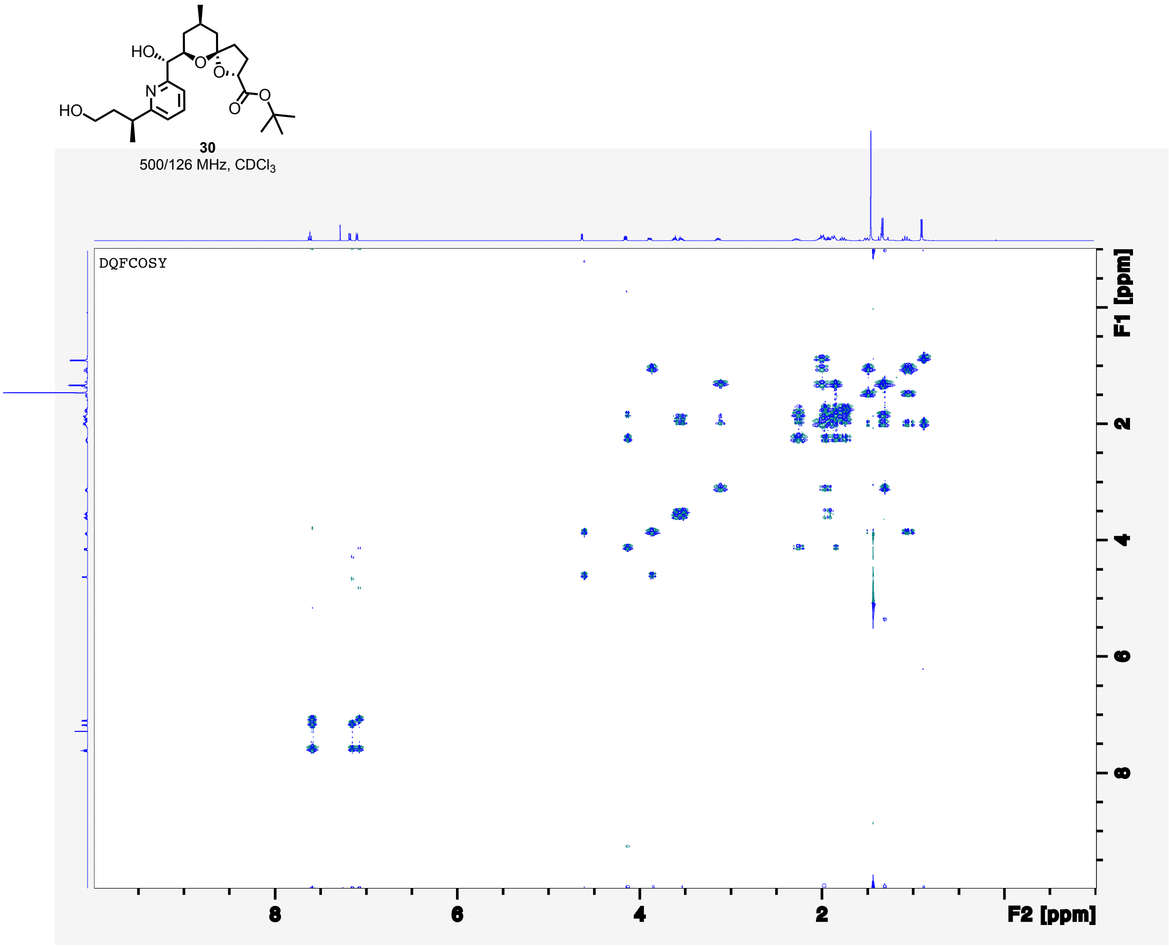
HO
HO
N
O
O
O
O
30
500/126 MHz, CDCl3
DQFCOSY
F1 [ppm]
2
4
6
8
8
6
4
2
F2 [ppm]

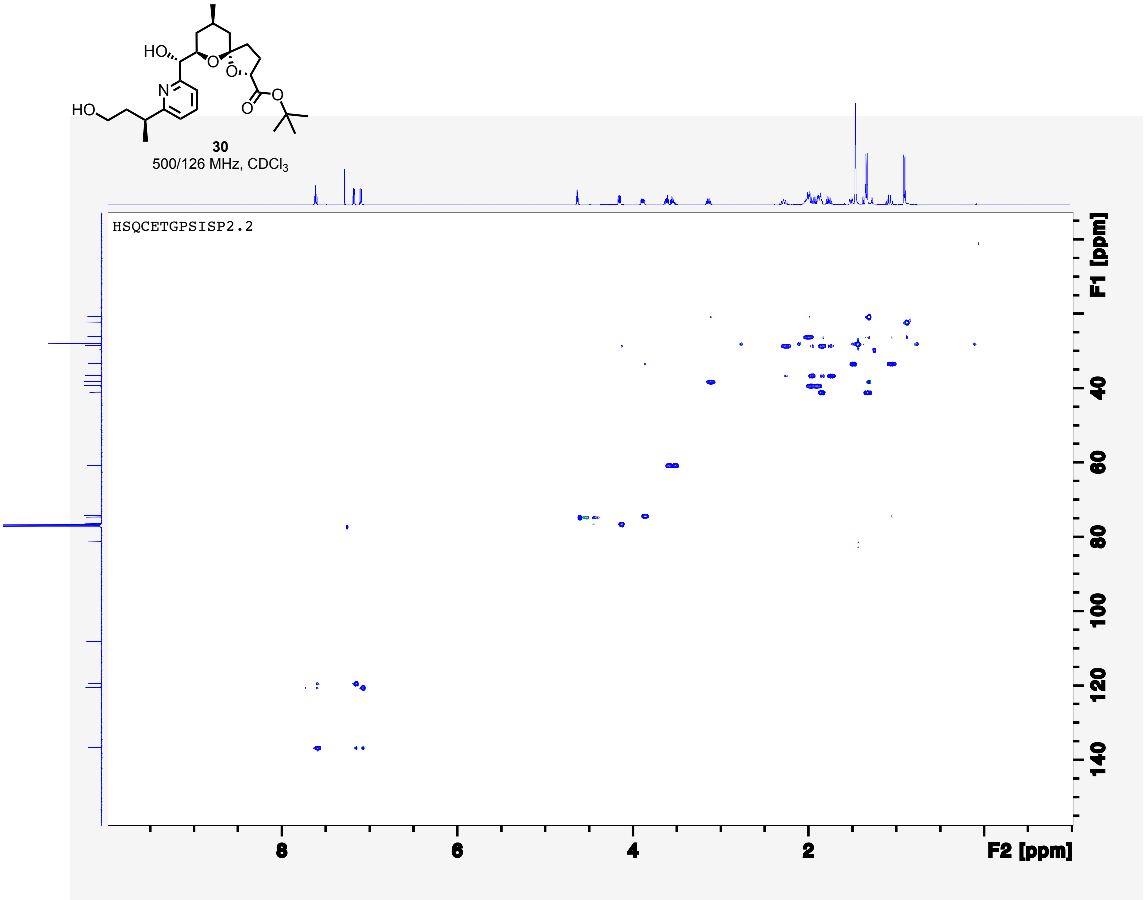
HO
HO
N
O
O
O
O
30
500/126 MHz, CDCl3
HSQCETGPSISP2.2
F1 [ppm]
40
60
80
100
120
140
8
6
4
2
F2 [ppm]

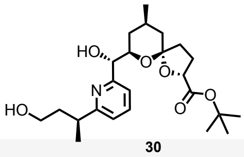

30

500/126 MHz, $CDCl_3$

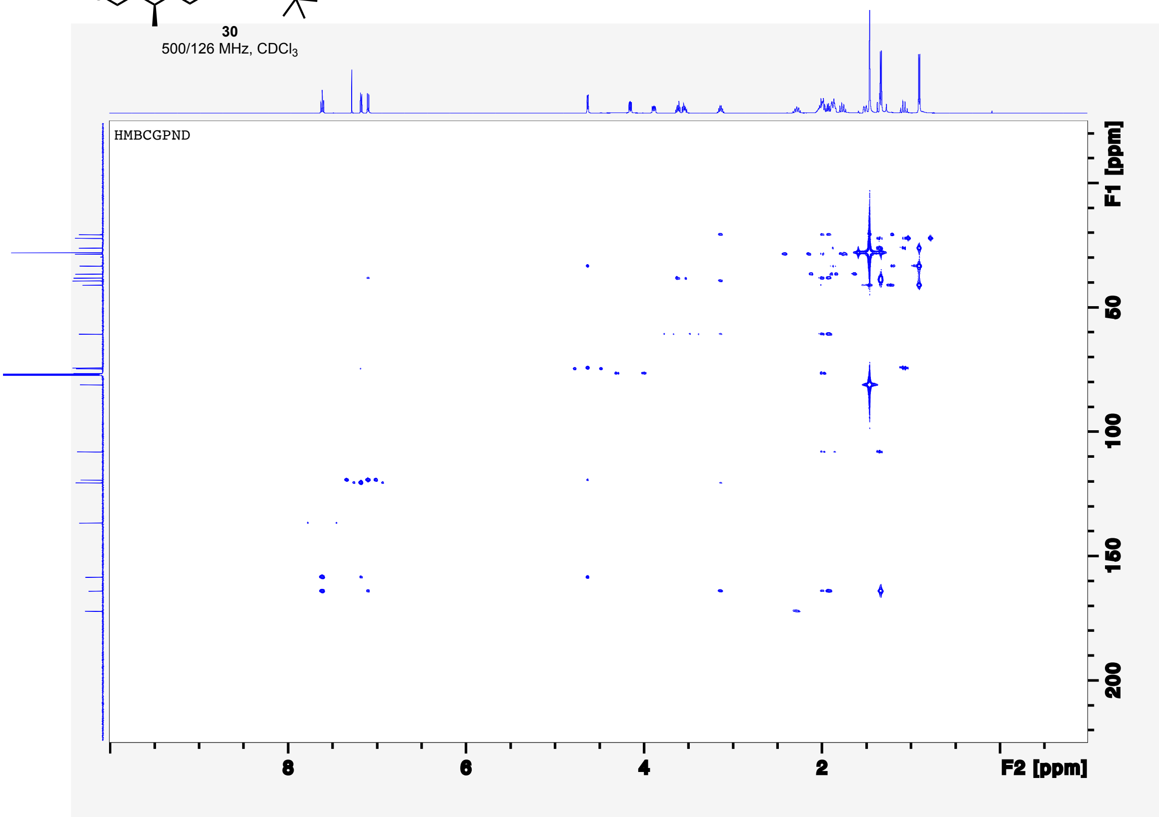

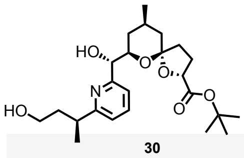

30

500 MHz, $CDCl_3$

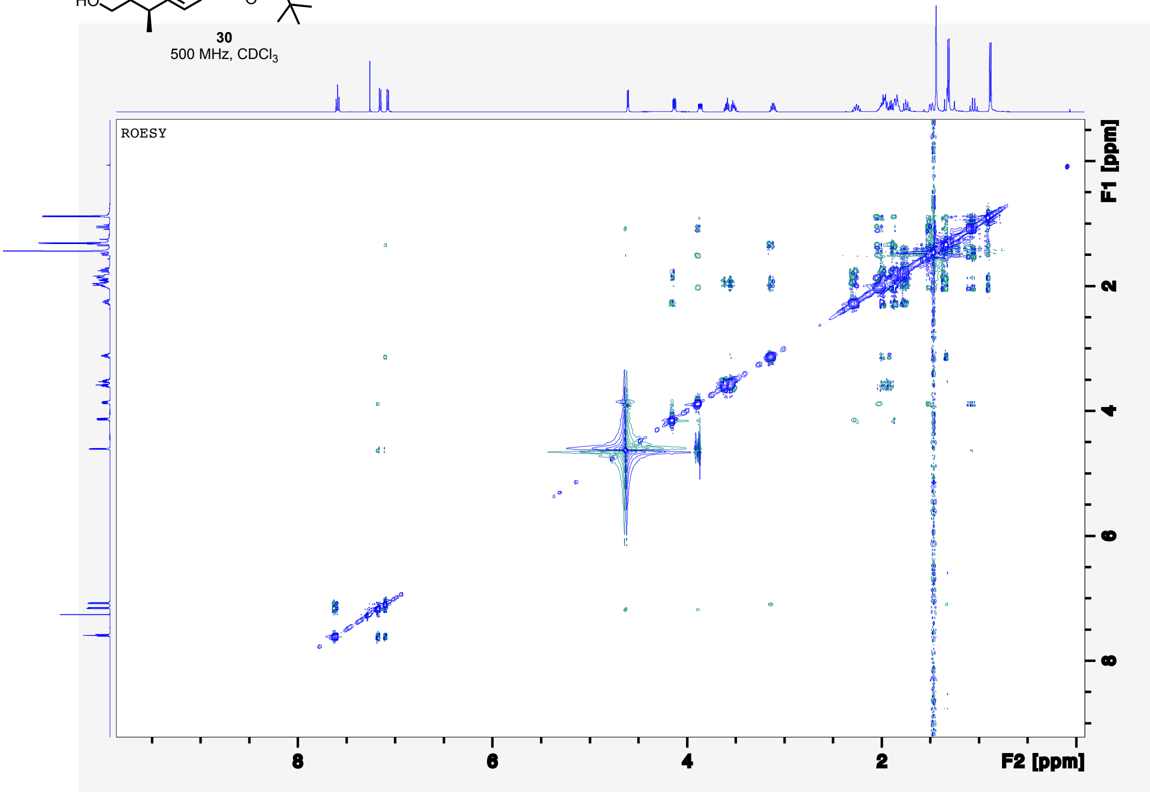

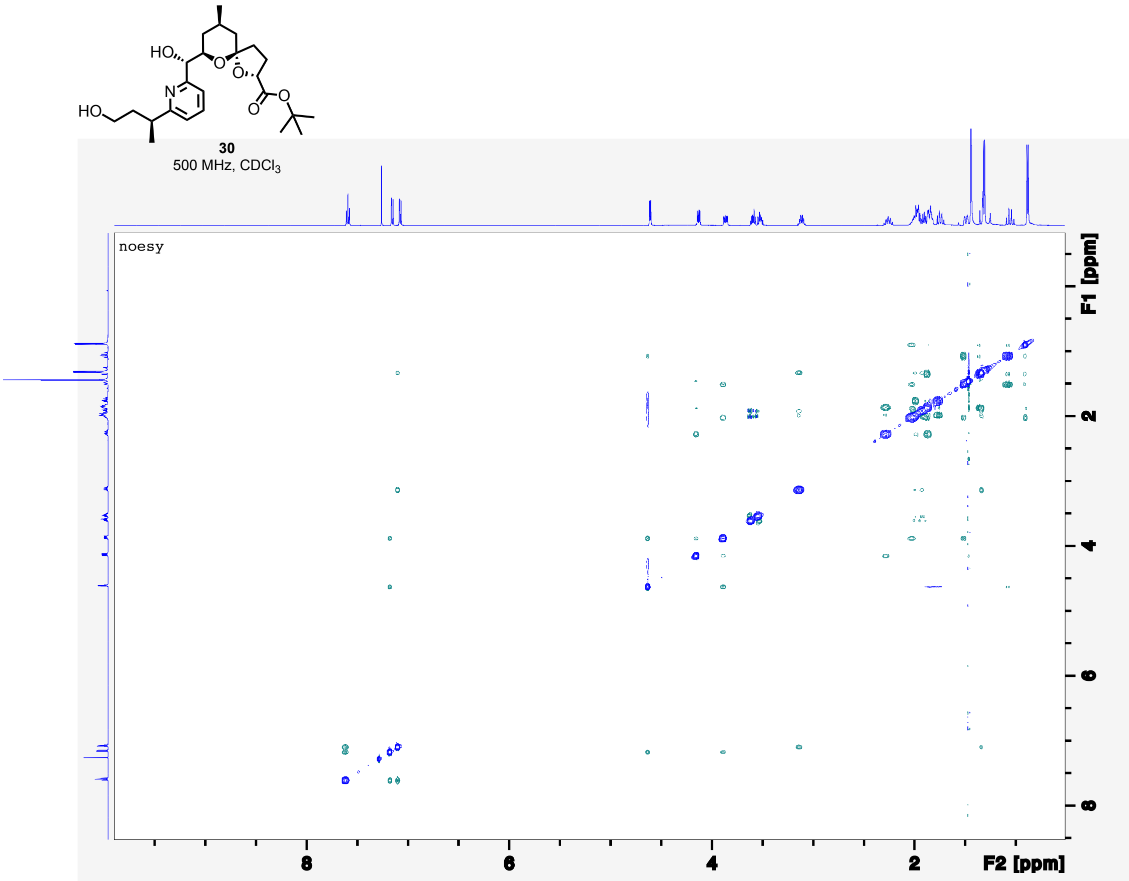
HO
HO
N
O
O
O
O
30
500 MHz, CDCl3
noesy
F1 [ppm]
2
4
6
8
F2 [ppm]
8
6
4
2

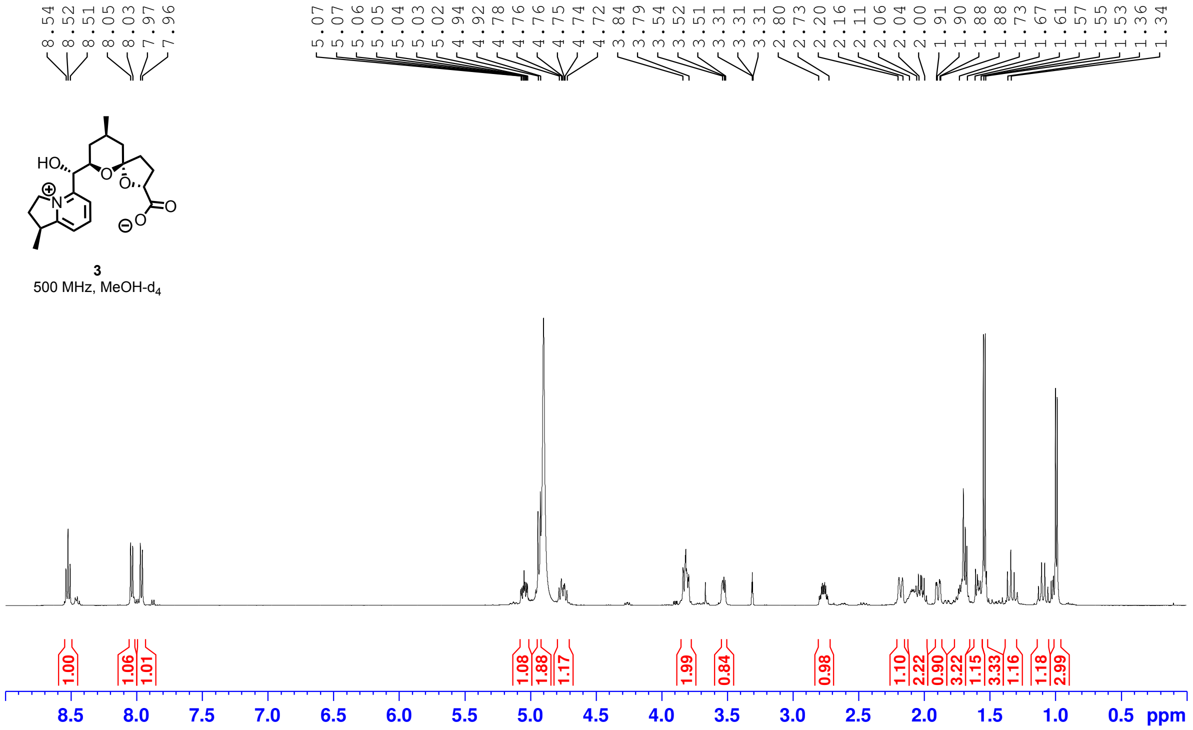

3
500 MHz, MeOH-d_4

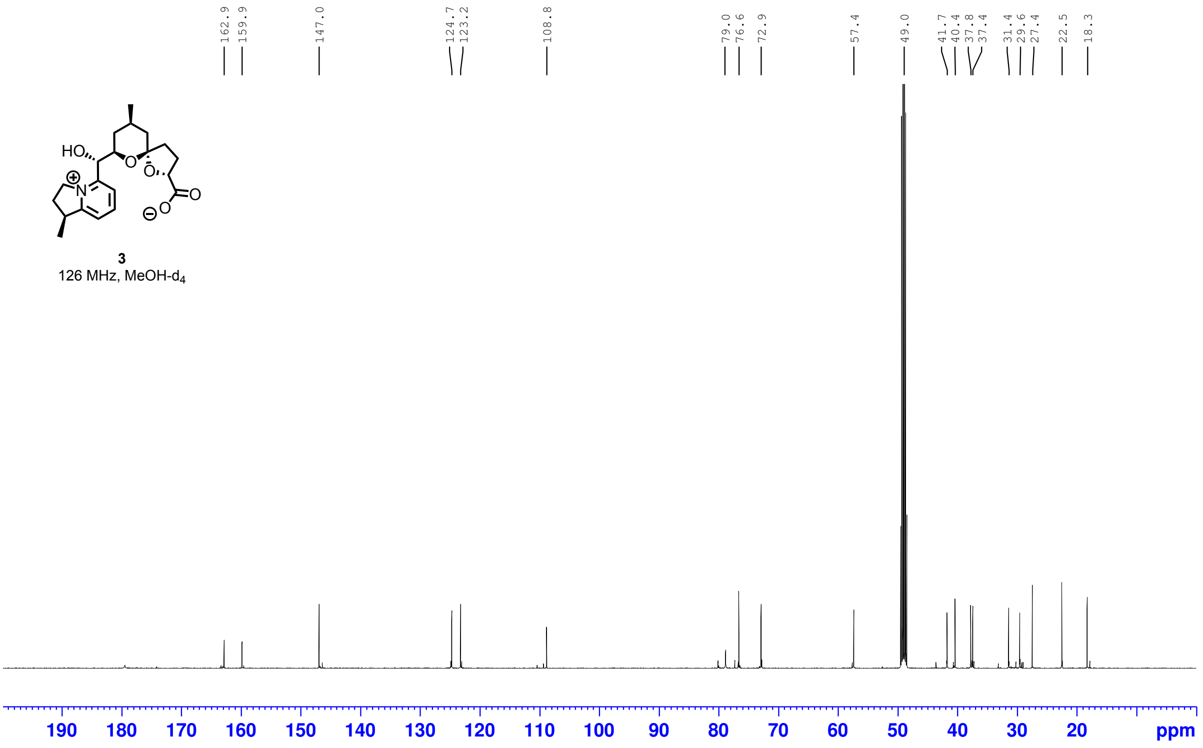

3
126 MHz, MeOH-d_4

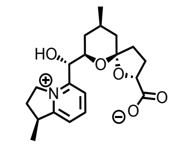

3

500/126 MHz, MeOH-d4

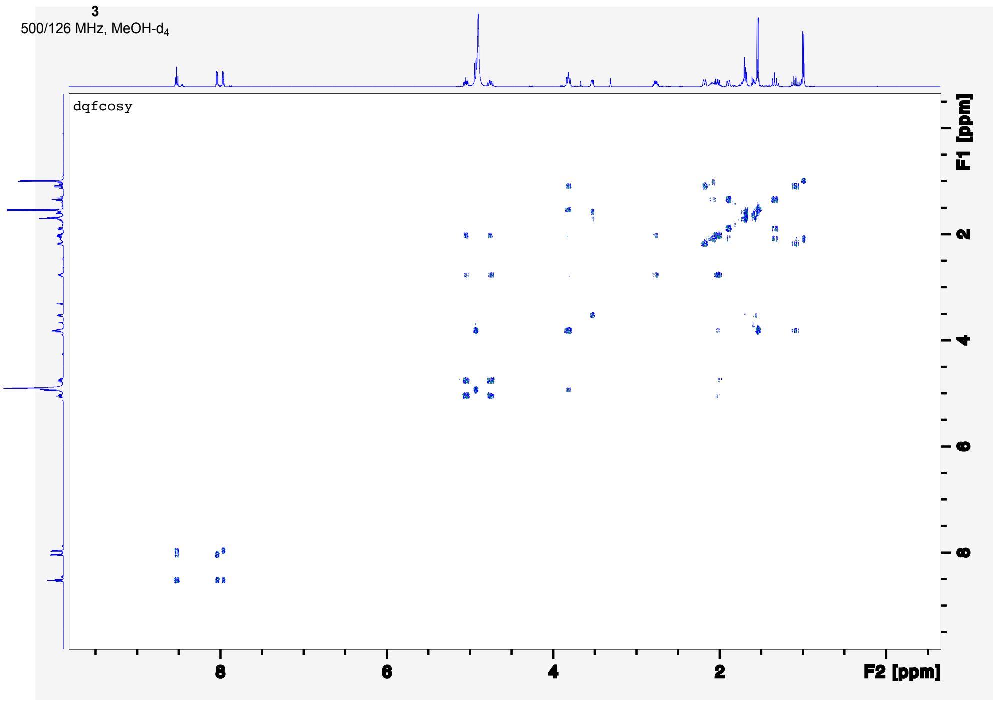

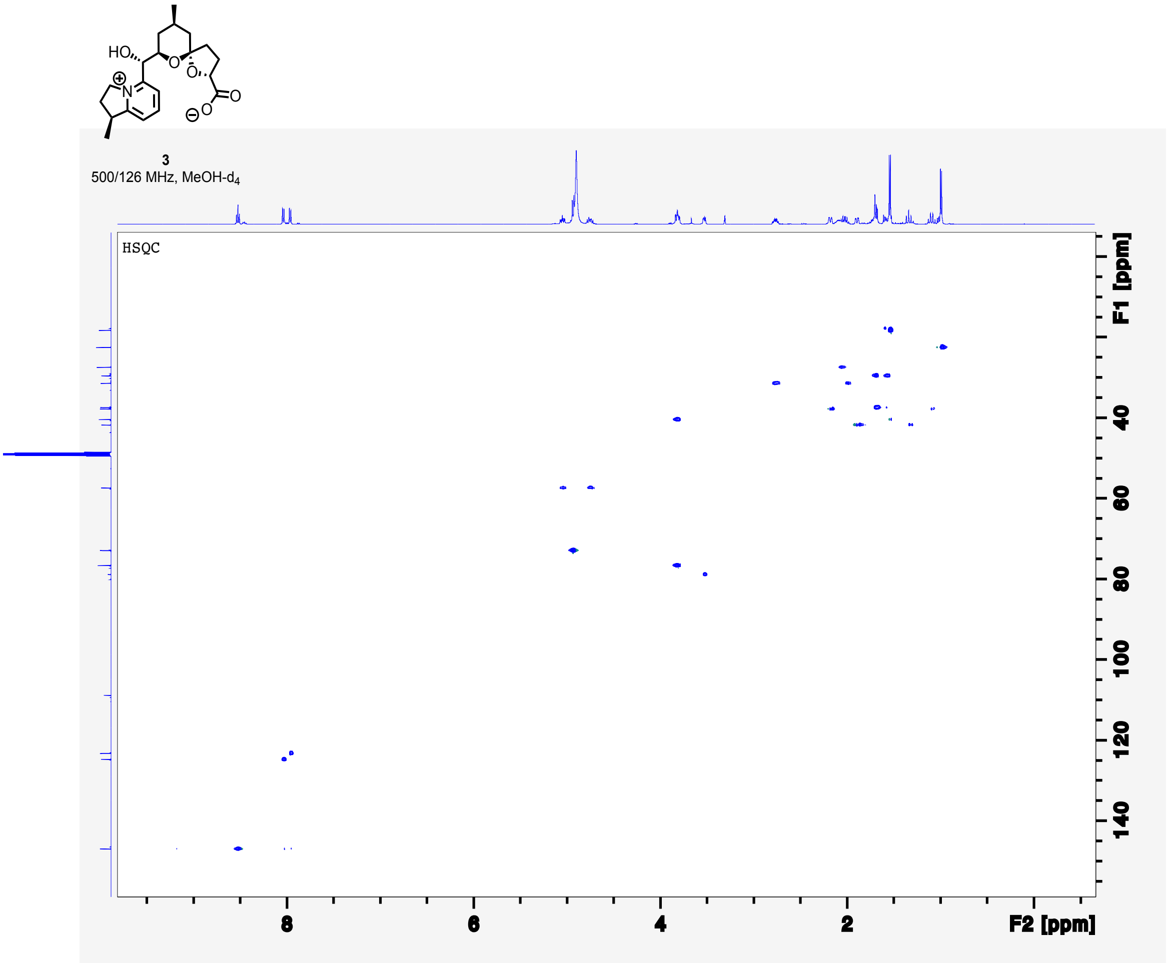
HO
N
O
O
O
O
3
500/126 MHz, MeOH-d4
HSQC
F1 [ppm]
40
60
80
100
120
140
8
6
4
2
F2 [ppm]

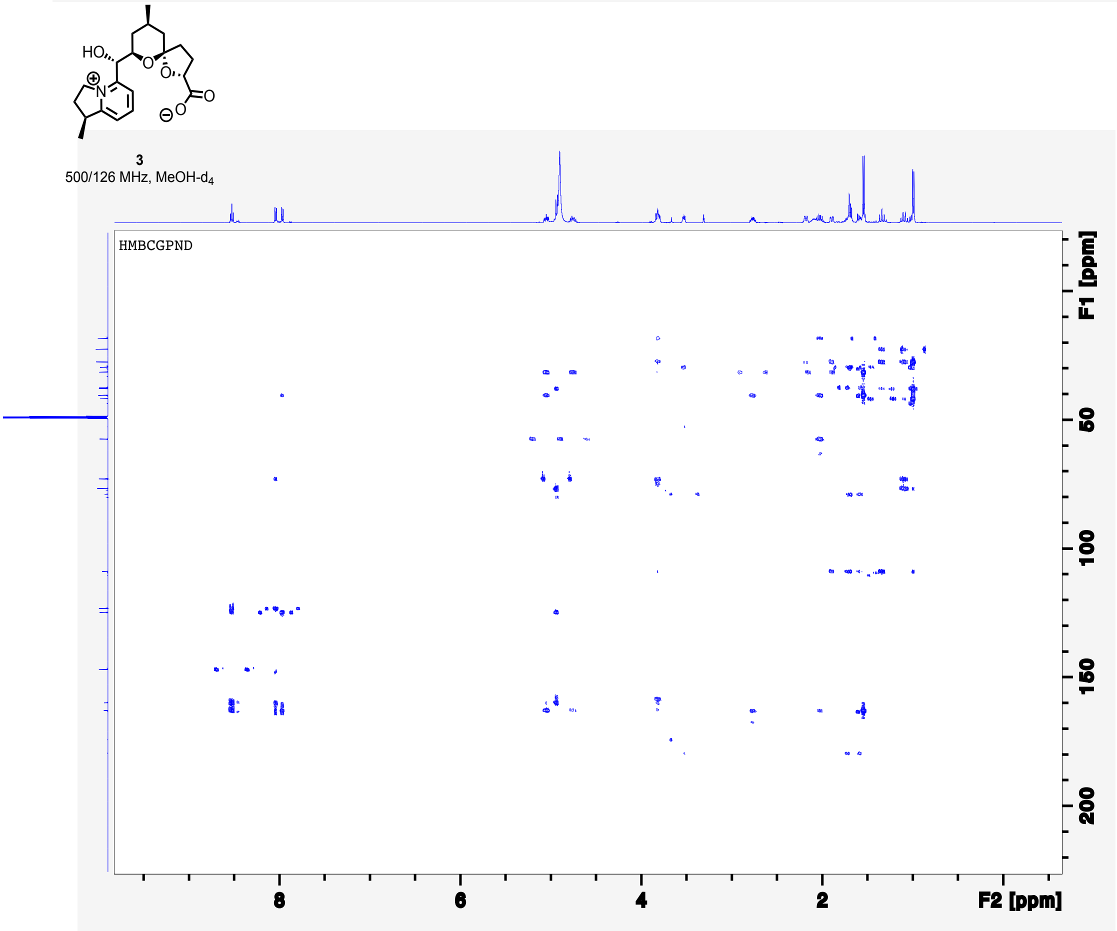
HO
N
O
O
O
O
3
500/126 MHz, MeOH-d4
HMBCGPND
F1 [ppm]
50
100
150
200
8
6
4
2
F2 [ppm]

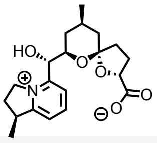

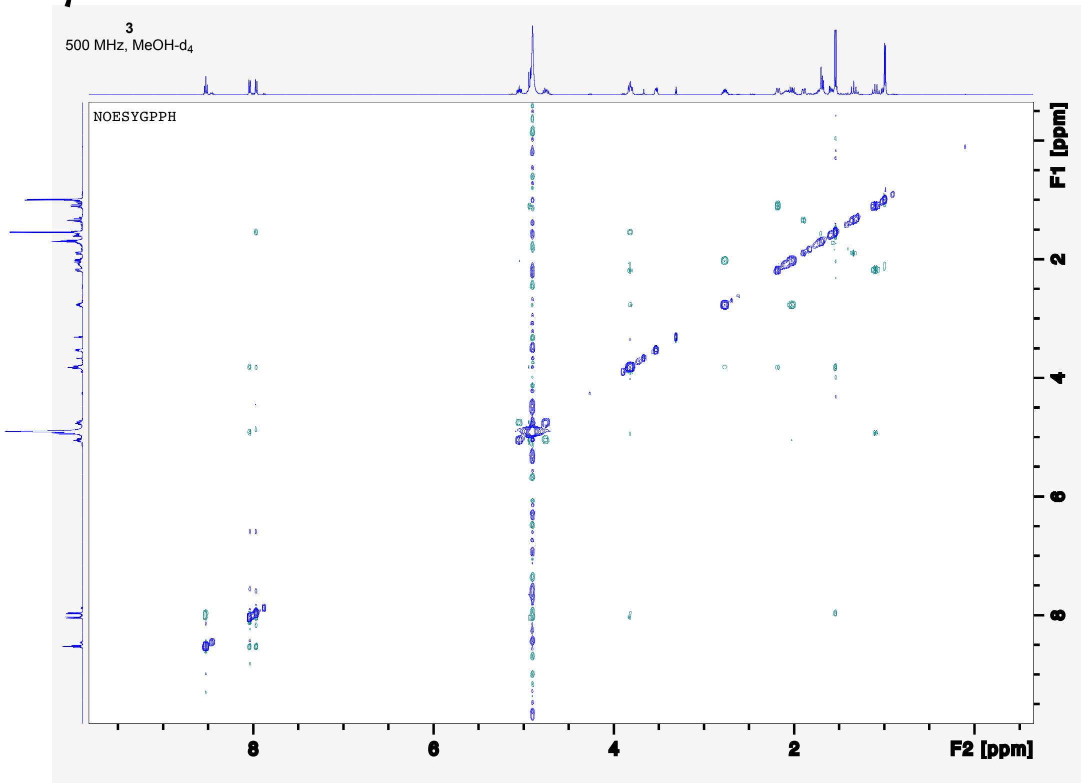
3
500 MHz, MeOH-d4
NOESYGPPH
F1 [ppm]
F2 [ppm]
8
6
4
2

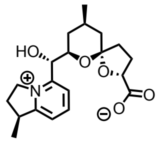

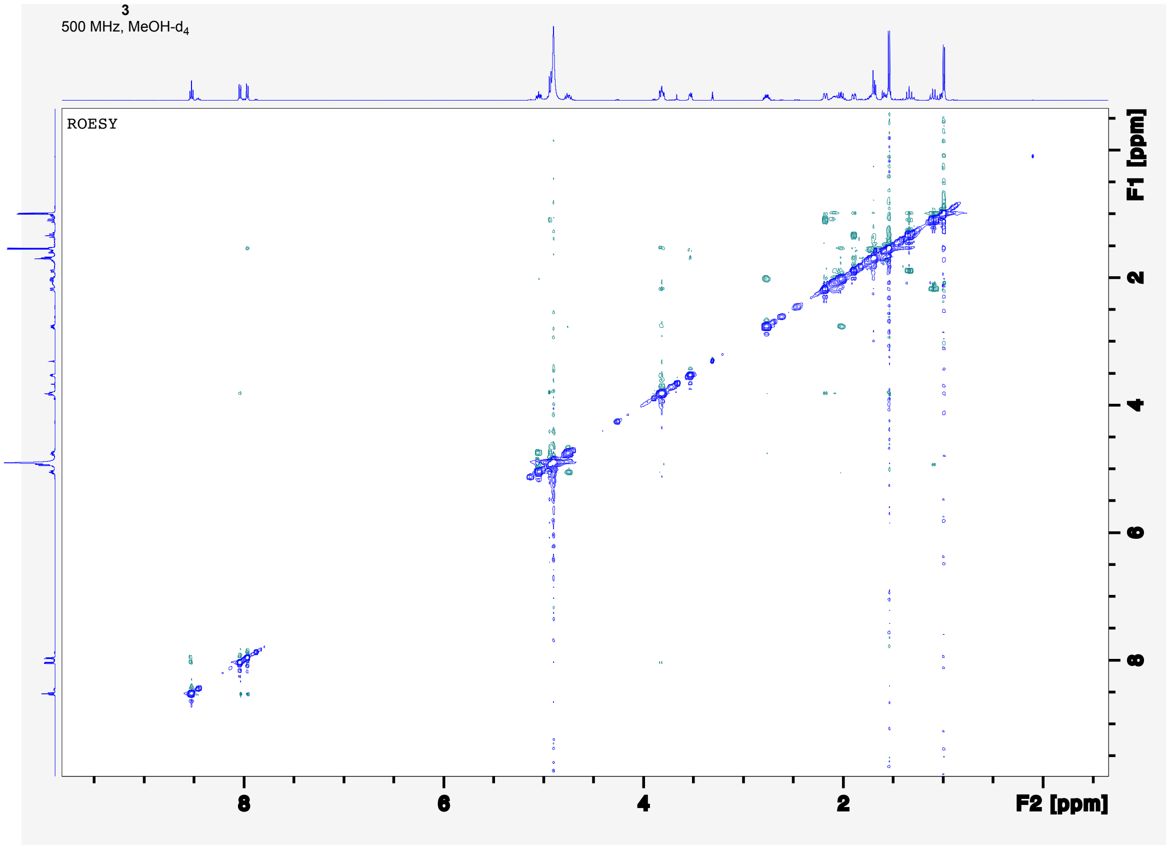
3
500 MHz, MeOH-d4
ROESY
F1 [ppm]
F2 [ppm]
8
6
4
2

4 Establishment of the C3 Configuration

4.1 Experimental procedures & Characterization Data

A) Attempts in stereoretentive cross coupling

2-(4-((*tert*-butyldimethylsilyl)oxy)butan-2-yl)-6-vinylpyridine (10)

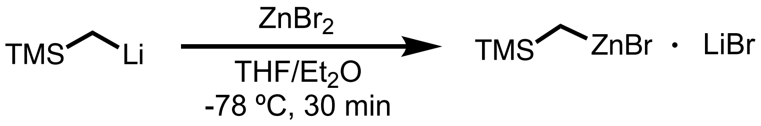

In a flame-dried flask $ZnBr_2$ (3.00 g, 11.76 mmol, 1.00 eq.) was dried at 300 °C for 30 min under vacuum. After addition of THF (15 mL) it was cooled to 0 °C. Me_3SiCH_2Li (0.4 M in *n*-pentane, 29.39 mL, 11.76 mmol, 1.00 eq.) was added dropwise and stirred for 1 hat rt. The solvent was removed under reduced pressure under argon atmosphere at 0 °C and Et_2O (3 mL) was added. This process was repeated three times and the residue was redissolved in Et_2O (10 mL) to obtain an ether solution of $Me_3SiCH_2ZnBr·LiBr$ (0.79 M). The concentration was determined by titration of a small aliquot with I_2.

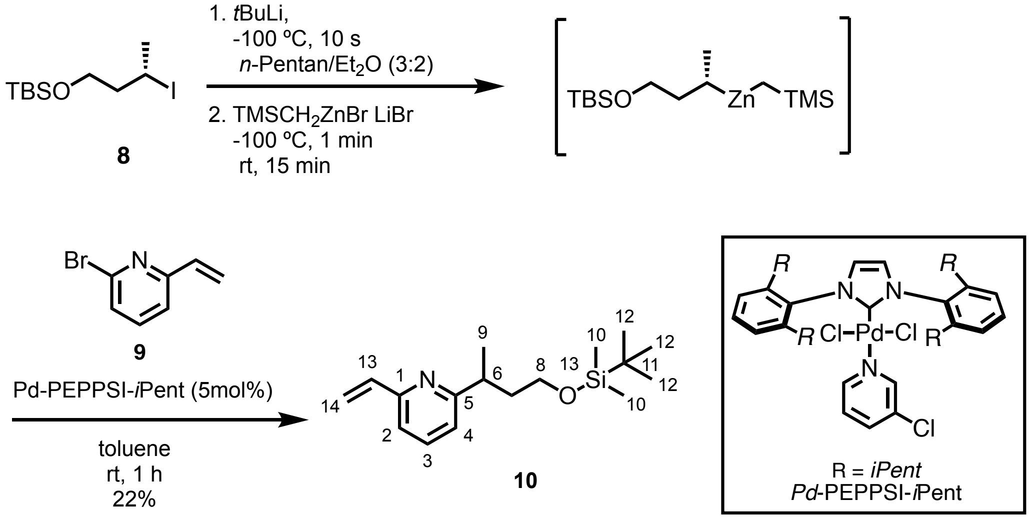

In a flame dried flask a solution of *n*-pentane (1.2 mL) and Et_2O (0.8 mL) was cooled down to −100 °C. *t*-BuLi (1.9 M in *n*-pentane, 0.18 mL, 0.35 mmol, 2.20 eq.) was added dropwise over 10 s. A solution of (*S*)-Iodbutane **8** (50 mg, 0.156 mmol, 1.00 eq.) in Et_2O (0.5 mL) was added dropwise over one minute. Subsequently $Me_3SiCH_2ZnBr·LiBr$ (0.78 M in Et_2O, 0.214 mL, 0.477 mmol, 1.05 eq.) was added dropwise and stirred for 1 min at −100 °C. The flask was warmed up to rt and stirred for 15 min and added dropwise to a solution of Vinylpyridine **9** (87.8 mg, 0.477 mmol, 3.00 eq.) and Pd-PEPPSI-*i*Pent (6 mg, 0.008 mmol, 0.05 eq.) in toluene (1.5 mL). After completion of the reaction, saturated ammonium chloride was added, and the mixture was extracted with Et_2O (3x10 mL). The organic layers were combined, dried

over anhydrous $MgSO_4$, filtered and the solvent was removed under reduced pressure. The crude product was purified by flash column chromatography (*n*-pentane/EtOAc 15:1) to give vinylpyridine **10** (10.0 mg, 0.034 mmol, 22%) as a colorless oil.

The analytical data is in accordance with chapter 2.6 (S)-2-(4-((tert-butyldimethylsilyl)oxy)butan-2-yl)-6-vinylpyridine (**10**). The *ee* was determined at the stage of alcohol **10a** with chiral HPLC.

(*S*)-3-(6-vinylpyridin-2-yl)butan-1-ol (10a)

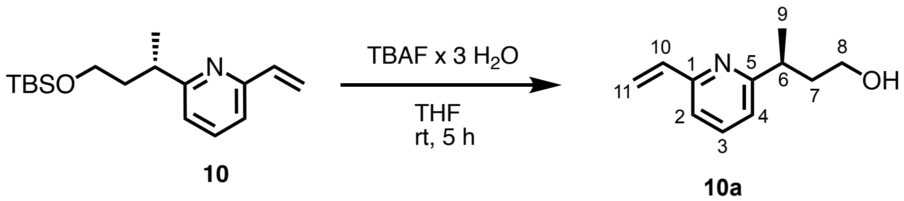

TBAF (275 mg, 0.872 mmol, 2.00 eq.) was added to a solution of vinylpyridine **10** (124 mg, 0.425 mmol, 1.00 eq.) in THF (4 mL) at 0°C and the mixture was stirred for 5 h. After completion of the reaction saturated ammonium chloride was added and the organic phase was extracted with EtOAc (3x10 mL). The combined organic layers were dried over anhydrous $MgSO_4$, filtrated and the solvent was removed under reduced pressure. The crude product was purified by flash column chromatography (n-pentane/EtOAc 1:1) to give 3-(6-vinylpyridin-2-yl)butan-1-ol (**10a**) (39.0 mg, 0.22 mmol, 52%, 1% ee) as a colorless oil.

TLC: R_f = 0.36 (*n*-pentane/EtOAc 1:1). **^{1}H-NMR:** (500 MHz, $CDCl_3$) δ = 7.60 (t, *J* = 7.7 Hz, 1H, *H-3*) 7.18 (dd, *J* = 7.7, 1.0 Hz, 1H, *H-2*), 7.08 (dd, *J* = 7.7, 1.0 Hz, 1H, *H-4*), 6.77 (dd, *J* =17.5, 10.9 Hz, 1H, *H-10*), 6.17 (dd, *J* = 17.5, 1.1 Hz, 1H, *H-11*), 5.46 (dd, *J* = 10.9, 1.1 Hz, 1H, *H-11*), 4.35 (s, 1H, O*H*), 3.68 – 3.56 (m, 2H, *H-8*), 3.25 –3.17 (m, 1H, *H-6*), 2.04–1.97 (m, 1H,*H 7*), 1.86 – 1.80 (m, 1H, *H-7*), 1.35 (d, *J* = 7.1 Hz, 3H, *H-9*) ppm.

^{13}C-NMR: (126 MHz $CDCl_3$) δ = 165.3 (*C-5*), 154.7 (*C-1*), 137.5 (*C-3*), 136.7 (*C-10*), 120.4 (*C -4*), 119.1 (*C-2*), 118.5 (*C-11*), 60.3 (*C-8*), 39.1 (*C-6*), 38.7 (*C-7*), 20.4 (*C-9*) ppm. **HRMS (ESI$^+$):** m/z calc. $C_{11}H_{15}NOH$ $[M+H]^+$: 178.1226, found: 178.1220 m/z. **FT-IR:** film, $\tilde{\nu}$ = 3330 (w), 3059 (w), 3015 (w), 2961 (w), 2929 (m), 2871 (w), 1871 (w), 1571 (s), 1452 (s), 1402 (w), 1374 (w), 1296 (w), 1250 (w), 1202 (w), 1159 (m), 1048 (s), 990 (m), 925 (m), 853 (w), 820 (s), 750 (m), 719 (w), 584 (w), 517 (w), 461 (w) cm^{-1}. **S.r:** $[\alpha]_D^{20}$=+12.1 (*c* = 0.5, $CHCl_3$).

HPLC: 92% *n*-hexane, 8% *i*-propanol, 1.0 mL/min, Chiralpak® OD-H (4.6 x 300 mm), ϑ = 20 °C, t_1= 6.65 min, t_2 = 7.11 min, 1% *ee*.

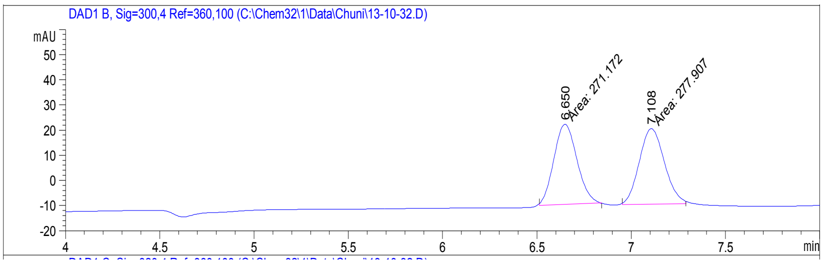

Signal 2: DAD1 B, Sig=300,4 Ref=360,100

Peak #	RetTime [min]	Type	Width [min]	Area [mAU*s]	Height [mAU]	Area %
1	6.650	MM	0.1416	271.17191	31.91220	49.3867
2	7.108	MM	0.1543	277.90714	30.02354	50.6133
Totals :				549.07904	61.93573	

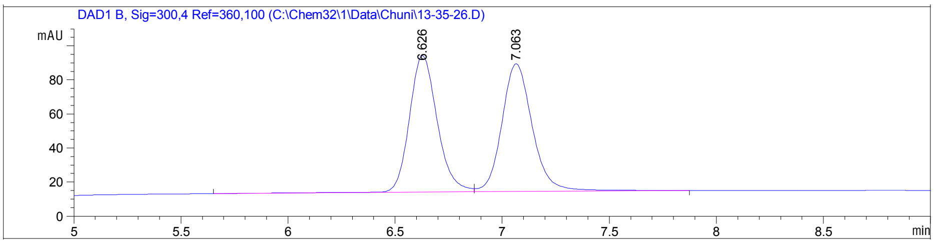

Signal 2: DAD1 B, Sig=300,4 Ref=360,100

Peak #	RetTime [min]	Type	Width [min]	Area [mAU*s]	Height [mAU]	Area %
1	6.626	BV	0.1396	727.03632	80.33241	49.4002
2	7.063	VB	0.1515	744.69189	75.18896	50.5998
Totals :				1471.72821	155.52137	

B) Auxiliary controlled hydrogenation

The absolute configuration of the C3-stereocenter was confirmed by the synthesis of *S,S*-**12** and *R,S*-**12** and HPLC analysis of *S*-**16** and *R*-**16**.

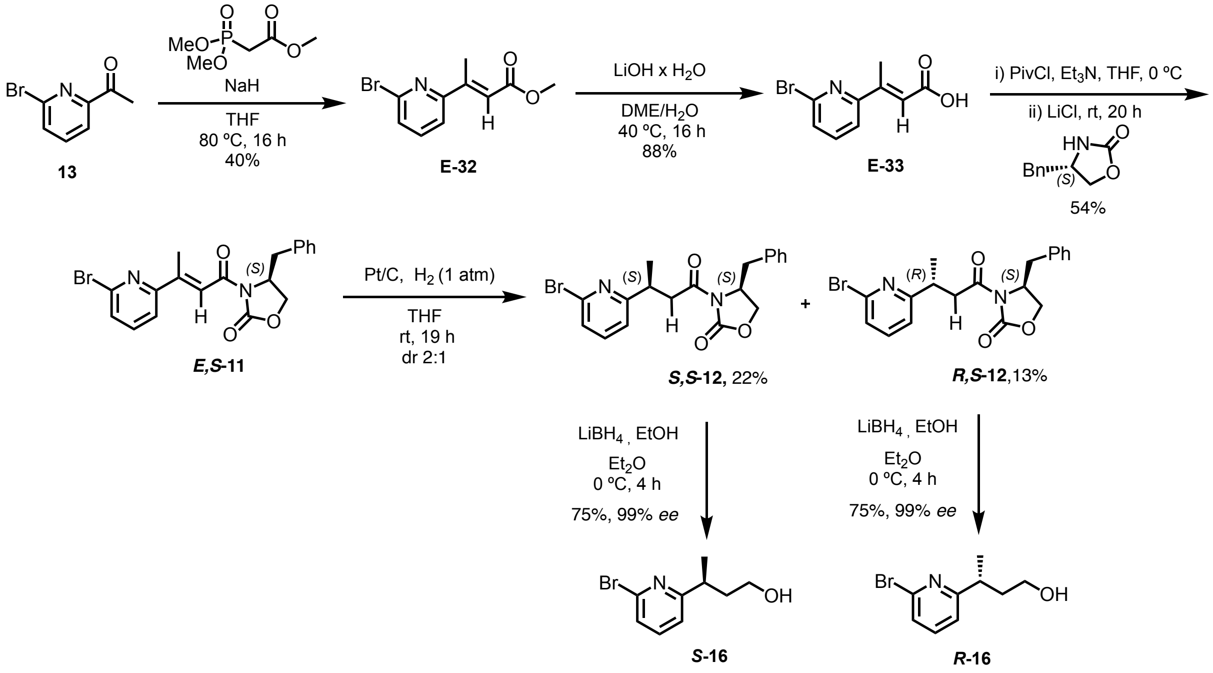

Scheme S1: Syntheses overview for the proof of the C3-stereocenter.

4.1.1 Methyl-(*E*)-3-(6-bromopyridin-2-yl)but-2-enoate (*E*-32) and methyl-(*Z*)-3-(6-bromopyridin-2-yl) but-2-enoate (*Z*-32)

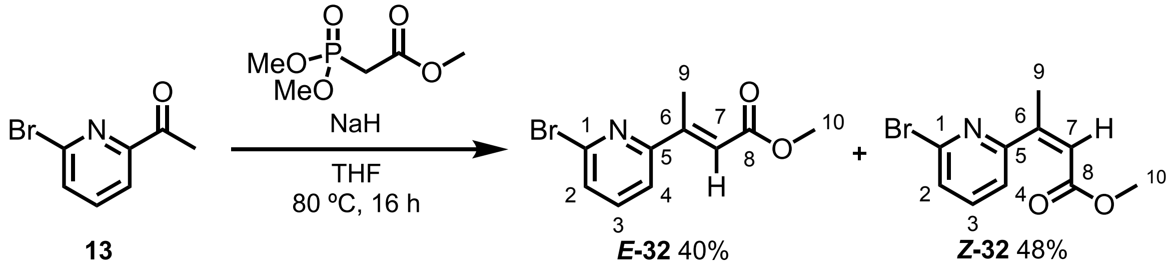

Prepared according to a procedure by Nagai *et. al.*,[1] methyl-2-(dimethoxyphosphoryl)acetate (4.77 mL, 30.0 mmol, 1.20 eq.) was added dropwise to a suspension of NaH (1.40 g, 35.0 mmol, 1.40 eq.) in THF (102 mL) at 0 °C. After the mixture was stirred at 0 °C for 1 h, 1-(6-bromopyridin-2-yl)ethan-1-one (5.00 g, 25.0 mmol, 1.00 eq.) was added to the mixture and stirred for 16 h at 80 °C. After completion of the reaction sat. aq. NH_4Cl (20 mL) and H_2O (20 mL) was added to the reaction mixture. The aqueous layer was extracted with Et_2O (3x30 mL). The organic layers were combined and dried over anhydrous $MgSO_4$. The solvent was removed under reduced pressure and the crude product was purified by flash column chromatography (*n*-pentane/EtOAc 10:1). (*E*)-3-(6-bromopyridin-2-yl)but-2-enoate (*E*-**32**)

(2.57 g, 10.0 mmol, 40 %) was obtained as a colorless solid and methyl-(*Z*)-3-(6-bromopyridin-2-yl)but-2-enoate (*Z*-**32**) (3.04 g, 11.9 mmol, 48 %) was obtained as a colorless resin.

***E*-32:**

TLC: R_f = 0.28 (*n*-pentane/EtOAc 7:1). **^{1}H-NMR:** (500 MHz $CDCl_3$) δ = 7.56 (t, *J* = 7.8 Hz, 1H, *H*-3), 7.48 (dd, *J* = 7.7, 0.9 Hz, 1H, *H*-4), 7.4 (dd, *J* = 7.8, 0.8 Hz, 1H, *H*-2), 6.77 (q, *J* = 1.4 Hz, 1H, *H*-7), 3.78 (s, 3H, *H*-10), 2.57 (d, *J* = 1.4 Hz, 3H, *H*-9) ppm. **^{13}C-NMR**: (126 MHz $CDCl_3$) δ = 167.3 (*C*-8), 159.0 (*C*-5), 151.3 (*C*-6), 141.9 (*C*-1), 139.0 (*C*-3), 128.2 (*C*-2), 120.0 (*C*-7), 119.8 (*C*-4), 51.5 (*C*-10), 15.9 (*C*-9) ppm. **HR-MS** (ESI^+): m/z calc. $C_{10}H_{10}BrNO_2Na$ $[M+Na]^+$: 277.9787, found: 277.9783 m/z. **FT-IR**: film, $\tilde{\nu}$ = 2993 (w), 2948 (w), 1712 (s), 1635 (m), 1571 (w), 1550 (s), 1430 (s), 1408 (w), 1374 (w), 1335 (m), 1284 (m), 1237 (w), 1191 (w), 1177 (w), 1156 (s), 1126 (m), 1097 (w), 1084 (w), 1032 (m), 983 (w), 965 (w), 907 (w), 883 (w), 794 (s), 738 (w), 665 (m), 596 (w), 497 (w), 455 (w). cm^{-1}**. m.p.:** T_m= 51.3-53.0 °C (EtOAc).

***Z*-32**:

TLC: R_f = 0.37 (*n*-pentane/EtOAc, 7:1). **^{1}H-NMR:** (300 MHz $CDCl_3$) δ = 7.54–7.50 (m, 1H, *H*-3), 7.40 (dd, *J* = 7.9, 0.9 Hz, 1H, *H*-2), 7.23 (dd, *J* = 7.6, 0.9 Hz, 1H, *H*-4), 6.01 (q, *J* = 1.5 Hz, 1H, *H*-7), 3.61 (s, 3H, *H*-10), 2.21 (d, *J* = 1.6 Hz, 3H, *H*-9) ppm. **^{13}C-NMR:** (75 MHz $CDCl_3$) δ= 166.3 (*C*-8), 159.3 (*C*-5), 151.2 (*C*-6), 141.4 (*C*-1), 138.2 (*C*-3), 127.1 (*C*-2), 121.5 (*C*-4), 120.0 (*C*-7), 51.5 (*C*-10), 24.6 (*C*-9) ppm. **HR-MS (ESI^+):** m/z calc. $C_{10}H_{10}BrNO_2Na$ $[M+Na]^+$: 277.9787; found: 277.9782 m/z. **FT-IR:** $\tilde{\nu}$ /cm^{-1} = 2985 (w), 2949 (w), 2841 (w), 2108 (w), 1719 (s), 1644 (w), 1576 (m), 1548 (s), 1432 (s), 1393 (w), 1370 (w), 1262 (m), 1170 (s), 1126 (w), 1095 (m), 1034 (m), 983 (m), 918 (w), 863 (w), 796 (s), 768 (w), 741 (w), 660 (m), 581 (w), 534 (w), 466 (w) cm^{-1}.

4.1.2 (*E*)-3-(6-bromopyridin-2-yl)but-2-enoic acid (*E*-33)

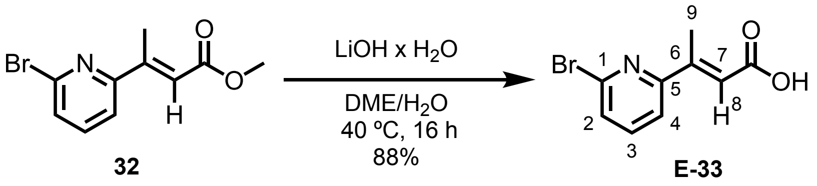

Prepared according to a procedure by Nagai *et. a l*[1], LiOH·H_2O (0.80 g, 19.0 mmol, 2.00 eq.) was added to a solution of methyl ester *E*-**32** (2.44 g, 9.53 mmol, 1.00 eq.) in DME (25 mL) and H_2O (25 mL) at 24 °C. The reaction mixture was stirred for 26 h at 40 °C. The mixture was quenched with aq. HCl (2 M) until pH 2. The aqueous layer was extracted with $CHCl_3$ (3x30 mL). The combined organic layers were washed with brine, dried over anhydrous $MgSO_4$ and concentrated to afford the crude carboxylic acid *E*-**33**, which was used in the next reaction without further purification. (*E*)-3-(6-bromopyridin-2-yl)but-2-enoic acid (*E*-**33**, 2.03 g, 8.39 mmol, 88%) was obtained as a colorless solid.

TLC: R_f = 0.55 ($CHCl_3$/MeOH 10:1). **^{1}H-NMR:** (300 MHz $CDCl_3$) δ = 7.86–7.75 (m, 2H, *H*-3, *H*-4), 7.65 (dd, *J* = 7.1, 1.5 Hz, 1H, *H*-2), 6.69 (q, *J* = 1.4 Hz, 1H, *H*-7), 2.46 (d, *J* = 1.4 Hz, 3H, *H*-9) ppm. **^{13}C-NMR:** (75 MHz $CDCl_3$,) δ = 167.4 (*C*-8), 158.0 (*C*-5), 149.1 (*C*-6), 140.8 (*C*-1), 140.5 (*C*-3), 128.2 (*C*-2), 120.6 (*C*-4), 120.5 (*C*-7), 15.1 (*C*-9) ppm. **HR-MS (ESI$^-$)**: m/z calc. $C_9H_7BrNO_2$ $[M-H]^-$: 239.9666; found: 239.9663 m/z. **FT-IR:** film, $\tilde{\nu}$ = 2910 (w), 2817 (w), 2717 (w), 2605 (m), 2532 (w), 1687 (s), 1625 (s), 1566 (w), 1548 (s), 1426 (s), 1404 (w), 1368 (w), 1328 (m), 1292 (m), 1245 (m), 1213 (w), 1161 (m), 1126 (s), 1097 (w), 1078 (m), 1012 (w), 979 (w), 937 (w), 889 (m), 793 (s), 733 (m), 713 (w), 660 (m), 602 (w), 501 (s), 464 (w) cm^{-1}. **m.p.:** T_m= 175-178 °C ($CHCl_3$).

4.1.3 (*S*,*E*)-4-benzyl-3-(3-(6-bromopyridin-2-yl)but-2-enoyl)oxazolidin-2-one (*E*-(*S*)-11)

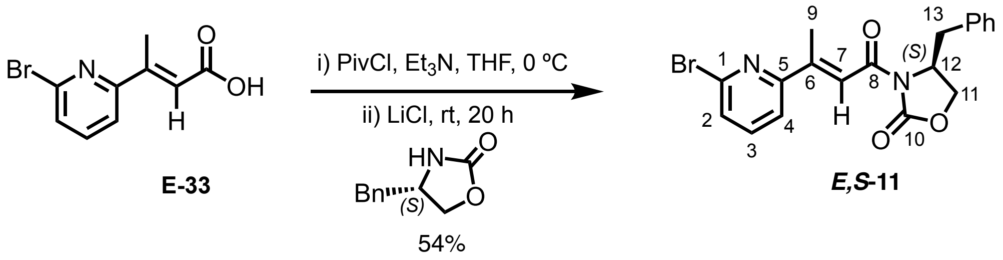

Prepared according to a procedure by Nagai *et. al*,[1] Triethylamine (2.88 mL 20.66 mmol, 5.00 eq.), and pivaloyl chloride (2.02 mL 16.52 mmol, 4.00 eq.), were added to a solution of (*E*)-3-(6-bromopyridin-2-yl)but-2-enoic acid *E*-**33** (1.00 g, 4.13 mmol, 1.00 eq.) in THF (50 mL) at 0 °C. After the reaction mixture was stirred at 0 °C for 1 h, LiCl (0.53 g, 12.39 mmol, 3.00 eq.) and (*S*)-4-benzyloxazolidin-2-one (1.83 g, 10.33 mmol, 2.50 eq.) were added to the mixture at 0 °C. The mixture was stirred at room temperature for 20 h. After completion of the reaction sat. aq. $NaHCO_3$ (20 mL) was added. The aqueous layer was extracted with EtOAc (3x20 mL). The combined organic layers were washed with brine, dried over anhydrous $MgSO_4$ and the solvent was removed under reduced pressure. The crude product was purified by flash column chromatography (*n*-pentane/EtOAc 3:2) to yield (*S*,*E*)-4-benzyl-3-(3-(6-bromopyridin-2-yl)but-2-enoyl)oxazolidin-2-one *E,S*-**11** (0.914 g, 2.09 mmol, 54%) as a colorless solid.

TLC: R_f = 0.48 (*n*-pentane/EtOAc 3:2).**^{1}H-NMR:** (300 MHz $CDCl_3$) δ = 7.77 (q, *J* = 1.4 Hz, 1H, *H*-7), 7.64–7.59 (m, 2H, *H*-3, *H*-4), 7.48 (dd, *J* = 6.2, 2.5 Hz, 1H, *H*-2), 7.38–7.33 (m, 2H, 2x*H*-Ph), 7.31–7.28 (m, 1H, *H*-Ph), 7.28–7.24 (m, 2H, 2x*H*-Ph), 4.79 (ddt, *J* = 9.1, 7.8, 3.2 Hz, 1H, *H*-12), 4.26 (ddd, *J* = 9.0, 7.9, 0.7 Hz, 1H, *H*-11), 4.19 (dd, *J* = 9.0, 3.0 Hz, 1H, *H*-11), 3.33 (dd, *J* = 13.5, 3.4 Hz, 1H, *H*-13), 2.90 (dd, *J* = 13.5, 9.1 Hz, 1H, *H*-13), 2.56 (d, *J* = 1.4 Hz, 3H, *H*-9) ppm.**^{13}C-NMR**: (75 MHz $CDCl_3$) δ = 165.5 (*C*-8), 159.6 (*C*-5), 153.8 (*C*-10), 151.9 (*C*-6), 142.0 (*C*-1), 139.6 (*C*-3), 136.0 (*C*-Ph), 129.9 (2x*C*-Ph), 129.3 (2x*C*-Ph), 128.5 (*C*-2), 127.6 (*C*-Ph), 120.5 (*C*-4, *C*-7), 66.8 (*C*-11), 55.6 (*C*-12), 38.3 (*C*-13), 16.7 (*C*-9) ppm. **HR-MS (ESI$^+$)**: m/z calc. $C_{19}H_{17}BrN_2O_3Na$ $[M+Na]^+$: 423.0315; found: 423.0309 m/z. **FT-IR:** film, $\tilde{\nu}$ = 3028 (w), 2921 (w), 1774 (s), 1676 (m), 1620 (w), 1573 (w), 1551 (m), 1480 (w), 1432 (w), 1386 (w), 1355 (m), 1293 (m), 1248 (w), 1211 (s), 1166 (w), 1129 (w), 1084 (w), 1056 (w), 1011 (w), 983 (w), 959 (w), 923 (w), 877 (w), 837 (w), 794 (m), 759 (w), 736 (w), 703 (w), 661 (w), 574 (w), 505 (w) cm^{-1}. **m.p.:** T_m= 126 - 129 °C (Et_2O), **S.r:** $[\alpha]_D^{20}$=+74.14 (*c* 0.5, $CHCl_3$).

4.1.4 (*S*)-4-benzyl-3-((*S*)-3-(6-bromopyridin-2-yl)butanoyl)-oxazolidin-2-one ((*S,S*)-12) and (*S*)-4-benzyl-3-((*R*)-3-(6-bromopyridin-2-yl)butanoyl)oxazolidin-2-one ((*S,R*)-12)

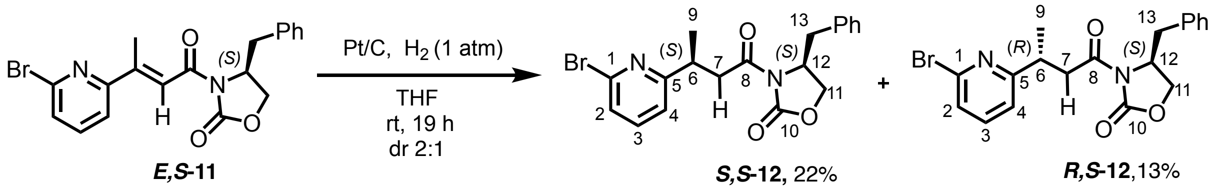

Prepared according to a procedure by Nagai *et. al*,[1] platinum on carbon (10w%, 89 mg, 0.046 mmol, 0.05 eq.) was added to a solution of alkene *E*,*S*-**11** (400 mg, 0.92 mmol, 1.00 eq.) in THF (5 mL) at room temperature. The reaction mixture was stirred for 19 h at room temperature under hydrogen atmosphere with a hydrogen balloon. The suspension was filtered through a pad of celite, washed with EtOAc and concentrated. The crude product was purified by flash column chromatography (*n*-pentane/EtOAc 2:1).). (*S*)-4-benzyl-3-((*S*)-3-(6-bromopyridin-2-yl)butanoyl)oxazolidin-2-one (*S*,*S)*-**12** (80 mg, 0.20 mmol, 22 %) was obtained as a colorless oil and (*S*)-4-benzyl-3-((*R*)-3-(6-bromopyridin-2-yl)butanoyl)oxazolidin-2-one (*S*,*R*)-**12** (48.0 mg, 0.12 mmol, 13%) was obtained colorless crystals. A suitable crystal for structure determination of (*S*,*R*)-**12** was obtained by slow evaporation of a concentrated CH_2Cl_2 solution in a small vial standing in a bigger vial filled with *n*-pentane at room temperature.

(S,*S*)-12:

TLC: R_f = 0.59 (*n*-pentane/EtOAc 2:1). **^{1}H-NMR: (**500 MHz $CDCl_3$) δ = 7.47 (t, *J* = 7.7 Hz, 1H, *H*-3), 7.34–7.30 (m, 2H, 2x*H*-Ph), 7.30–7.24 (m, 2H, *H*-2, *H*-Ph), 7.24–7.21 (m, 1H, *H*-4), 7.21–7.18 (m, 2H, 2x*H*-Ph), 4.61 (dddd, *J* = 9.7, 7.8, 3.4, 2.6 Hz, 1H, *H*-12), 4.22 (ddd, *J* = 8.7, 7.8, 0.8 Hz, 1H, *H*-11), 4.15 (dd, *J* = 9.0, 2.6 Hz, 1H, *H*-11), 3.62 (dd, *J* = 16.7, 9.1 Hz, 1H, *H*-7), 3.55 (dqd, *J* = 9.1, 6.8, 4.6 Hz, 1H, *H*-6), 3.28 (dd, *J* = 13.4, 3.4 Hz, 1H, *H*-13), 3.12 (dd, *J* = 16.8, 4.7 Hz, 1H, *H*-7), 2.76 (dd, *J* = 13.4, 9.7 Hz, 1H, *H*-13), 1.38 (d, *J* = 6.9 Hz, 3H, *H*-9) ppm. **^{13}C-NMR**: (126 MHz $CDCl_3$) δ = 172.2 (*C*-8), 166.3 (*C*-5), 153.7 (*C*-10), 141.5 (*C*-1), 138.9 (*C*-3), 135.5 (*C*-Ph), 129.6 (2x*C*-Ph), 129.1 (2x*C*-Ph), 127.4 (*C*-Ph), 125.8 (*C*-2), 121.0 (*C*-4), 66.4 (*C*-11), 55.4 (*C*-12), 41.4 (*C*-7), 38.1 (*C*-13), 37.3 (*C*-6), 21.1 (*C*-9) ppm. **HR-MS (ESI$^+$):** m/z calc. $C_{19}H_{20}BrN_2O_3$ $[M+H]^+$: 403.0652; found: 403.0644 m/z. **FT-IR:** film, $\tilde{\nu}$ = 3028 (w), 2969 (w), 2927 (w), 1777 (s), 1697 (m), 1582 (w), 1552 (m), 1480 (w), 1455 (w), 1434 (w), 1385 (m), 1290 (w), 1212 (m), 1159 (w), 1123 (w), 1103 (w), 1078 (w), 1051 (w), 993 (w), 909 (w), 838 (w), 796 (w), 742 (w), 703 (m), 663 (w), 595 (w), 502 (w) cm^{-1}. **S.r:** $[\alpha]_D^{20}$=+103.02 (*c* 0.5, $CHCl_3$).

***(R,S)*-12:**

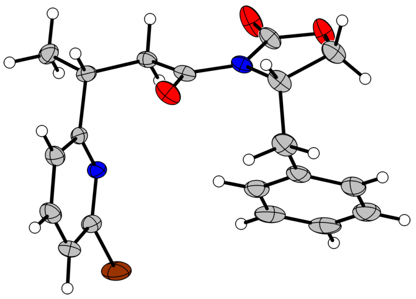

TLC: R_f = 0.46 (*n*-pentane/EtOAc 2:1). **^{1}H-NMR:** (500 MHz $CDCl_3$) δ = 7.47 (t, *J* = 7.7 Hz, 1H, *H*-3), 7.36–7.30 (m, 2H, 2x*H*-Ph), 7.29 (dd, *J* = 7.9, 0.9 Hz, 1H, *H*-2), 7.27–7.24 (m, 1H, *H*-Ph), 7.22 (ddd, *J* = 7.6, 0.9, 0.4 Hz, 1H, *H*-4), 7.17–7.13 (m, 2H, 2x*H*-Ph), 4.66 (ddt, *J* = 9.3, 7.9, 3.2 Hz, 1H, *H*-12), 4.21 (ddd, *J* = 9.1, 7.9, 0.7 Hz, 1H, *H*-11), 4.15 (dd, *J* = 9.0, 3.0 Hz, 1H, *H*-11), 3.64 (dd, *J* = 17.2, 8.5 Hz, 1H, *H*-7), 3.53 (dqd, *J* = 8.5, 7.0, 5.4 Hz, 1H, *H*-6), 3.19–3.10 (m, 2H, *H*-13, *H*-7), 2.75 (dd, *J* = 13.5, 9.3 Hz, 1H, *H*-13), 1.37 (d, *J* = 7.0 Hz, 3H, *H*-9) ppm. **^{13}C-NMR:** (126 MHz $CDCl_3$) δ = 172.1 (*C*-8), 166.4 (*C*-5), 153.6 (*C*-10), 141.6 (*C*-1), 138.9 (*C*-3), 135.4 (*C*-Ph), 129.6 (2x*C*-Ph), 129.1 (2x*C*-Ph), 127.4 (*C*-Ph), 125.8 (*C*-2), 121.0 (*C*-4), 66.3 (*C*-11), 55.1 (*C*-112), 41.5 (*C*-7), 37.9 (*C*-13), 37.2 (*C*-6), 21.0 (*C*-9) ppm. **HR-MS (ESI$^+$):** m/z calc. $C_{19}H_{20}BrN_2O_3$ $[M+H]^+$: 403.0652; found: 403.0643 m/z. **FT-IR:** film, $\tilde{\nu}$ =3062 (w), 3028 (w), 2969 (w), 2929 (w), 1776 (s), 1697 (m), 1582 (w), 1552 (m), 1480 (w), 1455 (w), 1434 (w), 1386 (m), 1290 (w), 1214 (m), 1194 (w), 1159 (w), 1123 (w), 1103 (m), 1077 (w), 1052 (w), 993 (w), 908 (w), 836 (w), 795 (w), 733 (w), 703 (m), 663 (w), 645 (w), 622 (w), 593 (w), 503 (w) cm^{-1}. **m.p:** T_m= 110 - 112 °C (EtOAc). **S.r**$[\alpha]_D^{20}$=+112.53 (*c* 0.5, $CHCl_3$).

4.1.5 (*S*)-3-(6-bromopyridin-2-yl)butan-1-ol ((*S*)-16)

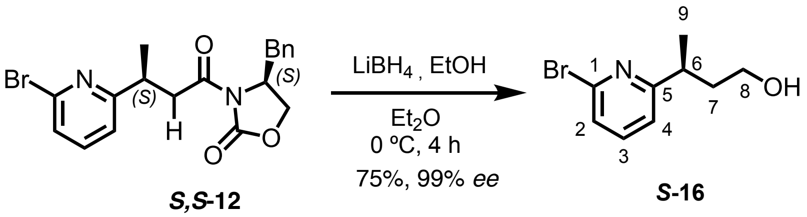

***S,S*-12** → ***S*-16**

Ethanol (0.007 mL, 0.128 mmol, 1.50 eq.) and lithium borohydride (3.7 mg, 0.171 mmol, 2.00 eq.) were added to a solution of (*S*)-4-benzyl-3-((*S*)-3-(6-bromopyridin-2-yl)butanoyl)oxazolidin-2-one (*S,S*-**12**, 36.5 mg, 0.085 mmol, 1.00 eq.) in diethyl ether (1.00 mL) at 0°C. The mixture was stirred at 0°C for 1 h, warmed up to room temperature and stirred for additional 2 h. After completion the reaction was quenched with NaOH (1 M, 2 mL) and extracted with diethyl ether (3x10 mL). The combined organic layers were washed with brine, dried anhydrous over $MgSO_4$ and the solvent was removed under reduced pressure. The crude product was purified by flash column chromatography (*n*-pentane/EtOAc 2:1). (*S*)-3-(6-bromopyridin-2-yl)butan-1-ol (*S*)-**16** (15.0 mg, 0.064 mmol, 75%, 99% *ee*) was obtained as a colorless oil.

TLC: R_f = 0.24 (*n*-pentane/EtOAc 2:1). **^{1}H-NMR:** (500 MHz, $CDCl_3$) δ = 7.47 (t, 1H, *J* = 7.7 Hz, *H-3*), 7.30 (dd, 1H, *J* = 7.7, 0.8 Hz, *H-2)*, 7.13 (dd, 1H, *J* = 7.7, 0.8 Hz, *H-4)*, 3.65-3.54 (m, 2H, *H-8*), 3.12-3.08 (m, 1H, *H-6*), 2.24 (br s, 1H, O*H),* 1.95-1.87 (m, 2H, *H-7*), 1.31 (d, 3H, J = 6.9 Hz, *H-9*) ppm. **^{13}C-NMR:** (500 MHz, $CDCl_3$) δ = 167.7 (*C-5*), 141.6 (*C-1*), 139.1 (*C-3*), 125.7 (*C-2*), 120.5 (*C-4*), 60.7 (*C-8*), 39.4 (*C-7*), 38.5 (*C-6*), 20.6 (*C-9*) ppm. **HR-MS (ESI$^+$):** m/z calc. $C_9H_{12}BrNOH$ $[M+H]^+$: 230.0175, found: 230.0171 m/z. **FT-IR:** film, $\tilde{\nu}$ = 3337 (w), 2963 (w), 2931 (m), 2874 (w), 2118 (w), 1581 (m), 1552 (s), 1457 (w), 1432 (m), 1407 (s), 1373 (w), 1352 (w), 1159 (m), 1126 (m), 1082 (w), 1045 (s), 986 (m), 953 (w), 903 (w), 852 (w), 794 (s), 777 (w), 742 (m), 670 (w), 624 (w), 520 (w), 415 (w) cm^{-1}. **S.r:** $[\alpha]_D^{20}$=+21.5 (*c* 0.5, $CHCl_3$). **HPLC:** (*S*)-**16**: 99% *n*-hexane, 1% *i*-propanol, 1 mL/min, Chiralcel OD-H (4.6 × 250 mm), t_{major} = 42.06 min, t_{minor} = 43.63 min, >99% *ee*.

racemic reference:

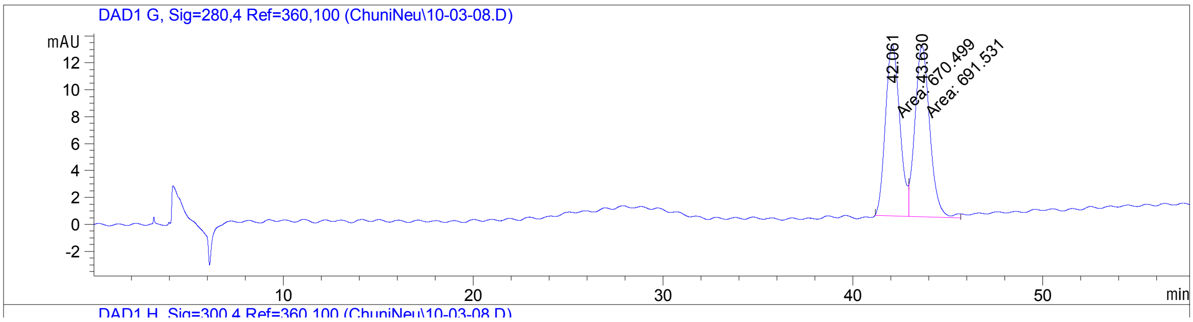

Signal 5: DAD1 G, Sig=280,4 Ref=360,100

Peak #	RetTime [min]	Type	Width [min]	Area [mAU*s]	Height [mAU]	Area %
1	42.061	MF	0.8767	670.49908	12.74635	49.2279
2	43.630	FM	0.9064	691.53082	12.71585	50.7721

Totals : 1362.02991 25.46220

(*S*)-**16**: t_R = 42.12 min, >99% *ee*

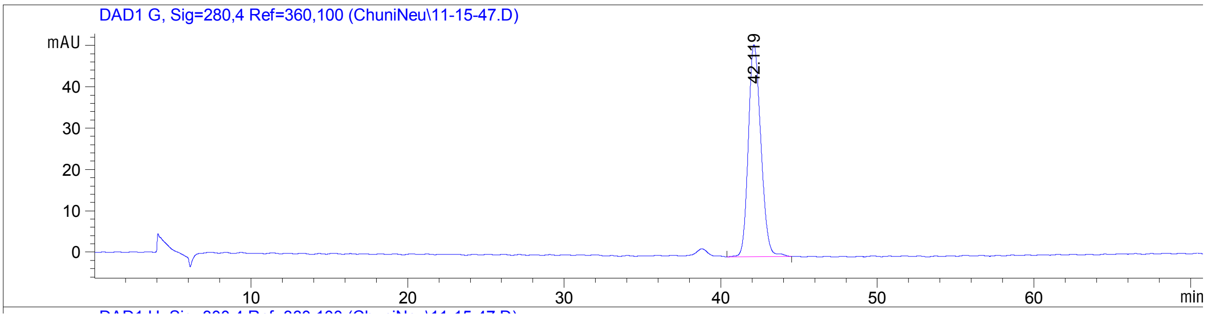

Signal 5: DAD1 G, Sig=280,4 Ref=360,100

Peak #	RetTime [min]	Type	Width [min]	Area [mAU*s]	Height [mAU]	Area %
1	42.119	BB	0.8608	2853.78345	51.31450	100.0000

Totals : 2853.78345 51.31450

4.1.6 (*R*)-3-(6-bromopyridin-2-yl)butan-1-ol ((*R*-)16)

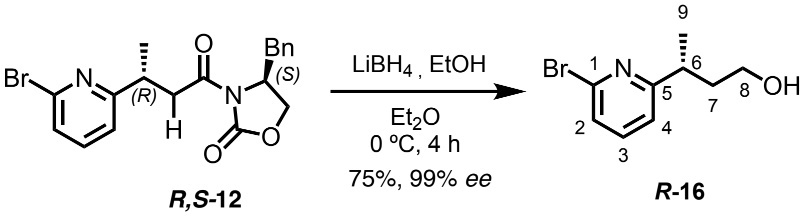

***R,S*-12** ***R*-16**

Alcohol (*R*)-**16** was prepared analogously to (*S*)-**16** (*see 4.1.5*). Oxazolidinone (R,*S*)-**12** (37.8 mg, 0.094 mmol, 1.00 eq.), $LiBH_4$ (4.1 mg, 0.187 mmol, 2.00 eq.), EtOH (8 µL, 0.141 mmol, 1.50 eq.) and Et_2O (1 mL) yielded alcohol (*R*)-**16** (16.1 mg, 0.07 mmol, 75%, 99% *ee*) as a colorless oil. The analytical data are in accordance with (*S*)-**16**.

S.r: $[\alpha]_D^{20}$= -18.1 (*c* 0.5, $CHCl_3$).

HPLC: (*R*)-**16**: 99% *n*-hexane, 1% *i*-propanol, 1 mL/min, Chiralcel OD-H (4.6 × 250 mm), t_{minor} = 42.06 min, t_{major} = 43.61 min, 99% *ee*.

racemic reference:

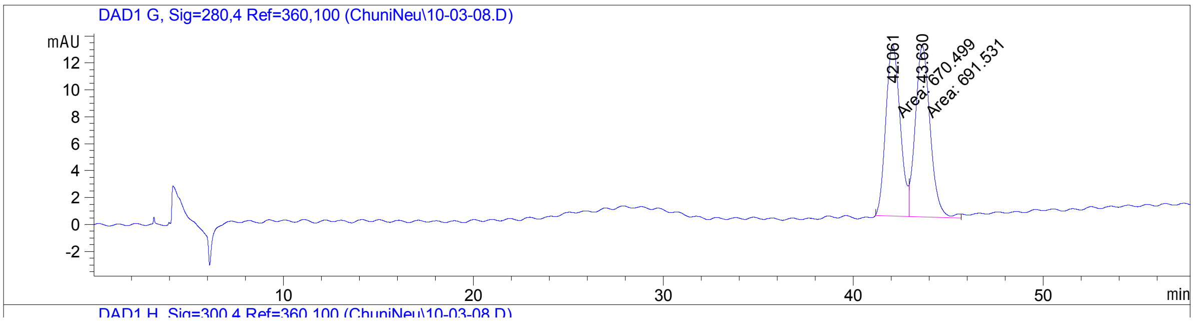

Signal 5: DAD1 G, Sig=280,4 Ref=360,100

Peak #	RetTime [min]	Type	Width [min]	Area [mAU*s]	Height [mAU]	Area %
1	42.061	MF	0.8767	670.49908	12.74635	49.2279
2	43.630	FM	0.9064	691.53082	12.71585	50.7721
Totals :				1362.02991	25.46220	

(*R*)-**16**: t_R = 43.61 min, >99% *ee*

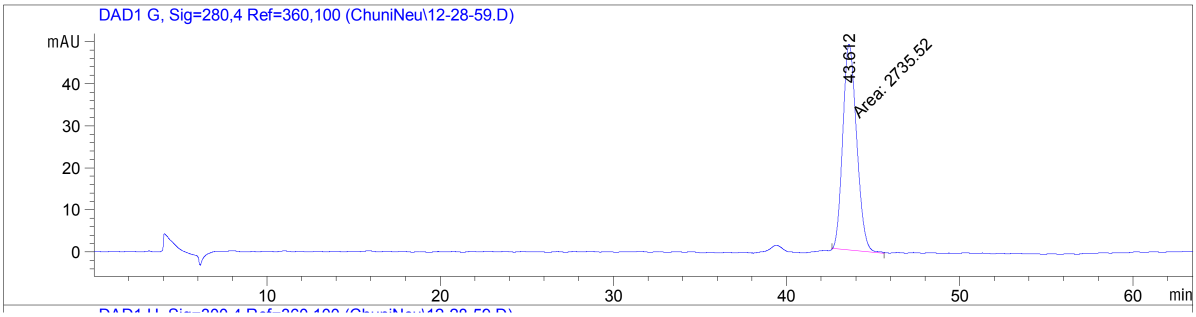

Signal 5: DAD1 G, Sig=280,4 Ref=360,100

Peak #	RetTime [min]	Type	Width [min]	Area [mAU*s]	Height [mAU]	Area %
1	43.612	MM	0.9314	2735.52075	48.95072	100.0000

Totals : 2735.52075 48.95072

4.2 NMR-Data

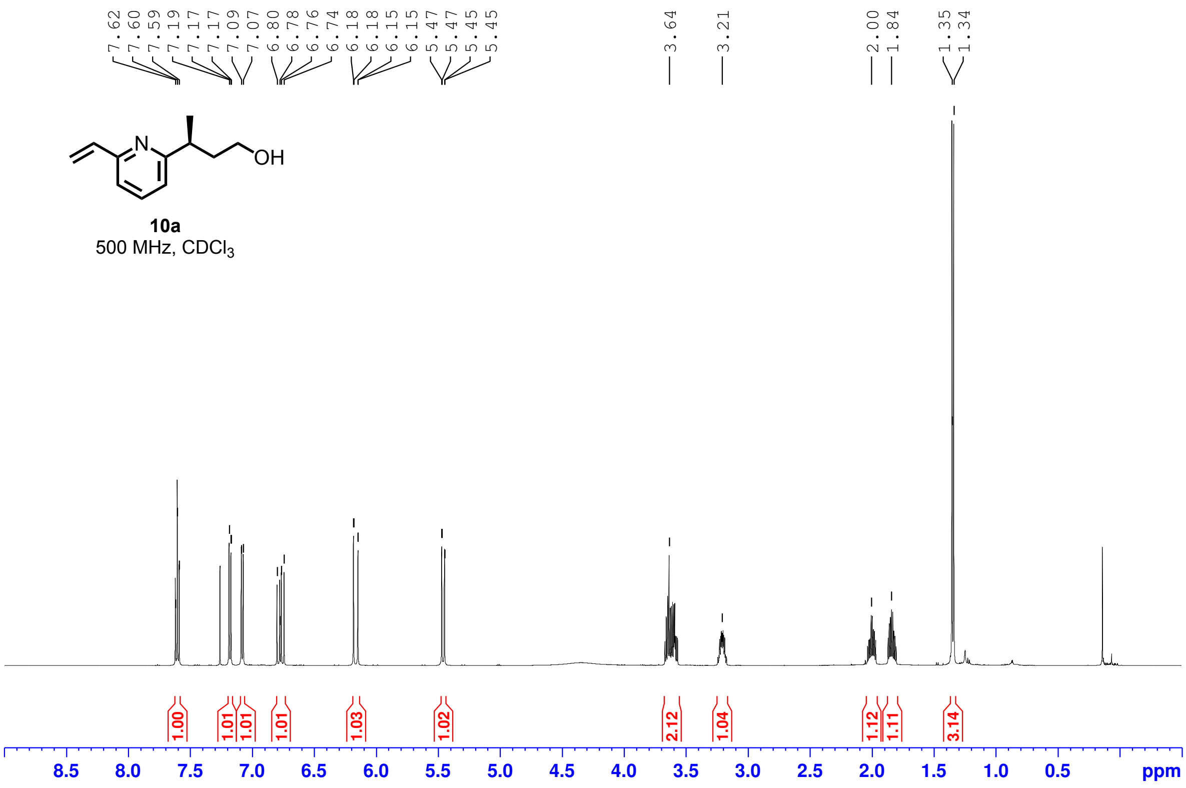

7.62
7.60
7.59
7.19
7.17
7.17
7.09
7.07
6.80
6.78
6.76
6.74
6.18
6.18
6.15
6.15
5.47
5.47
5.45
5.45
3.64
3.21
2.00
1.84
1.35
1.34
N
OH
10a
500 MHz, CDCl3
1.00
1.01
1.01
1.01
1.03
1.02
2.12
1.04
1.12
1.11
3.14
8.5
8.0
7.5
7.0
6.5
6.0
5.5
5.0
4.5
4.0
3.5
3.0
2.5
2.0
1.5
1.0
0.5
ppm

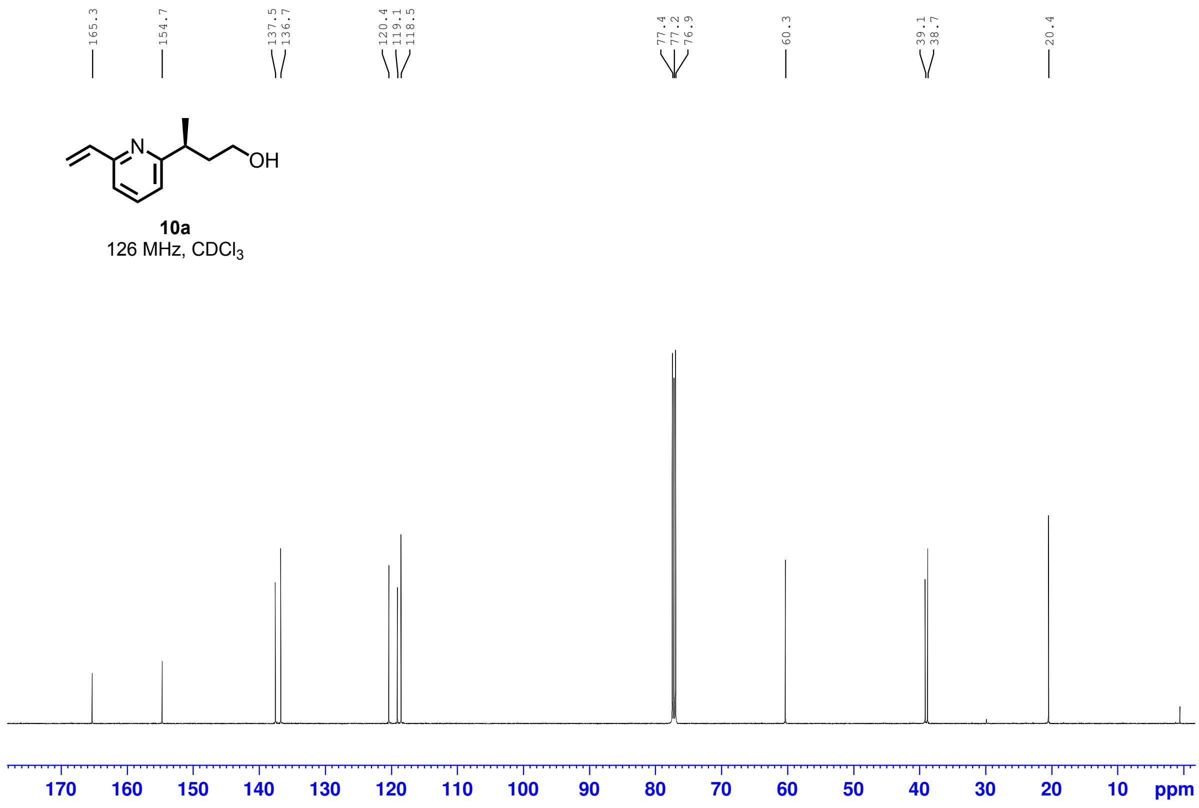

165.3
154.7
137.5
136.7
120.4
119.1
118.5
77.4
77.2
76.9
60.3
39.1
38.7
20.4
N
OH
10a
126 MHz, CDCl3
170
160
150
140
130
120
110
100
90
80
70
60
50
40
30
20
10
ppm

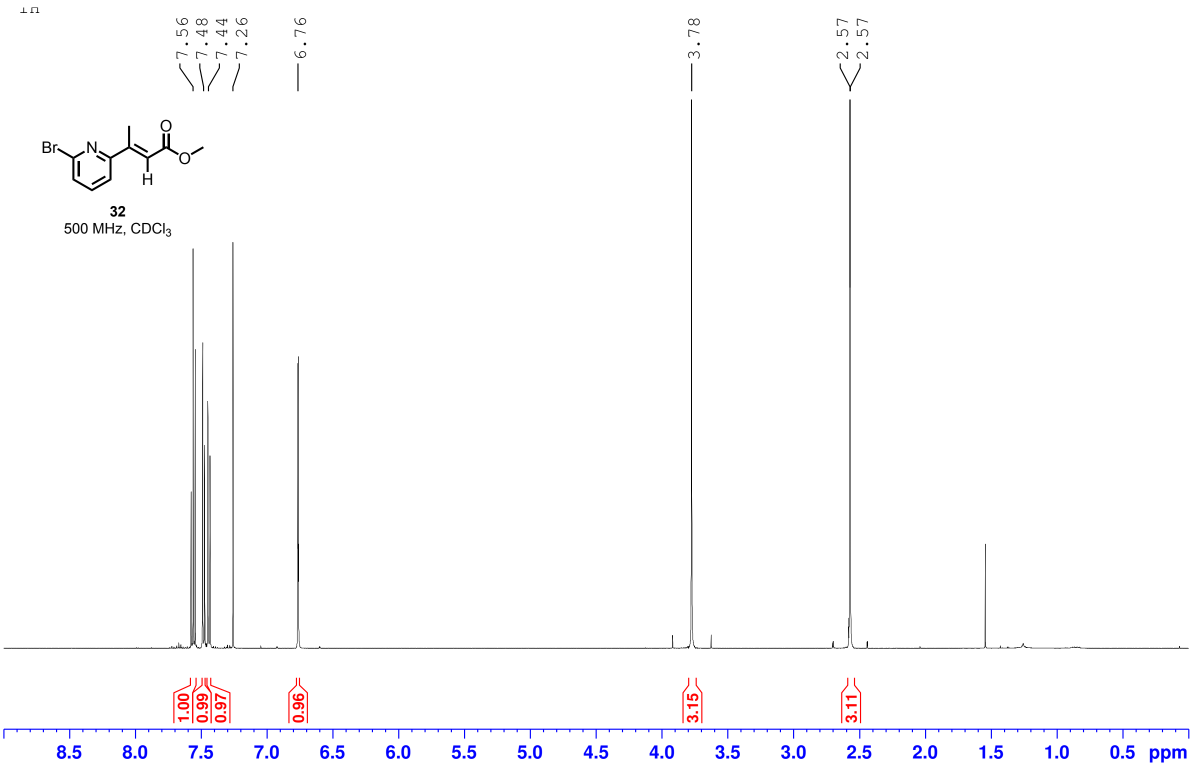

32
500 MHz, $CDCl_3$

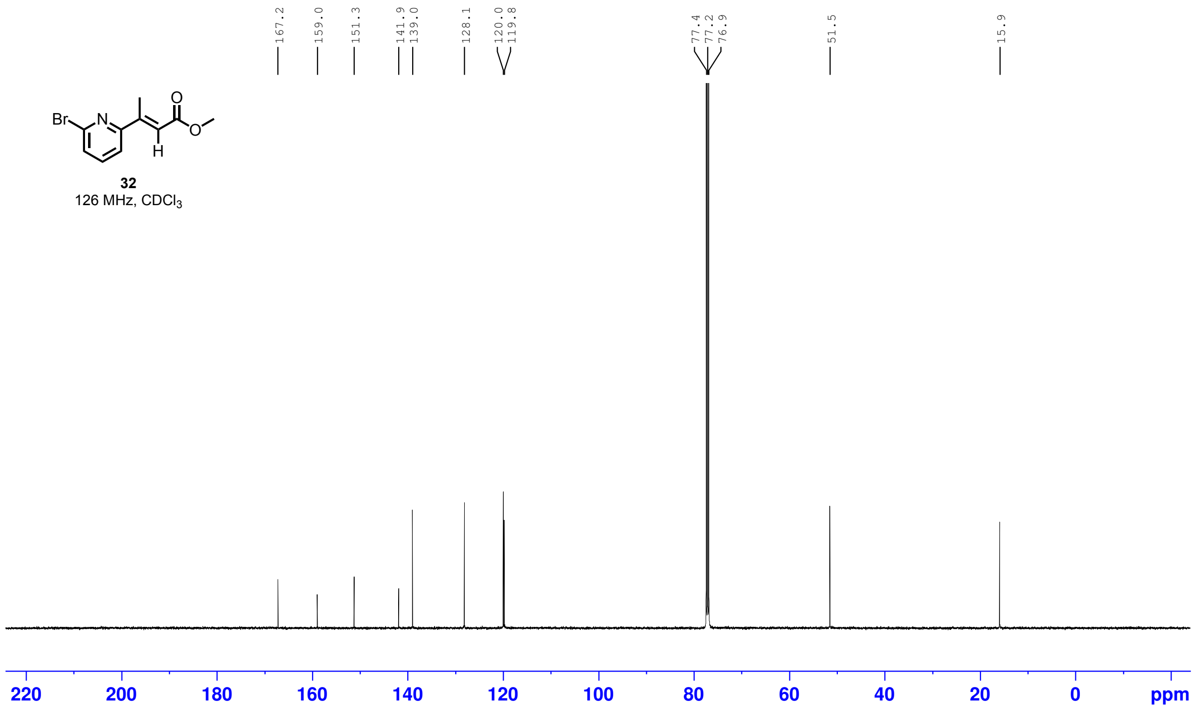

32
126 MHz, $CDCl_3$

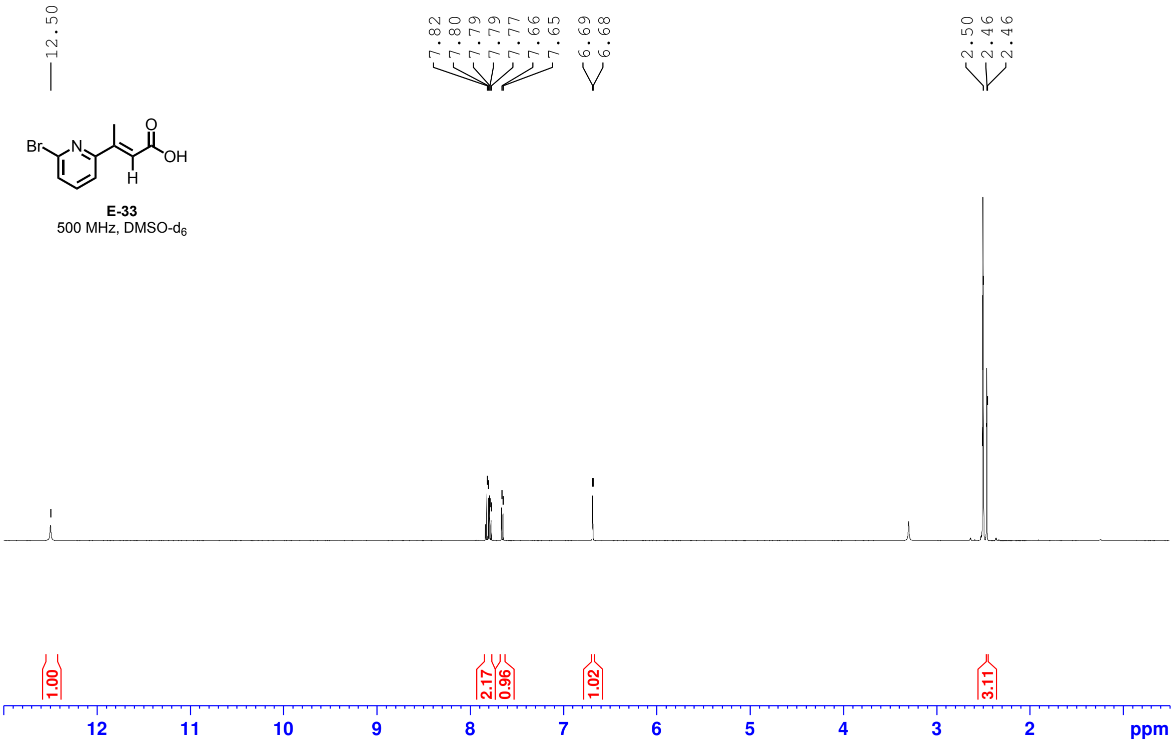

12.50
7.82
7.80
7.79
7.79
7.77
7.66
7.65
6.69
6.68
2.50
2.46
2.46
Br
N
O
OH
H
E-33
500 MHz, DMSO-d6
1.00
2.17
0.96
1.02
3.11
12
11
10
9
8
7
6
5
4
3
2
ppm

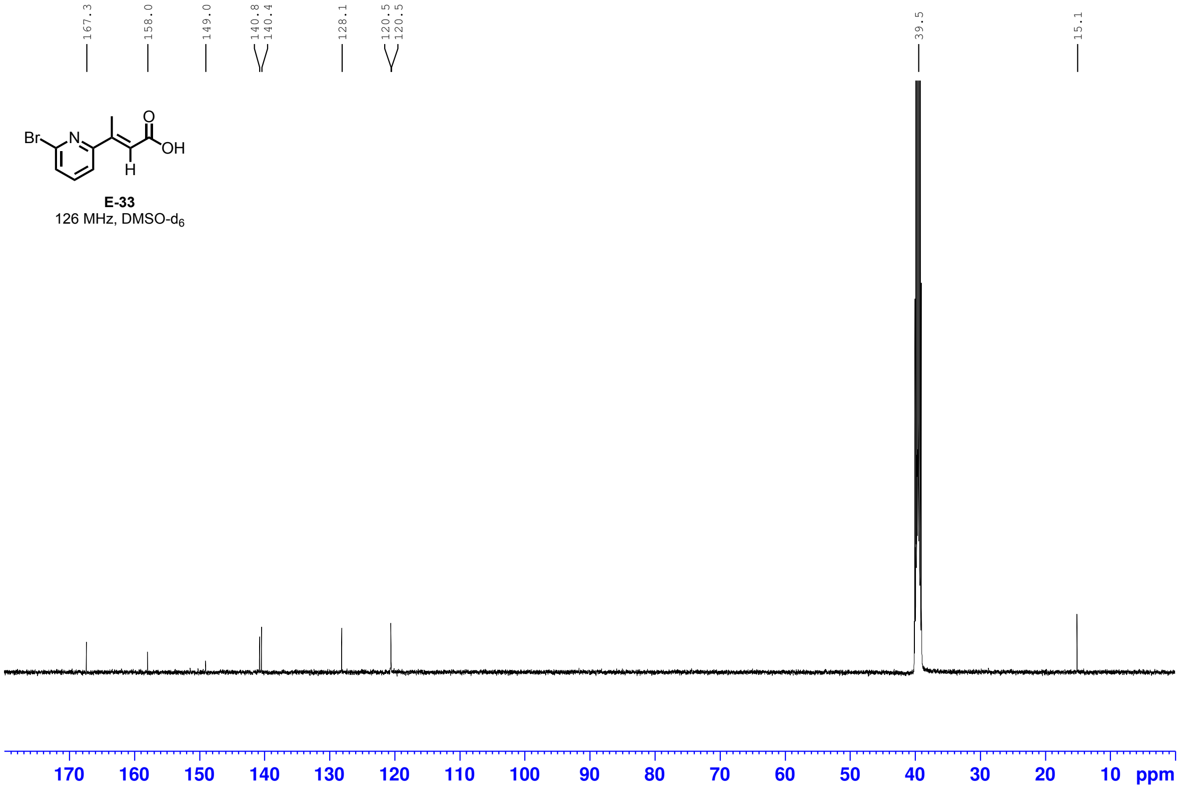

167.3
158.0
149.0
140.8
140.4
128.1
120.5
120.5
39.5
15.1
Br
N
O
OH
H
E-33
126 MHz, DMSO-d6
170
160
150
140
130
120
110
100
90
80
70
60
50
40
30
20
10
ppm

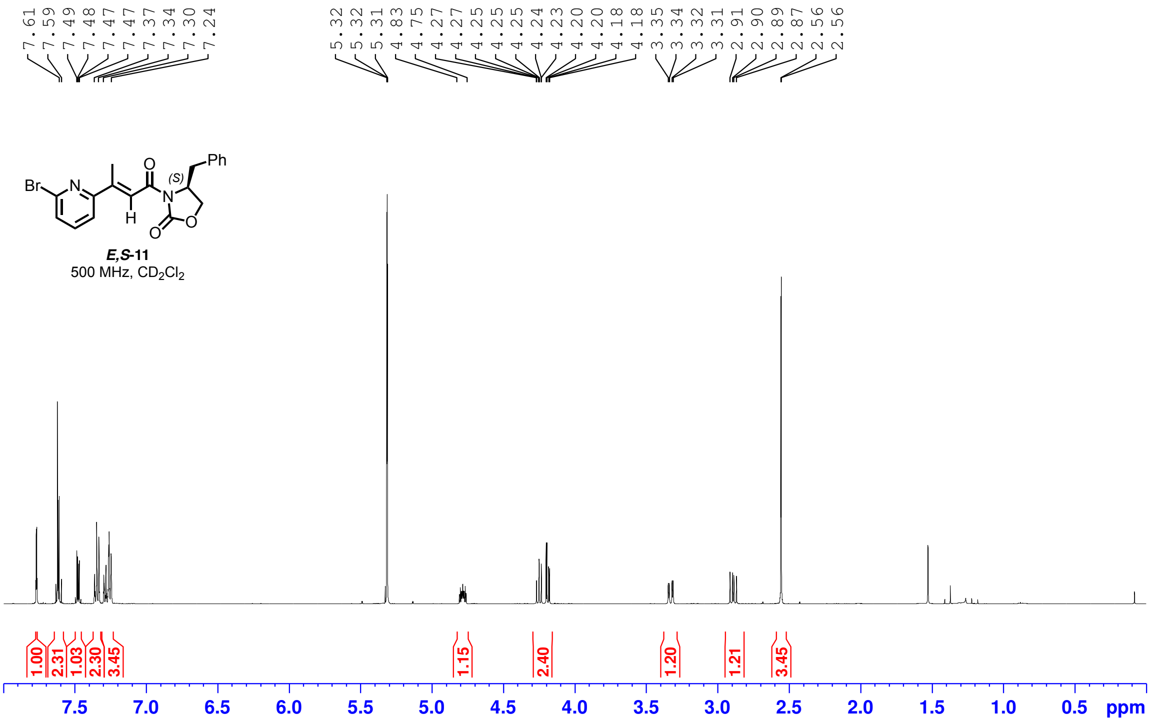
7.61
7.59
7.49
7.48
7.47
7.47
7.37
7.34
7.30
7.24
5.32
5.32
5.31
4.83
4.75
4.27
4.27
4.25
4.25
4.25
4.24
4.23
4.20
4.20
4.18
4.18
3.35
3.34
3.32
3.31
2.91
2.90
2.89
2.87
2.56
2.56
Br
N
(S)
O
Ph
H
O
O
E,S-11
500 MHz, CD_2Cl_2
1.00
2.31
1.03
2.30
3.45
1.15
2.40
1.20
1.21
3.45
7.5
7.0
6.5
6.0
5.5
5.0
4.5
4.0
3.5
3.0
2.5
2.0
1.5
1.0
0.5
ppm

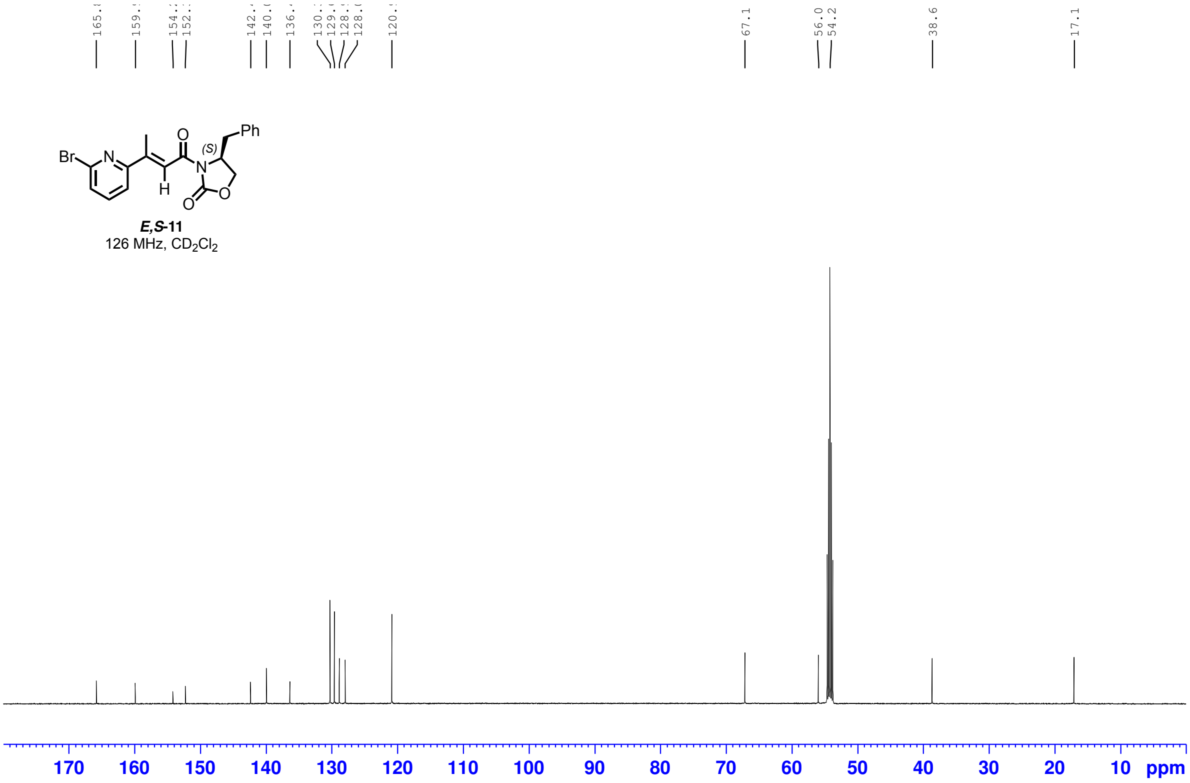
67.1
56.0
54.2
38.6
17.1
Br
N
(S)
O
Ph
H
O
O
E,S-11
126 MHz, CD_2Cl_2
170
160
150
140
130
120
110
100
90
80
70
60
50
40
30
20
10
ppm

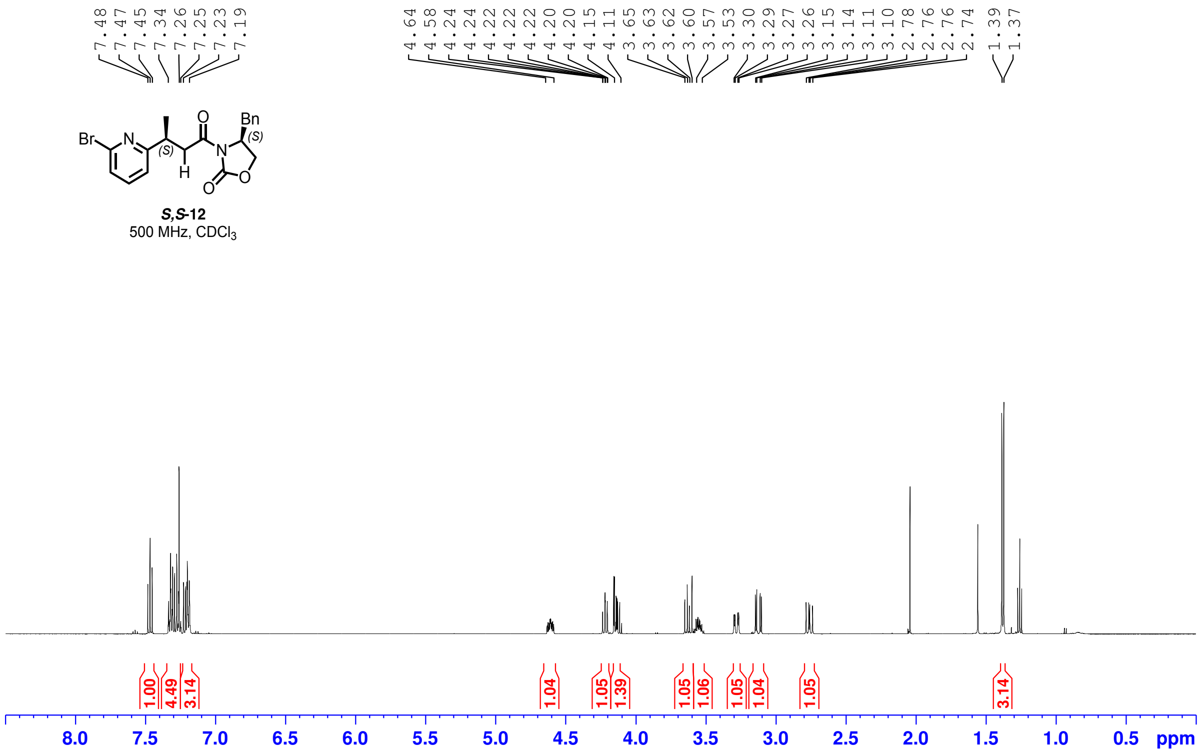
7.48 7.47 7.45 7.34 7.26 7.25 7.23 7.19
4.64 4.58 4.24 4.24 4.22 4.22 4.22 4.20 4.20 4.15 4.11 3.65 3.63 3.62 3.60 3.57 3.53 3.30 3.29 3.27 3.26 3.15 3.14 3.11 3.10 2.78 2.76 2.76 2.74 1.39 1.37
S,S-12
500 MHz, CDCl3
1.00 4.49 3.14 1.04 1.05 1.39 1.05 1.06 1.05 1.04 1.05 3.14
8.0 7.5 7.0 6.5 6.0 5.5 5.0 4.5 4.0 3.5 3.0 2.5 2.0 1.5 1.0 0.5 ppm

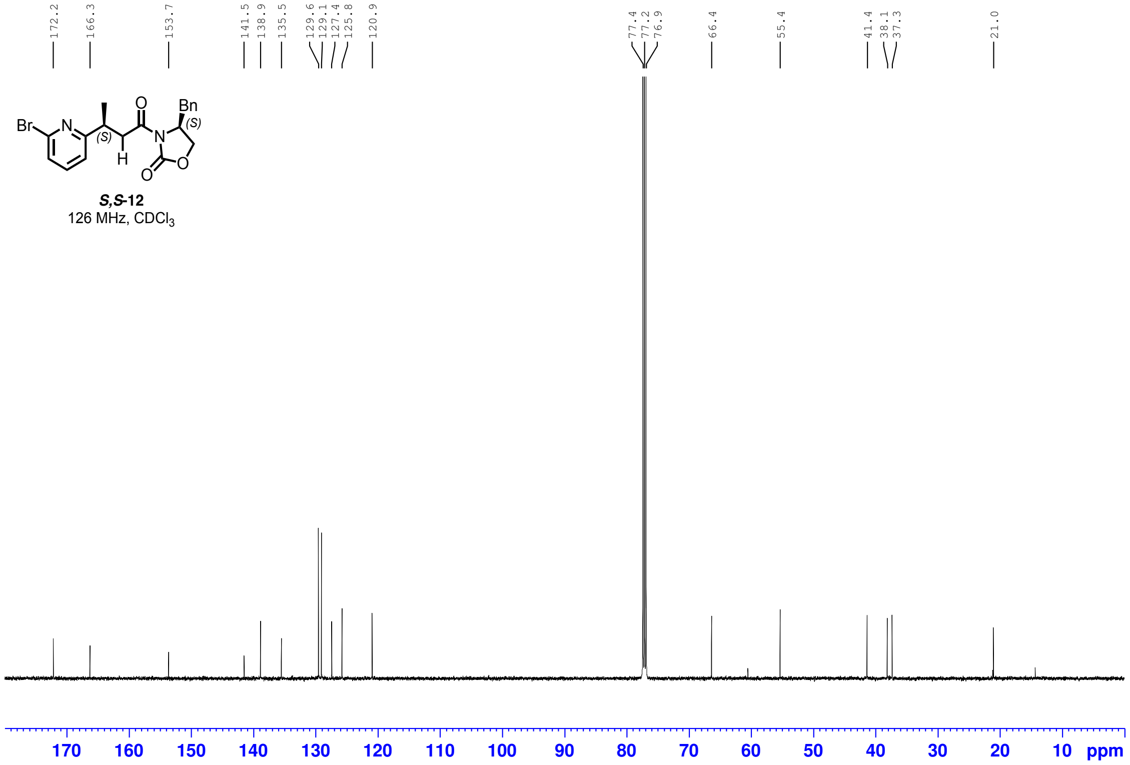
172.2 166.3 153.7 141.5 138.9 135.5 129.6 129.1 127.4 125.8 120.9 77.4 77.2 76.9 66.4 55.4 41.4 38.1 37.3 21.0
S,S-12
126 MHz, CDCl3
170 160 150 140 130 120 110 100 90 80 70 60 50 40 30 20 10 ppm

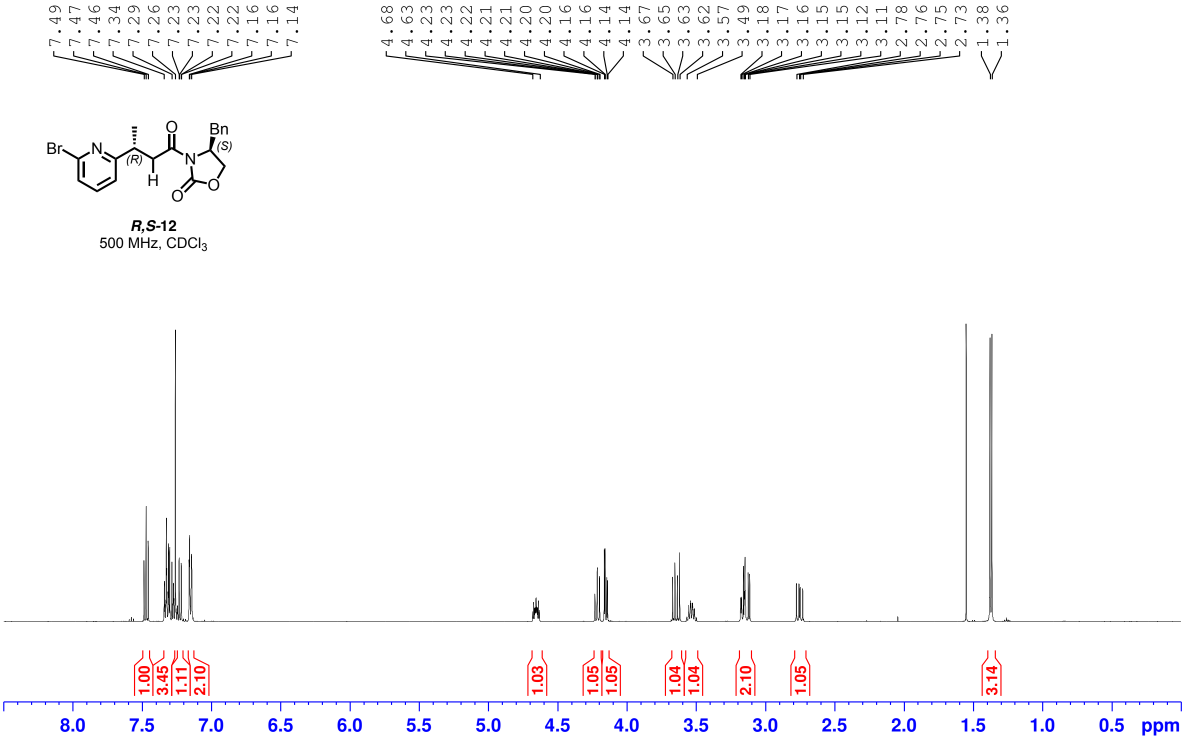
R,S-12
500 MHz, CDCl3

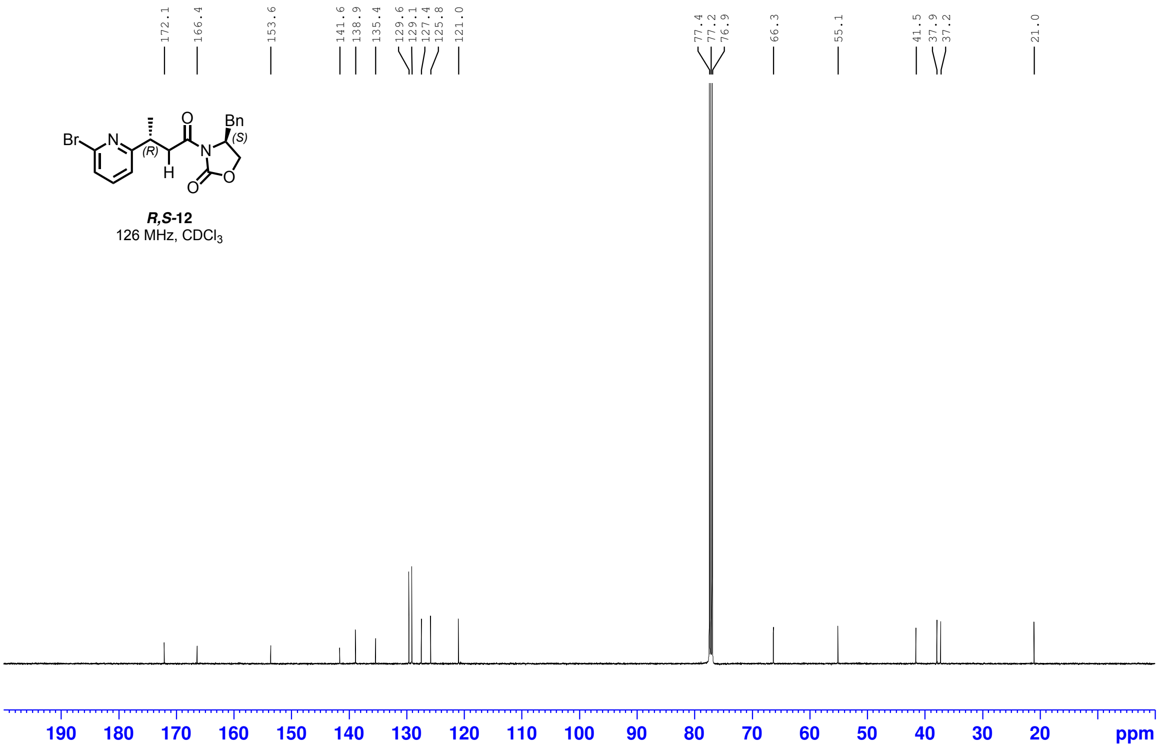
R,S-12
126 MHz, CDCl3

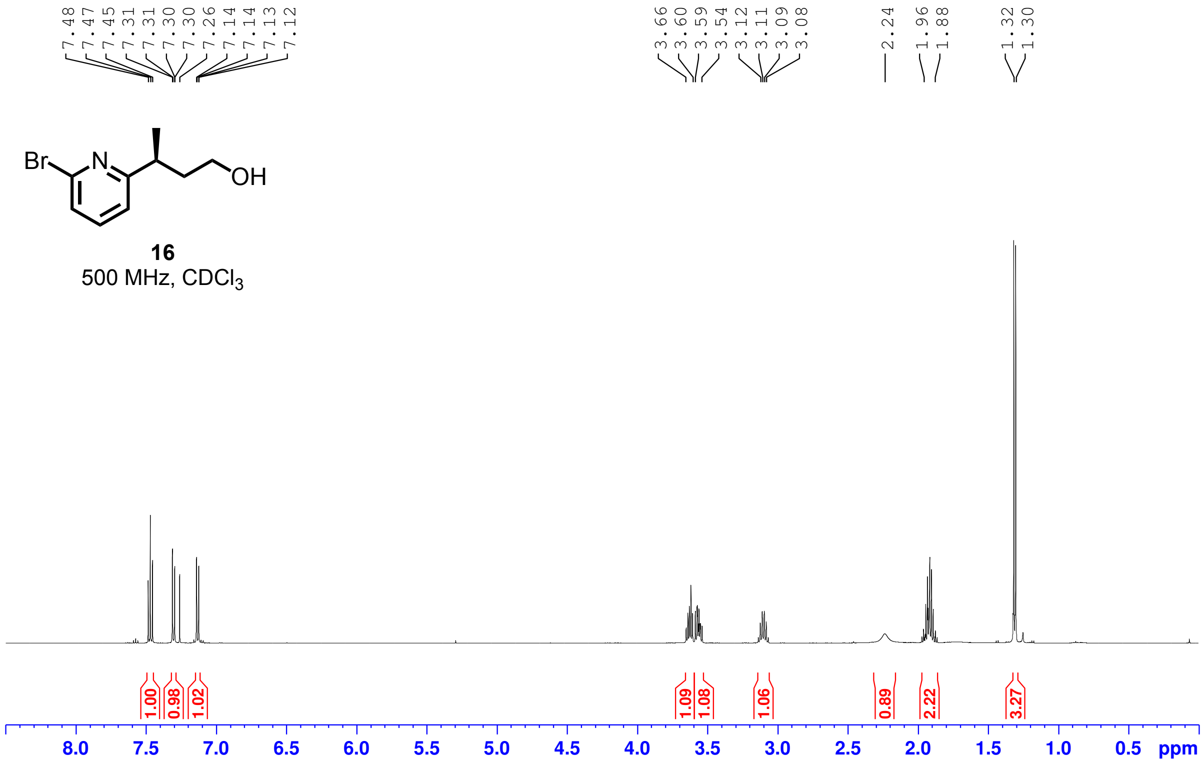

16
500 MHz, $CDCl_3$

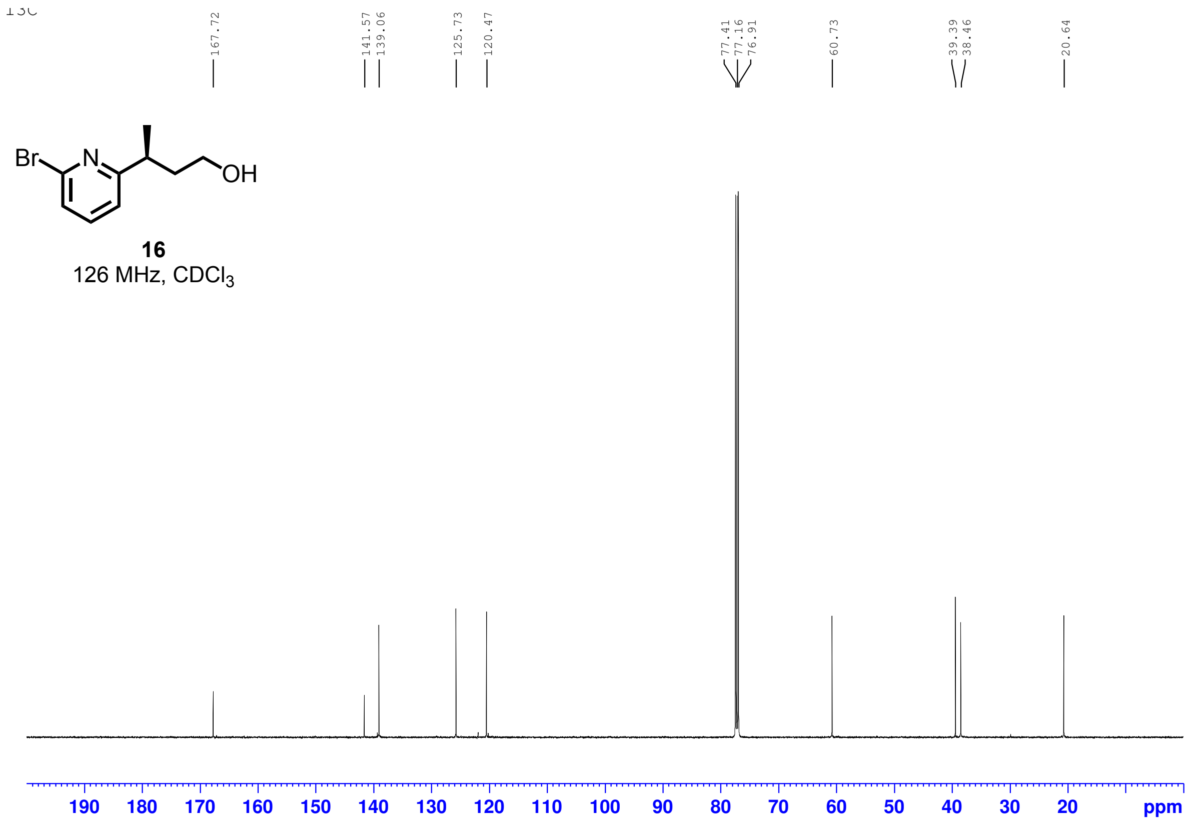

16
126 MHz, $CDCl_3$

5 X-Ray Data

5.1 X-Ray Data of (*S,R-)*12

A suitable crystal of $C_{19}H_{19}BrN_2O_3$ (*S,R)*-**12** was selected under inert oil and mounted using a MiTeGen loop. Intensity data of the crystal were recorded with a D8 Venture diffractometer (Bruker AXS). The instrument was operated with Mo-Kα radiation (0.71073 Å, microfocus source) and equipped with a PHOTON III C14 detector. Evaluation, integration and reduction of the diffraction data was carried out using the Bruker APEX 5 software suite.[8] Multi-scan and numerical absorption corrections were applied using the SADABS program.[9,10] The structure was solved using dual-space methods (SHELXT-2018/2) and refined against F^2 (SHELXL-2019/1 using ShelXle interface).[11–13] All non-hydrogen atoms were refined with anisotropic displacement parameters. The hydrogen atoms were refined using the "riding model" approach with isotropic displacement parameters 1.2 times (1.5 times for terminal methyl groups) of that of the preceding carbon atom. CCDC 2556159 contains the supplementary crystallographic data for this paper. These data can be obtained free of charge from The Cambridge Crystallographic Data Centre via www.ccdc.cam.ac.uk/structures.

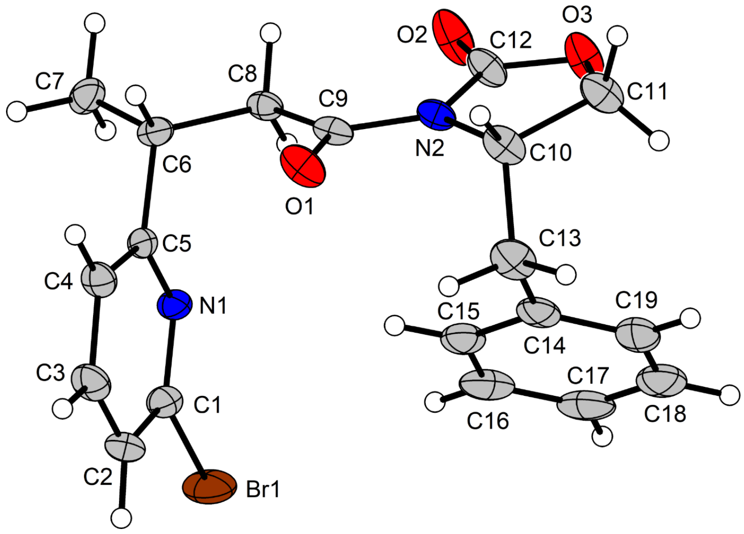

* Displacement ellipsoids are shown at 50% probability level at 100 K. The hydrogen atoms are shown with arbitrary radii.

Table 1. Selected crystallographic data and details of the structure determination for $C_{19}H_{19}BrN_2O_3$.

Identification code	CILB021D2
Empirical formula	$C_{19}H_{19}BrN_2O_3$
Molar mass / g·mol^{-1}	403.27
Space group (No.)	$P2_1$ (4)
a / Å	6.2091(3)
b / Å	15.0838(5)
c / Å	9.8697(4)
β / °	104.4100(10)
V / Å^3	895.28(6)
Z	2
$\rho_{calc.}$ / g·cm^{-3}	1.496
μ / mm^{-1}	2.316
Color	colorless
Crystal habitus	block
Crystal size / mm^3	0.791 x 0.403 x 0.258
T / K	100
λ / Å	0.71073 (Mo-K$_\alpha$)
θ range / °	2.130 to 28.293
Range of Miller indices	$-8 \leq h \leq 8$
	$-20 \leq k \leq 20$
	$-13 \leq l \leq 13$
Absorption correction	multi-scan and numerical
T_{min}, T_{max}	0.2830, 1.0000
R_{int}, R_σ	0.0365, 0.0281
Completeness of the data set	0.998
No. of measured reflections	24796
No. of independent reflections	4430
No. of parameters	228
No. of restraints	1
S (all data)	1.070
$R(F)$ ($I \geq 2\sigma(I)$, all data)	0.0290, 0.0306
$wR(F^2)$ ($I \geq 2\sigma(I)$, all data)	0.0708, 0.0717
Extinction coefficient	0.049(5)
Flack parameter x	–0.007(4)
$\Delta\rho_{max}$, $\Delta\rho_{min}$ / e·Å^{-3}	0.358, –0.476

6 References

[1] Nagai, T.; Wang, Y.; Hagiwara, K.; Inoue, M. Asymmetric synthesis of evoninic acid, *Tetrahedron Lett.* **2022**, *97*, 1–4.

[2] Zhang, D. W; Luo, Z.; Liu, G. -J.; Weng, L.-H. α N–O turn induced by fluorinated α-aminoxy diamide: synthesis and conformational studies. *Tetrahedron* **2009***, 65, 48*, 9997-10001.

[3] Speltz, T.E.; Fanning, S.W.; Mayne, C. G.; Fowler, C.; Tajkhorshid, E.; Greene, G.L.; Moore, T.W. Stapled Peptides with g-Methylated Hydrocarbon Chains for the Estrogen Receptor/Coactivator Interaction, *Angew. Chem. Int. Ed.* **2016**, 55, 4252-4255.

[4] Kochi, T.; Muto, K.; Kumagai, T.; Kakiuchi, F. Remote Arylative Substitution of Alkenes Possessing an Acetoxy Group via β-Acetoxy Elimination, *Angew. Chem. Int. Ed.* **2021**, 60, 24500-24504.

[5] Kolb, H. C.; VanNieuwenhze, M. S.; Sharpless, K. B. Catalytic Asymmetric Dihydrox-ylation, *Chem. Rev.* **1994**, *94*, 2483–2547.

[6] Kolb, H. C.; Sharpless, K. B. A Simplified Procedure for the Stereospecific Transformation of 1,2-Diols into Epoxides. *Tetrahedron* **1992**, *48*, 10515-10530.

[7] Lee, H.; Moon, S. J.; Yoo, Y. D.; Jeong, E. J.; Rho, J. R. Voratins A-C: Pyridinium Alkaloids from the Marine Dinoflagellate *Effrenium voratum* with Inhibitory Effects on Biomarkers for Benign Prostatic Hyperplasia. *J. Nat. Prod.* **2022**, *85*, 1495-1502.

[8] *APEX5*, Bruker AXS Inc., Madison, Wisconsin, USA, **2023**.

[9] *SADABS*, Bruker AXS Inc., Madison, Wisconsin, USA, **2016**.

[10] L. Krause, R. Herbst-Irmer, G. M. Sheldrick, D. Stalke, *J. Appl. Crystallogr.* **2015**, *48*, 3-10.

[11] G. M. Sheldrick, *Acta Crystallogr., Sect. A: Found. Adv.* **2015**, *71*, 3–8.

[12] G. M. Sheldrick, *Acta Crystallogr., Sect. C: Struct. Chem.* **2015**, *71*, 3–8.

[13] C. B. Hübschle, G. M. Sheldrick, B. Dittrich, *J. Appl. Crystallogr.* **2011**, *44*, 1281–1284.
